# Prediction of recurrence risk in endometrial cancer with multimodal deep learning

**DOI:** 10.1038/s41591-024-02993-w

**Published:** 2024-05-24

**Authors:** Sarah Volinsky-Fremond, Nanda Horeweg, Sonali Andani, Jurriaan Barkey Wolf, Maxime W. Lafarge, Cor D. de Kroon, Gitte Ørtoft, Estrid Høgdall, Jouke Dijkstra, Jan J. Jobsen, Ludy C. H. W. Lutgens, Melanie E. Powell, Linda R. Mileshkin, Helen Mackay, Alexandra Leary, Dionyssios Katsaros, Hans W. Nijman, Stephanie M. de Boer, Remi A. Nout, Marco de Bruyn, David Church, Vincent T. H. B. M. Smit, Carien L. Creutzberg, Viktor H. Koelzer, Tjalling Bosse

**Affiliations:** 1 Department of Pathology, Leiden University Medical Center, Leiden, The Netherlands; 2 Department of Radiation Oncology, Leiden University Medical Center, Leiden, The Netherlands; 3 Department of Computer Science, ETH Zurich, Zurich, Switzerland; 4 Department of Pathology and Molecular Pathology, University Hospital, University of Zurich, Zurich, Switzerland; 5 Swiss Institute of Bioinformatics, Lausanne, Switzerland; 6 Department of Gynecology and Obstetrics, Leiden University Medical Center, Leiden, The Netherlands; 7 Department of Gynecology, Copenhagen University Hospital, Rigshospitalet, Copenhagen, Denmark; 8 Department of Pathology, Herlev University Hospital, Herlev, Denmark; 9 Department of Radiology, Leiden University Medical Center, Leiden, The Netherlands; 10 Department of Radiation Oncology, Medisch Spectrum Twente, Enschede, The Netherlands; 11 Maastricht Radiation Oncology, MAASTRO, Maastricht, The Netherlands; 12 Department of Clinical Oncology, Barts Health NHS Trust, London, UK; 13 Department of Medical Oncology, Peter MacCallum Cancer Center, Melbourne, Victoria Australia; 14 Department of Medical Oncology and Hematology, Odette Cancer Center Sunnybrook Health Sciences Center, Toronto, Ontario Canada; 15 Department Medical Oncology, Gustave Roussy Institute, Villejuif, France; 16 Department of Surgical Sciences, Gynecologic Oncology, Città della Salute and S Anna Hospital, University of Turin, Turin, Italy; 17 Department of Obstetrics and Gynecology, University Medical Center Groningen, University of Groningen, Groningen, The Netherlands; 18 Department of Radiotherapy, Erasmus MC Cancer Institute, University Medical Center Rotterdam, Rotterdam, The Netherlands; 19 Wellcome Centre for Human Genetics, University of Oxford, Oxford, UK; 20 Oxford NIHR Comprehensive Biomedical Research Centre, Oxford University Hospitals NHS Foundation Trust, Oxford, UK; 21 Institute of Medical Genetics and Pathology, University Hospital Basel, Basel, Switzerland

**Keywords:** Probabilistic data networks, Endometrial cancer, Machine learning, Prognostic markers, Pathology

## Abstract

Predicting distant recurrence of endometrial cancer (EC) is crucial for personalized adjuvant treatment. The current gold standard of combined pathological and molecular profiling is costly, hampering implementation. Here we developed HECTOR (histopathology-based endometrial cancer tailored outcome risk), a multimodal deep learning prognostic model using hematoxylin and eosin-stained, whole-slide images and tumor stage as input, on 2,072 patients from eight EC cohorts including the PORTEC-1/-2/-3 randomized trials. HECTOR demonstrated C-indices in internal (*n* = 353) and two external (*n* = 160 and *n* = 151) test sets of 0.789, 0.828 and 0.815, respectively, outperforming the current gold standard, and identified patients with markedly different outcomes (10-year distant recurrence-free probabilities of 97.0%, 77.7% and 58.1% for HECTOR low-, intermediate- and high-risk groups, respectively, by Kaplan–Meier analysis). HECTOR also predicted adjuvant chemotherapy benefit better than current methods. Morphological and genomic feature extraction identified correlates of HECTOR risk groups, some with therapeutic potential. HECTOR improves on the current gold standard and may help delivery of personalized treatment in EC.

## Abstract (web summary)

A multimodal deep learning prognostic model based on histopathology outperforms current gold standards for identifying patients with endometrial cancer with different outcomes, in multiple external validation cohorts.

## Main

EC is the most common gynecological malignancy in high-income countries and is increasing in incidence^1^. Although most women with localized disease are cured by surgery, 10–20% develop distant recurrence^2^, which is typically incurable. Adjuvant chemotherapy can reduce this risk, at the expense of toxicity^3,4^. Thus, current guidelines recommend such adjuvant treatment based on a combination of clinicopathological risk factors (for example, histological subtype, grade, lymphovascular space invasion (LVSI), FIGO (International Federation of Gynaecology and Obstetrics) tumor stage) and, if available, the molecular classification of EC. The last identifies patients with favorable and unfavorable outcomes defined by *POLE* mutation (*POLE*mut) or p53 abnormality (p53abn), respectively, and intermediate outcomes characterized by mismatch repair deficiency (MMRd) or no specific molecular profile (NSMP)^5–8^. Recent efforts have been made to combine clinicopathological and molecular factors^9^; however, in practice, challenges remain as a result of the complexity of combining an increasing number of factors, high-interobserver variability in the assessment of histopathological factors, and costs and turnaround-times of molecular testing. In addition, histological slides contain lots of visual information, some with prognostic potential^10^, that is only partly captured in the grading and tumor histotyping by pathologists.

Deep learning (DL) models, including those using digitized hematoxylin and eosin (H&E)-stained tumor slides, have shown great promise in the prediction of molecular alterations^11–13^, cell composition^14^ and prognosis^15–21^, outperforming standard pathologist-based assessment. This is particularly true of the latest generation of self-supervised learning and whole-slide image (WSI) prediction DL models, which use attention-based networks^22^, graphs^15,19^ or (vision) transformers^23,24^ to provide more granular and interpretable image representation. In addition, multimodal DL models for prognosis prediction are promising to outperform unimodal approaches that solely rely on the morphological information provided by H&E WSIs^16,21^. We previously developed a DL model, image-based (im) four molecular classes in EC (im4MEC), to accurately predict the molecular EC classification from tumor H&E WSIs, and showed that image-based molecular classes predicted prognosis^11^. Others have classified EC binary recurrence^25^ or used uni-/multimodal DL models to predict EC overall survival^15,16,19,21^ (concordance indices (C-indices) of 0.629–0.687), but these have relied on more detailed tumor profiling, such as multiplex immunofluorescence staining^25^ or the combination of H&E WSIs with genomic and/or transcriptomic data^16^, neither of which is deliverable in clinical practice at present. Thus, there remains a pressing unmet need for a method that can predict EC distant recurrence from input data generated as part of routine clinical diagnostics.

**Fig. 1:**
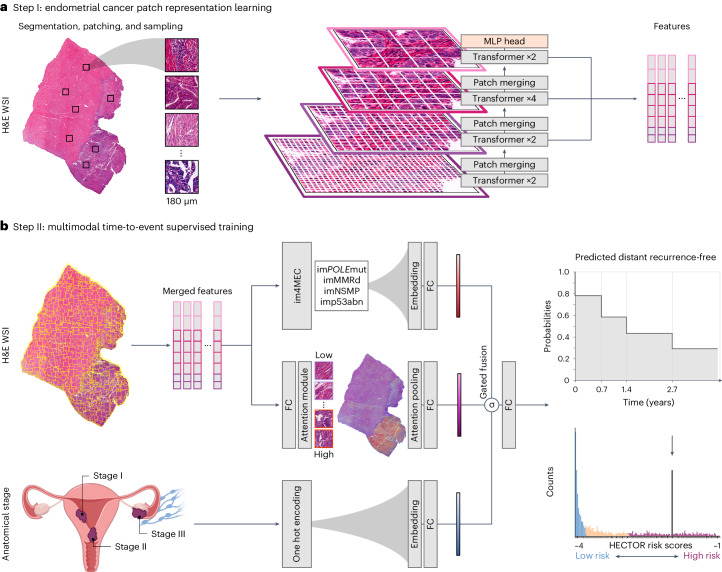
Overview of HECTOR. **a**, Tissue segmented from the H&E WSI of EC, subsequently patched at 180 μm. A multistage vision transformer^60^ was trained using self-supervised learning by randomly sampling patches from WSIs of 1,862 patients, excluding any patients of the internal and external test sets. Patch-level features are extracted from the last eight transformer blocks. **b**, HECTOR taking the H&E WSI and the (FIGO 2009) anatomical stage I–III category as inputs. Extracted patch-level features are spatially and semantically averaged. The patch features are passed into both an attention-based multiple instance learning model and the im4MEC DL model (with all layers frozen), which predicts the molecular class from the H&E WSI as im*POLE*mut, imMMRd, imNSMP or imp53abn^11^. Both the anatomical stage category and image-based molecular class are fed through the Embedding layers. Gating-based attention is applied on the resulting three embeddings^16,35^, followed by a Kronecker product for fusion. The −log(likelihood loss) was used to predict the distant recurrence-free probability function over discrete time^61^. Risk scores were defined as the integrated predicted probabilities. MLP, multilayer perceptron; FC, Fully Connected layer.

In the present study, we report the development and evaluation of HECTOR (Fig. 1)—a multimodal DL model to predict distant recurrence from H&E WSI and anatomical stage for postsurgical women with EC—across eight EC cohorts including three large randomized trials^3,26–31^.

## Results

### EC cohorts

HECTOR is a two-step DL model wherein the first step consists of self-supervised tumor image representational learning and the second of the distant recurrence prediction task (Fig. 1).

**Extended Data Fig. 1:**
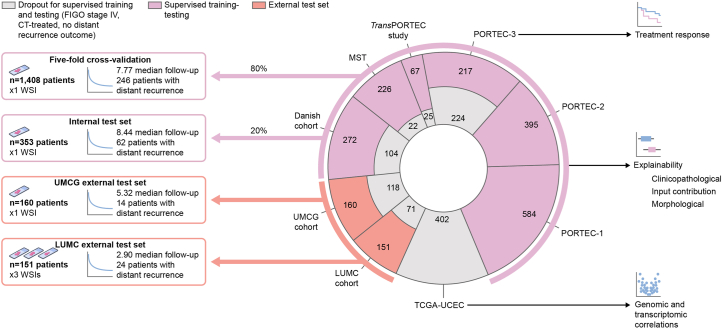
Overview of the data split and downstream analyses performed in this study. One representative WSI per patient from an Formalin-Fixed Paraffin-Embedded (FFPE) block was included. 20% of cases meeting inclusion criteria were randomly held out for an internal test set (*n* = 353). The remaining 80% was used for five-cross validation (*n* = 1,408 patients). This training dataset was enriched with dropped WSIs of FIGO 2009 stage IV cases or those with missing outcome such as the TCGA-UCEC cohort^21^ for training with self-supervised learning (*n* = 1,862). Two cohorts were held out as external test sets, the UMCG external test set (*n* = 160) and the LUMC external test set (*n* = 151). The LUMC external test set contains up to three FFPE blocks per case. More details for training and data split are provided in Methods. Altogether, including the two training steps and all downstream analyses, this comprehensive analysis comprised data of 2,751 tumors of women. CT, chemotherapy.

To train and validate the distant recurrence prediction task of HECTOR, we collected and curated tumor-containing, H&E-stained WSIs of the hysterectomy specimen and comprehensive clinicopathological datasets, molecular and clinical distant recurrence data for 2,072 patients with tumor stages (FIGO 2009) I–III EC across eight cohorts, including the PORTEC-1, -2 and -3 randomized trials^3,26–30^ (Extended Data Fig. 1; study CONSORT diagram shown as Supplementary Figs. 1 and 2 and Supplementary Tables 1 and 2). Of these, two population-based cohorts were held out as two external test sets: patients treated at the University Medical Center Groningen^31^ (UMCG; *n* = 160 patients) and the Leiden University Medical Center (LUMC; *n* = 151 patients) where the LUMC external test set also simulates a diagnostic scenario with up to three tumor blocks per patient. The remaining patients were divided randomly into a 20% held-out internal test set (*n* = 353) and 80% training set (*n* = 1,408) where fivefold crossvalidation was performed. The median duration of follow-up in the training set, internal test set, UMCG external test set and LUMC external test was 7.8, 8.4, 5.3 and 2.9 years, respectively, during which 246 (17.5%), 62 (17.6%), 14 (8.8%) and 24 (15.9%) patients had distant recurrence. Importantly, patients who underwent chemotherapy, predominantly the experimental treatment arm of the PORTEC-3 randomized trial (*n* = 225), were excluded from training because this treatment influences distant recurrence risk^3,4^ (Extended Data Fig. 1). These PORTEC-3 patients were, however, used for downstream analysis of adjuvant chemotherapy benefit by HECTOR.

To train HECTOR’s self-supervised learning step (which requires a large imaging dataset without outcome data), we enriched the training set with one additional cohort of the TCGA-UCEC^32^ (The Cancer Genome Atlas Uterine Corpus Endometrial Carcinoma) as well as the WSIs that were excluded for the distant recurrence task owing to cancer metastasized at diagnosis (FIGO 2009, stage IV) or missing outcome (*n* = 1,862; Methods).

Altogether, including the two training steps and the downstream analyses, the present study comprised tumor data from 2,751 patients.

### HECTOR design and performance

To design HECTOR and obtain the most performant DL model for prediction of distant recurrence based on the highest C-index^33^, we conducted ablation studies on the fivefold crossvalidation (Supplementary Table 3). HECTOR’s first step comprises a vision transformer for patch-level, self-supervised representational learning (Fig. 1a). HECTOR’s second step is a multimodal, three-arm architecture to predict distant recurrence-free probabilities (Fig. 1b). The three-arm architecture fuses prognostic information from the H&E-stained WSI of the tumor-containing uterine section, the image-based molecular class as predicted by im4MEC directly from the H&E WSI^11^ and the surgically assessed anatomical stage (as three-tiered based on the FIGO 2009 system, wherein stage I indicates a tumor confined in the uterus, stage II a cervical extent and stage III beyond, including vaginal, adnexal, pelvic and lymph nodes)^34^. To do this, we combined attention-based multiple instance learning with Embedding layers to map the discrete risk factors (the image-based molecular class and anatomical stage) to a higher-dimensional continuous vector space, with the importance of each factor controlled by gating-based attention^16,35^. Ablation studies (Supplementary Table 3) also included multitask learning^36^, with a second training objective predicting the image-based molecular class instead of the frozen im4MEC, or replacing attention-based multiple instance learning with DL models that integrate spatial information of the patches, such as transformer^23^ and attention-based graph neural network^15^. These two architectures did not outperform attention-based multiple instance learning for this task. Further details are provided in Methods and a summary of the HECTOR configuration is provided in Supplementary Tables 4 and 5.

**Fig. 2:**
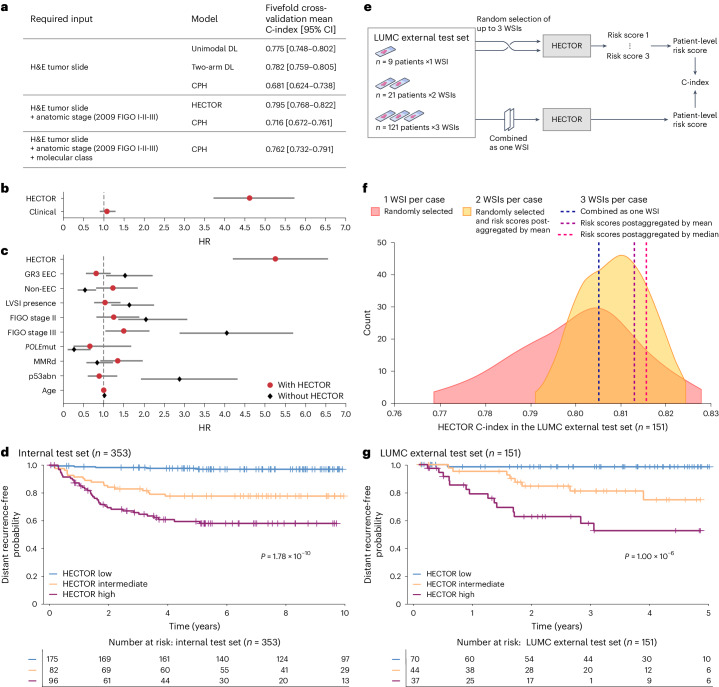
Performance of HECTOR. **a**, Comparison of HECTOR performance using the C-index with alternative unimodal and two-arm DL models and CPH models fitted on clinicopathological and molecular risk factors. **b**, Comparison of prognostic values between HECTOR and clinicopathological and molecular risk factors combined into one risk score in a multivariable analysis. Data are presented as the HRs and 95% CIs (*n* = 1,254 patients). **c**, Residual prognostic value of all established clinicopathological and molecular risk factors when using HECTOR-predicted risk scores in a multivariable analysis. Data are presented as the HRs and 95% CIs (*n* = 1,254 patients). **d**, The 10-year distant recurrence-free probability analysis using the Kaplan–Meier method by HECTOR risk groups in the internal test set and log rank test *P* value. **e**, Experiments conducted in the LUMC external test set (*n* = 151 patients) with the input of multiple WSIs. **f**, C-index of HECTOR in the LUMC external test set randomly using one to three WSIs for all patients and repeating the experiment 100×. **g**, The 5-year distant recurrence-free probability analysis using the Kaplan–Meier method by HECTOR risk groups when using up to three WSIs (postaggregated by median) in the LUMC external test set and log rank test *P* value. GR3, grade 3; EEC, endometrioid.

**Extended Data Fig. 2:**
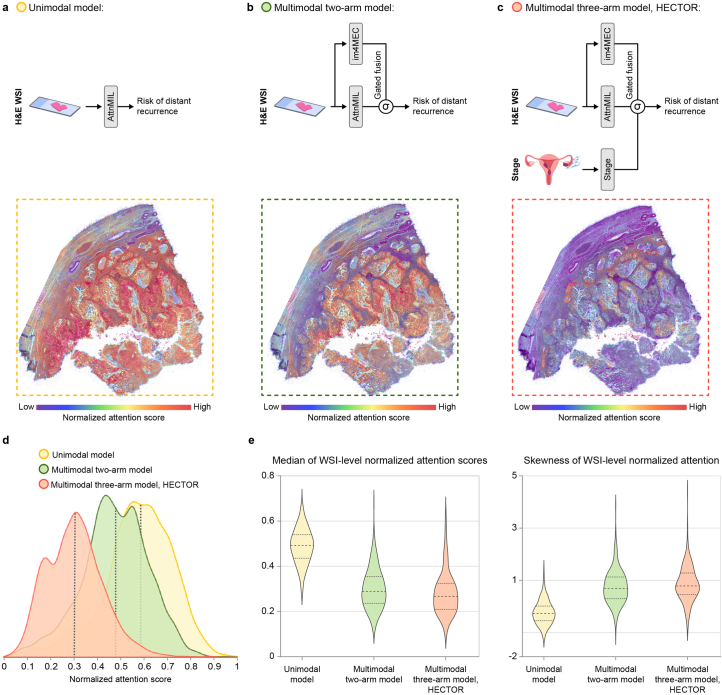
Shifts of attention scores from unimodal to multimodal model. **a**, Model using only H&E WSI (unimodal) and a corresponding example of the normalized attention scores shown as overlaid on the H&E WSI as a heatmap where red is high attention score and blue low attention score. **b**, The two-arm model with H&E WSI and image-based molecular class predicted by im4MEC, and a corresponding example of the normalized attention scores shown as overlaid on the H&E WSI. **c**, The multimodal three-arm HECTOR model with H&E WSI, image-based molecular class, and stage, and a corresponding example of the normalized attention scores shown as overlaid on the H&E WSI. **d**, Density plot of the normalized attention scores of the heatmap shown in a,b,c for each model. **e**, Quantitative analysis of the distribution shift between the three models in the internal test set (*n* = 353 patients) using the WSI-level skewness and median of the normalized attention scores.

HECTOR demonstrated a mean C-index of 0.795 (95% confidence interval (CI): 0.768–0.822) on fivefold crossvalidation. Notably, the addition of the image-based molecular class arm as predicted by im4MEC to the H&E WSI (referred to as two-arm or one-arm model, respectively) boosted performance from 0.775 (95% CI: 0.748–0.802) to 0.782 (95% CI: 0.759–0.805) with no need for extra input data. Adding the anatomical stage (as three-tiered FIGO 2009, stage I, II or III) further improved the C-index to 0.795 (95% CI: 0.768–0.822), yielding the final architecture of HECTOR (Fig. 2a). The cumulative area under the receiver operating curve (AUC)^37^ and integrated Brier score^38^ are reported in Supplementary Table 6. We also observed that HECTOR concentrated high attention to fewer regions while ignoring large parts of the H&E WSI compared with a model relying on the H&E WSI (Extended Data Fig. 2).

On the unseen internal test set, HECTOR obtained a C-index of 0.789 and, on the UMCG external test set, a C-index of 0.828. The performance in the LUMC external test set is depicted in ‘Performance with multiple WSIs’.

To aid clinical interpretation, we first defined categorical HECTOR risk groups as quartiles of the continuous risk scores in the training set. The groups from the first two quartiles were then combined for simplification because these had very similar clinical outcomes in the training set (distant recurrence-free probabilities of 98.1% and 95.8% by Kaplan–Meier analysis, respectively; Supplementary Fig. 3) and applied on to the internal and external test sets. Second, we computed the hazard ratio (HR) of HECTOR using a Cox’s proportional hazard (CPH) model with both continuous and categorical HECTOR risk scores as the independent variable and time to distant recurrence as the dependent variable.

HECTOR showed strong prognostic value as a continuous variable in the training test set (HR = 5.06; 95% CI: 4.35–5.89; *P* = 9.00 × 10^−99^), the internal test set (HR = 2.69; 95% CI: 2.07–3.49; *P* = 1.31 × 10^−13^) and the UMCG external test set (HR = 5.84; 95% CI: 3.06–11.14; *P* = 8.37 × 10^−8^). On the internal test set, 10-year distant recurrence-free probabilities for HECTOR low- (*n* = 175), intermediate- (*n* = 82) and high- (*n* = 96) risk groups were 97.0% (95% CI: 0.930–0.988), 77.7% (95% CI: 0.670–0.854) and 58.1% (95% CI: 0.469–0.677), respectively (log rank *P* = 1.78 × 10^−10^; Fig. 2d). The corresponding HR for HECTOR high- and intermediate-risk groups in the internal set, using the HECTOR low-risk group as the reference, were 15.63 (95% CI: 6.58–37.13; *P* = 4.81 × 10^−10^) and 7.67 (95% CI: 3.06–19.22; *P* = 1.37 × 10^−5^), respectively. In the UMCG external test set, a similar stratification was observed with 5-year distant recurrence-free probabilities for HECTOR low- (*n* = 102), intermediate- (*n* = 44), and high- (*n* = 14) risk groups of 93.9% (95% CI: 0.859–0.974), 91.4% (95% CI: 0.756–0.972) and 19.0% (95% CI: 0.0097–0.553), respectively (log rank *P* = 5.56 × 10^−10^; Supplementary Fig. 4). The corresponding HR for the HECTOR intermediate group in the UMCG external test set was 2.26 (95% CI: 0.61–8.42; *P* = 0.225) and in the high-risk group was 20.42 (95% CI: 5.92–70.50; *P* = 2.00 × 10^−6^), respectively.

### Comparison with current prognostic gold standard

We compared DL-based risk scores (that is, the one-, two-arm and HECTOR models) with the current standards for EC prognostication comprising clinicopathological risk factors and the molecular EC classification on the fivefold crossvalidation (Fig. 2a). For this, we first compared C-indices by type of input required: (1) a ‘base’ CPH model including variables defined by pathologists using H&E images alone (histological subtype, grade and LVSI); (2) the base model plus anatomical stage; and (3) the base model plus anatomical stage and molecular EC class. In the fivefold crossvalidation, given the H&E-based input data, the one- and two-arm model discrimination was superior to the base CPH model (C-index = 0.681; 95% CI: 0.624–0.738). HECTOR model discrimination was superior to the base CPH model plus anatomical stage which used the same inputs (C-index = 0.716; 95% CI: 0.672–0.761) and better or as good as the base CPH model plus anatomical stage and molecular EC class (C-index = 0.762; 95% CI: 0.732–0.791), which requires sequencing, immunohistochemistry (IHC) and expert pathology.

We further compared HECTOR prognostic values against current clinicopathological and molecular risk factors in multivariable analysis using HECTOR continuous risk scores as the independent variable. HECTOR retained prognostic values in multivariable models in which known risk factors (histological subtype, grade, LVSI, FIGO 2009 stage I–III, age, molecular class) combined as one risk score (referred to as the CLINICAL risk score) were not prognostic (HECTOR HR = 4.62 (95% CI: 3.72–5.73; *P* = 5.02 × 10^−44^) versus CLINICAL HR = 1.08 (95% CI: 0.90–1.30; *P* = 0.402)) (Fig. 2b). Similar multivariable analysis, including risk factors as individual variables, showed independent prognostic value of HECTOR (HR = 5.26; 95% CI: 4.21–6.56; *P* = 2.30 × 10^−48^), with only FIGO 2009 stage III disease retaining statistical significance (HR = 1.50; 95% CI: 1.05–2.14; *P* = 0.026) (Fig. 2c). Other known risk factors were no longer prognostic after inclusion of the HECTOR risk score, suggesting that these factors were captured by HECTOR. For instance, the *POLE*mut and p53abn molecular classes derived from ground-truth sequencing and IHC, respectively—HR = 0.66 (95% CI: 0.26–1.69; *P* = 0.384) and HR = 0.90 (95% CI: 0.61–1.34; *P* = 0.616)—and histological factors such as LVSI (HR: 1.05; 95% CI: 0.77–1.42, *P* = 0.776) would not be of additive prognostic value for the prediction of distant recurrence.

Given the current prognostic gold standards that would classify p53abn EC as high-risk tumors and MMRd and NSMP as intermediate-risk tumors with heterogeneous outcomes, we validated the capacity of HECTOR to refine prognosis within the MMRd, NSMP and p53abn molecular classes in the training and internal test sets. In particular, the HECTOR low-risk group also identified about 5.3% (16 out of 300) of p53abn EC cases with excellent prognosis in the entire dataset (Supplementary Fig. 5). Along these lines, we estimated the number of patients with markable different risk classification between HECTOR and the ESGO-ESTRO-ESP 2021 guidelines^5^ which combine clinicopathological and molecular factors (Supplementary Fig. 6). Among all patients with intermediate- to high-risk tumors based on the guidelines (and no report of distant recurrence), 48.2% (552 cases out of 1,146) of patients were predicted to be HECTOR low risk and 16.9% (62 cases out of 366) were predicted to be HECTOR low risk among high-risk tumors only. Among all guideline-based low-to-high intermediate-risk tumors, 11.2% (131 out of 1,170) of patients were predicted to be HECTOR high risk and 4.9% (14 out of 287) when restricting to only low-risk tumors.

### Performance with multiple WSIs

To evaluate the prognostic value and robustness of HECTOR in a second real-world external test set, we leveraged the fact that most cases in the LUMC cohort had multiple tumor-containing H&E WSIs derived from different tissue blocks per patient (121 of 151 cases had 3 WSIs, 21 had 2 and 9 had 1; Fig. 2e). This enabled us to validate the external performance of HECTOR in a diagnostic setting and subsequently test robustness to selection of the H&E WSI. The initial evaluation, using a HECTOR score derived from random selection of a single WSI per patient repeated 100×, demonstrated a mean C-index of 0.802 (95% CI: 0.799–0.804) for prediction of distant recurrence on the LUMC external test set (Fig. 2f).

HECTOR performance and risk stratification were slightly improved by the addition of further WSIs (taking per-patient HECTOR risk scores as either the mean or the median scores across WSIs) with C-indices of 0.810 (95% CI: 0.808–0.811) with up to 2 WSIs per patient, and 0.813 or 0.815 with up to 3 WSIs (Fig. 2f). A different method was tested wherein the WSIs were combined as one single input bag of images, yielding a C-index of 0.805. The 5-year distant recurrence-free probabilities using the median of HECTOR risk scores per patient were 98.4% (95% CI: 0.891–0.998) in HECTOR low risk (*n* = 70), 74.8% (95% CI: 0.534–0.874) in HECTOR intermediate risk (*n* = 44) and 52.6% (95% CI: 0.323–0.694) in HECTOR high risk (*n* = 37; log rank *P* = 1.00 × 10^−6^) (Fig. 2g and Supplementary Fig. 7). The corresponding HR (for the continuous HECTOR risk score) was 3.73 (95% CI: 2.34–5.96; *P* = 3.17 × 10^−8^) and (for the categorical high risk versus intermediate risk) 34.51 (95% CI: 4.52–263.39; *P* = 6.37 × 10^−4^) versus 15.08 (95% CI: 1.91–119.16; *P* = 0.010). Furthermore, HECTOR performance in patient stratification of the LUMC external test set extended to overall survival (5-year probabilities of 88.4% (95% CI: 0.769–0.944), 69.9% (95% CI: 0.468–0.845) and 47.0% (95% CI: 0.289–0.633) for low, intermediate and high risk, respectively; Supplementary Fig. 8).

Potential confounding by intratumoral heterogeneity also appeared to be minimal because 85 cases out of the 142 cases with more than 1 WSI had consistent HECTOR risk group predictions across the WSIs and only 3 cases with 3 WSIs had a different predicted HECTOR risk group for each WSI (Supplementary Figs. 9–12 and Supplementary Notes p16).

### Association with prognostic factors and input contribution

**Fig. 3:**
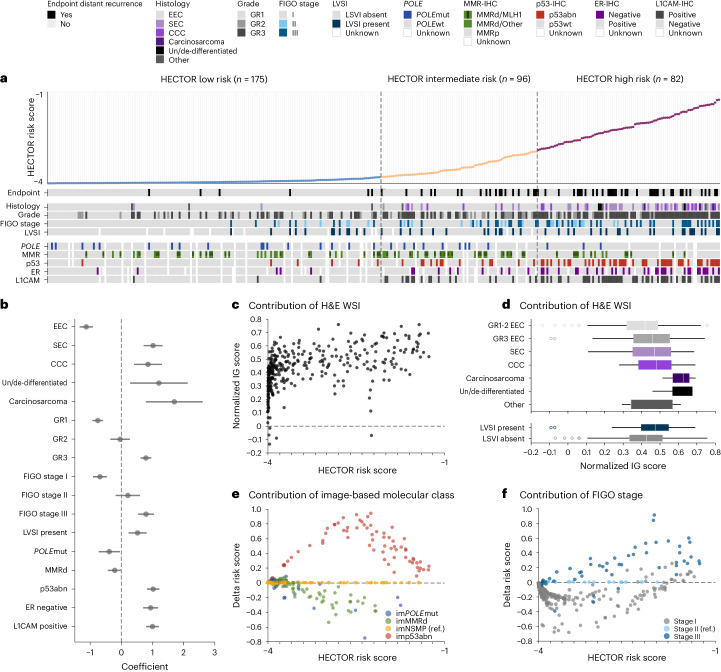
HECTOR explainability by analysis of HECTOR risk score with prognostic factors and analysis of input contribution. **a**, Heatmap of established prognostic factors for patients included in the internal test set (*n* = 353 patients) ordered by predicted HECTOR risk scores. Cases with multiple alterations in *POLE*, MMR and/or p53 are shown. Cases lacking any of these three specific molecular alterations are considered as NSMP according to the World Health Organization 2020 classification of female genital tumors^62^. **b**, Association of the prognostic factors and continuous HECTOR risk scores using multiple single linear regression with the HECTOR continuous risk scores as the dependent variable. Data are presented as the coefficients of the linear regression and 95% CIs (*n* = 353 patients). **c**, Analysis of the contribution to the HECTOR risk scores of the WSI modality in the internal test set (*n* = 353 patients), using the IG method^63^. The IG values of the patches were normalized and averaged by WSI. **d**, IG-normalized values of the WSIs stratified by histological subtypes (top) and presence of LVSI (bottom) in the internal test set (*n* = 353 patients). The box plots are defined by the center tick as the median value, the lower and upper parts of the box as the first (Q1) and third (Q3) quartiles, respectively, and the bounds of whiskers are (Q1 − 1.5 × IQR, Q3 + 1.5 × IQR) where IQR is the interquartile range (Q3 − Q1). Any outlier points beyond the whiskers are displayed with point marks. **e**, The contribution of the image-based molecular classes to the continuous HECTOR risk score in the internal test set, using the imNSMP as the reference (ref.) group. The difference in predicted risk score is computed between the risk score given by the image-based molecular class and the one produced by using imNSMP. **f**, The contribution of FIGO 2009 stage to the continuous HECTOR risk score in the internal test set, using FIGO 2009 stage II as the reference group. CCC, clear cell; GR1–3, grades 1–3; SEC, serous; wt, wild-type.

DL prognostic models may provide information on the correlates or features that determine clinical outcome. Initial analysis of the internal test set by multiple linear regression (Fig. 3a,b) revealed that lower HECTOR risk scores were associated with established favorable risk factors of endometrioid (EEC) histological subtype, grade 1 and *POLE*mut EC, and higher HECTOR risk scores with unfavorable factors, including non-EEC histological subtypes, grade 3, FIGO stage III, LVSI, p53abn EC, estrogen receptor negativity and L1 cell adhesion molecule (L1CAM) positivity (Supplementary Tables 7–9 and Supplementary Fig. 13). MMRd EC, grade 2 and FIGO 2009 stage II were spread throughout the risk score axis and were not statistically significant.

For deeper explainability, we evaluated the impact of the H&E WSI, im4MEC and anatomical stage on the prediction, that is, whether each modality decreased (negative contribution) or increased (positive contribution) the HECTOR risk scores of developing distant recurrence. We used the normalized Integrated Gradient (IG) values for the H&E WSIs, and differences in predicted risk scores with fixed value of im4MEC or FIGO anatomical stage for the same case in the internal test set. The H&E WSIs mainly had a positive contribution with values linearly increasing alongside HECTOR risk scores (Fig. 3c and Supplementary Fig. 14). We also noted higher magnitude of contributions toward grade 3 EEC or non-EEC histological subtypes and LVSI (Fig. 3d). Both observations may indicate that unfavorable morphological features captured in H&E WSIs are a strong driver of risk score predictions. The use of image-based molecular class and FIGO 2009 stage I–III was consistent with domain expertise in EC with im*POLE*mut and imMMRd mainly decreasing and imp53abn strongly increasing the HECTOR risk scores given accurate predictions (Fig. 3e, Supplementary Table 8 and Supplementary Fig. 15) and higher anatomical stage increasing the HECTOR risk scores (Fig. 3f and Supplementary Fig. 16).

These analyses enabled us to dissect data of the six patients with distant recurrence predicted as HECTOR low risk in the internal test set (Supplementary Table 10 and Supplementary Fig. 17). Experimental tests, in which the image-based molecular class was replaced by the true molecular class, showed no effect of misclassification by im4MEC in these instances on to the HECTOR risk group. Review of the single WSI input by an expert gynecopathologist revealed that, at least in two cases, WSIs were missing unfavorable visual features that were reported in the pathology report (substantial LVSI or high-grade tumoral areas). We also noted three cases predicted as HECTOR high risk with a *POLE* mutation. Although the same experiment confirmed that the image-based molecular class had little or no effect in the HECTOR predictions of these instances, these three cases all had notably FIGO 2009, stage II or III disease (Supplementary Table 11).

### Morphological correlates of outcome risk

**Fig. 4:**
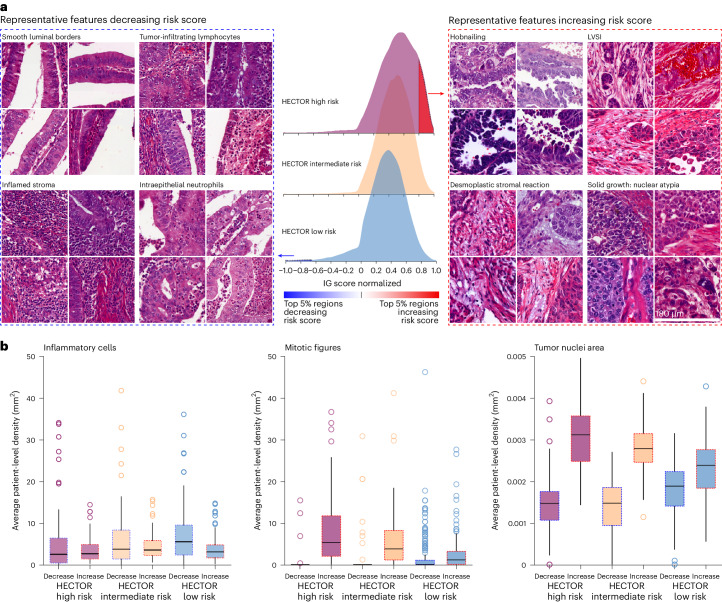
Morphological features contributing to HECTOR risk scores. **a**, The top 5% of the regions increasing and decreasing the risk score, from the IG method^63^, extracted for qualitative review and quantitative analysis. A representative selection of four patches for each morphological subtype (each selected from a different patient) showed the increasing risk score in the HECTOR high-risk group (right). A representative selection of four patches for each morphological subtype (each selected from a different patient) showed the decreasing risk score in the HECTOR low-risk group (left). Each patch is 180 × 180 μm^2^. **b**, Among the top 5% regions, decreasing and increasing the risk score, inflammatory cells, mitotic figures and the tumor nuclei area detected and computed with DL-based image analysis tools^14,64^. The average by patient is reported in the internal test set (*n* = 353). The box plots are defined by the center tick as the median value, the lower and upper parts of the box Q1 and Q3 quartiles, respectively, and the bounds of whiskers are (Q1 − 1.5 × IQR, Q3 + 1.5 × IQR). Any outlier points beyond the whiskers are displayed with point marks.

**Extended Data Fig. 3:**
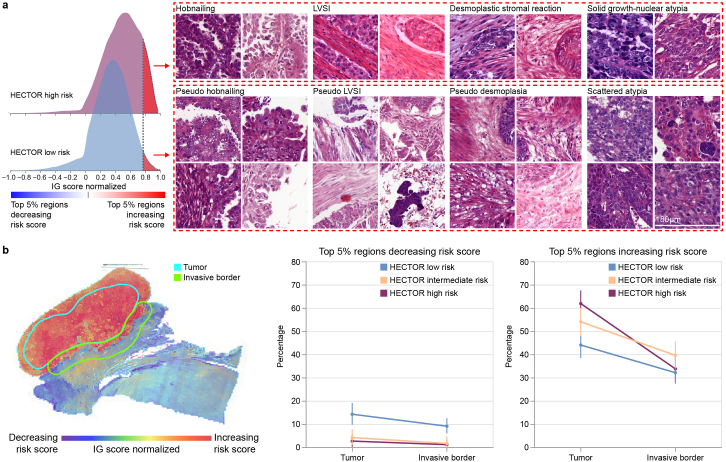
Morphological features increasing risk score in HECTOR high versus low risk group and quantitative spatial analysis. **a**, A representative selection of four patches for each morphological subtype (each selected from a different patient) increasing the risk score in the HECTOR low risk group as compared to the features increasing the risk score in the HECTOR high risk. Each patch is 180 × 180 μm. **b**, Spatial analysis of top 5% regions decreasing and increasing the risk score in all WSIs of the LUMC test set based on the manually annotated areas: tumor and invasive border. (left) An example showing the annotation of the tumor area and invasive border of one WSI and heatmap showing the contribution of the regions using the IG methods. (right) The relative contribution of these two annotated areas averaged by WSI shown for each HECTOR risk group. Data are presented as the mean values and standard deviation (*n* = 414 WSIs).

To identify the prognostic morphological features that may have been used by HECTOR, the top 5% regions of the H&E WSIs with the highest impact on the risk scores (decreasing and increasing) were extracted and reviewed by an expert gynecopathologist in the internal test set (Fig. 4a and Supplementary Figs. 18–22). Within the HECTOR low-risk group, the morphological features decreasing the risk score were identified as smooth luminal borders, inflamed stroma and intraepithelial lymphocytes, intraepithelial neutrophils and abundant compact normal myometrium without tumor. Morphological features increasing the risk score in the HECTOR high-risk group were a ragged luminal tumor surface (also referred to as hobnailing), LVSI, solid tumor growth with marked nuclear atypia, desmoplastic stromal reaction and the presence of mitotic figures (Fig. 4a). Within the HECTOR low-risk group, we observed morphological features with positive contribution, although relatively less common, as surface changes mimicking hobnailing, retraction artifacts mimicking LVSI, loose myometrium with edema mimicking desmoplasia and solid tumor growth with scattered high-grade nuclear atypia (Extended Data Fig. 3a).

Mitotic activity, inflammatory cell density and the size of the tumor nuclei were quantified using DL-based image analysis tools (Fig. 4b and Methods). More inflammatory cells were present in the top 5% regions decreasing the risk scores and this effect was more pronounced in the HECTOR low-risk group (*P* = 0.011). A higher mitotic density and larger tumor nuclei were found in the top 5% regions in the HECTOR high-risk group (both *P* < 0.001). These results remained consistent across image-based molecular classes and FIGO 2009 stages I–III (Supplementary Figs. 23–25) and when filtering in regions containing tumor cells (Supplementary Fig. 26). In a quantitative spatial analysis, we computed the overlap of the top 5% regions with the tumor and invasive border areas (Extended Data Fig. 3b). The latter showed that the regions increasing the risk scores were picked out more from the tumor than from the invasive border area. Tumor and invasive border areas contributed almost the same in regions decreasing the risk scores, notably in the HECTOR low-risk group.

### Genomic alterations, immune and transcriptional signatures

**Fig. 5:**
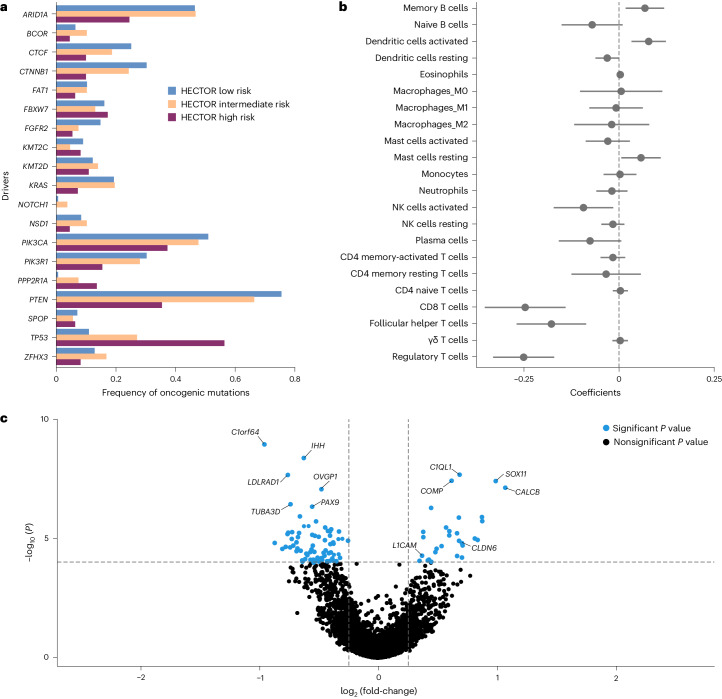
Genomic and transcriptomic correlations of HECTOR risk groups using TCGA-UCEC (*n* = 381). **a**, Analysis of the mutational frequency of the top 19 genes recognized as key oncogenic alterations in EC for each HECTOR risk group. **b**, Association of HECTOR risk score with the immune activation gene using multiple single linear regressions (Methods). Data are presented as the coefficients of the linear regression and 95% CIs (*n* = 381). **c**, Differential gene expression of HECTOR high-risk versus HECTOR low-risk TCGA-UCEC cases. *P* values of the likelihood ratio test were adjusted using the Benjamini–Hochberg FDR and statistical significance accepted <0.050.

For comprehensive analysis of the molecular correlates of HECTOR risk scores, we analyzed the TCGA-UCEC (*n* = 381 FIGO, stage I–III ECs) dataset (Fig. 5 and Supplementary Fig. 27). Coding driver mutations in *ARID1A*, *CTCF*, *CTNNB1*, *FGFR2*, *KRAS* and *PTEN* were enriched in the HECTOR low-risk group (all *P* < 0.005), whereas *PPP2R1A* and *TP53* mutations were more frequent in the HECTOR high-risk group (*P* = 2.19 × 10^−3^ and *P* = 2.81 × 10^−7^, respectively) (Fig. 5a and Supplementary Table 12). Using transcriptional data, we performed an analysis of CIBERSORT-defined lymphocyte populations using multiple linear regression (Fig. 5b). This revealed that increasing HECTOR scores were positively correlated with memory B cells (*P* = 0.008), activated dendritic cells (*P* < 0.001) and resting mast cells (*P* = 0.029), and inversely correlated with CD8^+^ T cells (*P* < 0.001), follicular helper T cells (*P* < 0.001), regulatory T cells (*P* < 0.001) and natural killer (NK) cell activation (*P* = 0.049). Notably, these associations were independent of EC molecular class and tumor mutational burden (TMB) (Supplementary Table 13). Further transcriptomic analysis (Fig. 5c, Supplementary Fig. 27c and Supplementary Table 15) confirmed that variation in lymphocyte populations was reflected in the differential expression of canonical immune cell markers, including *CD1C*, *BTLA* and *CD40LG* (enriched in the HECTOR low-risk cases). HECTOR high-risk tumors also demonstrated upregulation of genes predictive of worse outcomes in EC, including *L1CAM* and *CLDN6*, whereas HECTOR low-risk cases showed upregulation of genes associated with hormone signaling (*C1orf64* and *OVGP1*).

### Adjuvant chemotherapy response prediction by HECTOR

**Extended Data Fig. 4:**
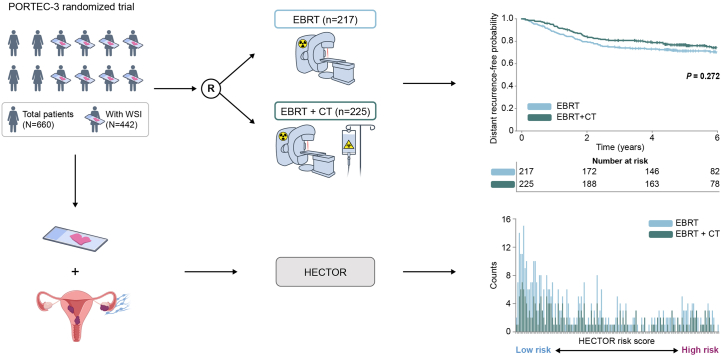
Overview of the PORTEC-3 randomized trial and analysis of treatment response prediction by HECTOR. In PORTEC-3, 660 evaluable patients were randomized (1:1) between adjuvant external beam radiotherapy (EBRT) alone and external beam radiotherapy in combination with concurrent and adjuvant chemotherapy (CT). For 442 patients whose WSI was available, HECTOR risk scores were inferred. HECTOR risk groups cutoffs were kept the same as the training set (Methods).

The investigation of whether HECTOR could predict the benefit of chemotherapy for distant recurrence risk was conducted using the PORTEC-3 randomized trial^3^. In this trial, patients with high-risk stage I–III EC were randomized to concurrent and adjuvant external beam radiotherapy with or without platinum- and paclitaxel-based chemotherapy. HECTOR risk scores were predicted on all PORTEC-3 cases for whom WSI was available (*n* = 442), which included the patients who underwent chemotherapy (*n* = 225). Importantly, these 225 cases had not been used in either training or test sets (Extended Data Fig. 4, Supplementary Table 14 and Supplementary Fig. 28). Analysis of distant recurrence-free probabilities by treatment arm and HECTOR demonstrated a statistically significant interaction between chemotherapy and HECTOR risk score as either a continuous or a categorical variable (*P*_INTERACTION_ = 0.014 and *P*_INTERACTION_ = 0.064, respectively).

**Fig. 6:**
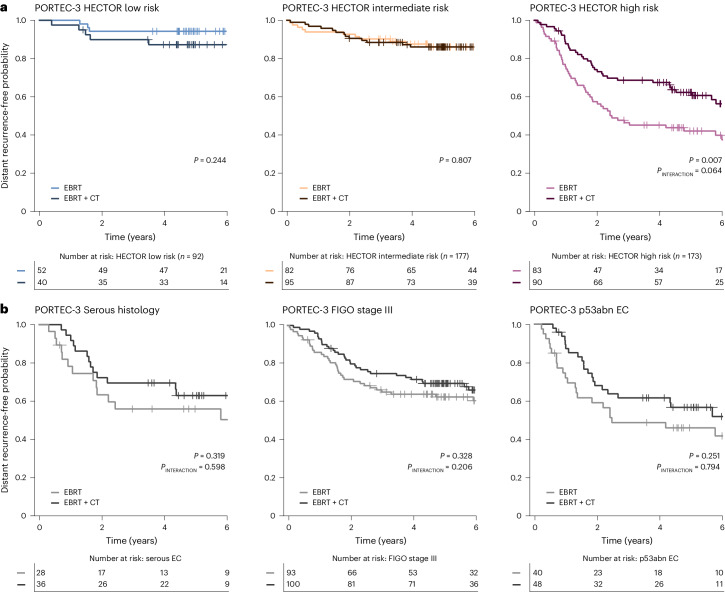
Impact of the addition of adjuvant chemotherapy to external beam radiotherapy on distant recurrence in the PORTEC-3 randomized trial by HECTOR risk group. **a**, The 6-year distant recurrence-free probability by Kaplan–Meier analysis and log rank test *P* value shown for each HECTOR risk group stratified by randomly allocated treatment. The *P* value of the interaction term using categorical HECTOR risk group is shown. There was also a significant interaction between the HECTOR continuous risk scores and the treatment (*P*_INTERACTION_ = 0.014). **b**, For comparison with HECTOR selection, distant recurrence-free probability by Kaplan–Meier analysis from the PORTEC-3 trial for different gold standard prognostic factors in EC relying on serous histology, the FIGO 2009 stage III and the p53abn molecular class is shown. The log rank test and interaction term *P* values are displayed. EBRT, external beam radiotherapy; CT, chemotherapy.

We examined this in detail across HECTOR risk groups (Fig. 6a). Within HECTOR low- (*n* = 92) and HECTOR intermediate-risk (*n* = 177) groups, outcomes were similarly favorable in both treatment arms, as evidenced by similar probability of EC distant recurrence (log rank *P* = 0.244 and 0.807, respectively). In contrast, among women classified as HECTOR high risk (*n* = 173), those who received adjuvant chemotherapy had significantly improved distant recurrence-free probabilities compared with those treated with external beam radiotherapy alone (5-year distant recurrence-free probability of 62.2% (95% CI: 0.511–0.715) versus 42.0% (95% CI: 0.311–0.526); log rank *P* = 0.007; HR = 0.561 (95% CI: 0.366–0.862; *P* = 0.008)). Exploratory analysis suggested that the predictive accuracy was greater than that provided by prognostic factors currently used to identify patients with high-risk tumors who were likely to benefit from adjuvant chemotherapy, including serous histological subtype, FIGO 2009 stage III and the p53abn molecular class (Fig. 6b). Further exploratory analyses suggested that HECTOR also identified patients who benefited from adjuvant chemotherapy within the NSMP and MMRd molecular classes (Supplementary Figs. 29 and 30). These results remained consistent when sub-stratifying by the image-based molecular class arm of HECTOR (Supplementary Fig. 31). Thus, HECTOR demonstrated significant predictive utility that may exceed that offered by current methods.

## Discussion

HECTOR, a DL model trained and validated in 2,072 patients with stage I–III EC^3,26–31^, with long-term follow-up, predicts postoperative distant recurrence risk using only H&E-stained tumor slide(s) of the hysterectomy specimen and anatomical stage. HECTOR obtained C-indices of 0.789, 0.828 and 0.815 in three unseen test sets for distant recurrence outcome. Its performance is on a par with clinically implemented prognostic DL tools in other cancer types (C-indices of 0.714 and 0.744 for colorectal cancer recurrence^39^, AUC of 0.78 for 10-year prostate cancer distant recurrence^40^) and also favorably compares with molecular prognostic assays such as OncotypeDX (C-index of 0.641 for 10-year breast cancer distant recurrence^41^). Notably, HECTOR outperformed the current diagnostic gold standard of combined pathological and molecular analysis for distant recurrence risk prediction, and was also found to be predictive of adjuvant chemotherapy benefit in the PORTEC-3 randomized trial^3^. Pending prospective validation, our results suggest that HECTOR may have the potential to be a highly effective tool for individualized prognostication of women with EC, while delivering shorter turnaround times and reducing testing costs. HECTOR may also enable biomarker discoveries for improving targeted treatment decision-making.

HECTOR performance is the result of a new multimodal, integrative, three-arm architecture which leveraged prognostic information from the H&E WSI, the image-based molecular class from im4MEC^11^ and anatomical stage^34^. This multimodal architecture outperformed alternative DL models using only H&E-based information, corroborating other studies^16,42^. It is interesting that nesting of the im4MEC model within HECTOR boosted the performance, in contrast to other studies where integration of copy number variation or transcriptomics did not improve prediction of overall survival in EC^16^. We demonstrated that the prognostic value of categorical clinical risk factors, such as the anatomical stage, can be learned end to end by the DL model to increase predictive accuracy. HECTOR takes a step toward integrating patient-level imaging, image-based molecular and clinical insights, which may benefit similar studies in other cancer types where unimodal DL models have been developed on images only^17,20,39^.

Our preliminary investigations of model explainability and risk score correlates offer good prospects to improve our understanding of the biology of EC and other cancer types. For example, the association of HECTOR low-risk scores with immune cell infiltrate is consistent with data showing better prognosis of immune-infiltrated EC^10^, although at present it is unclear whether HECTOR directly quantified lymphocyte subtypes such as T cells from H&E WSIs. The upregulation of *CLDN6* in HECTOR high-risk ECs is consistent with this being a predictor of distant recurrence^43^. Cases with combined HECTOR high risk and *CLDN6* upregulated could be actionable as a chimeric antigen receptor T cell target^44^. Although desmoplastic stromal reaction is known to predict bad prognosis in colorectal cancer, the association that we describe in the present study has not previously been reported in EC^45^. Whether this represents a morphological readout of *L1CAM* overexpression^46^ is presently unclear. We also confirmed well-established, unfavorable histopathological risk factors in EC aligning with higher HECTOR risk scores^5^. Thus, we expect the outperformance of standard histopathology by HECTOR probably being driven by the nonlinear combination of each factor and, more importantly, the noncategorical processing of the visual information from the WSIs.

HECTOR’s design holds considerable promise for scaling to clinical implementation because it is built on two broadly available and cost-effective inputs routinely obtained in diagnostics: one H&E-stained tumor slide from which we used the image-based rather than the true molecular classes and high-level clinical information of the tumor extension at diagnosis (to the cervix or beyond the uterus excluding distant) which is independent of an evolving FIGO staging system^9^. After appropriate validation in a prospective clinical trial setting, HECTOR may have great potential to individualize triage of women with EC in the adjuvant setting from low to high risk of distant recurrence. Subsequent treatment decision-making by clinicians could be guided accordingly because HECTOR low-risk prediction could provide a means to de-escalate adjuvant treatment or to encourage adjuvant systemic therapy recommendation for patients predicted to be HECTOR high risk (such as chemotherapy^3,4^ or targeted therapies in clinical trials^47–49^). The therapeutic guidance within HECTOR high risk can be supported by selective targeted molecular testing such as MMRd or even DL-based molecular predictions given a good accuracy^11^. Although our data support that HECTOR could reduce under- and over-treatment for women with EC, it would also spare challenges and expenses of resource-limited environments where molecular testing and expert pathologist review are difficult or not feasible. We speculate that future technical improvements of HECTOR could be an extension of its inputs to consecutive digitized H&E-stained hysterectomy sections followed by three-dimensional reconstruction^50^, routinely performed IHC-stained WSIs^51^, preoperative radiology images^52^ or a clinical report encoding patient-level clinical information^53^. Moreover, DL-based assessment of the anatomical stage by leveraging histology images of the cervical, ovarian and (or radiology images of) lymph node sections would make HECTOR independent of pathology review.

Our study has several strengths. Our total cohort of 2,751 patients, including 3 randomized trials, makes this one of the largest DL-based prognostic studies in EC performed to date. Our state-of-the-art multimodal DL methodology allowed us to leverage prognostic information from multiple factors, including those beyond the H&E image alone. Expert pathology review and molecular profiling enabled us to benchmark our methodology against the current gold standard in risk stratification of EC. Limitations of our study are that our current model based on multiple instance learning is unaware of the spatial relationship between regions and was not designed to leverage information between multiple WSIs, both of which may improve performance^54,55^; although context-aware architectures have not been found to improve performance in this task. In addition, complex interactions of the morphology, molecular and anatomical stage may be further optimized by experimenting with other early-to-late fusion techniques^42^, or learning more generalizable morpho-molecular representations using pretext tasks. Some patients in the study did not undergo surgical staging lymphadenectomy^26,27^, a consideration that may have introduced some noise in the anatomical stage input and may explain the residual prognostic value of advanced disease stage III in multivariable analysis. Given that *POLE*mut EC mutations rarely metastasize^56^, we acknowledge the possibility that the risk may be overestimated in these rare instances by HECTOR. Furthermore, not all morphological correlates observed in the H&E regions (for example, structural changes) were quantified in the present study owing to the lack of available labeled datasets that could have been used for training DL-based, EC-specific image analysis tools. Importantly, HECTOR performance needs further validation both in unselected cohorts more diverse than the ones of largely European ancestry that we examined and in prospective trials. As such, prospective validation will be conducted first in the PORTEC-4a trial^57^. Moreover, as the therapeutic landscape of EC is rapidly evolving, the most suitable adjuvant systemic therapy for HECTOR high-risk patients needs to be continuously validated^4,58^ or (prospectively) explored in other randomized trials^47–49,59^.

In summary, validation and extension of HECTOR could help delivery of precision medicine to advance prognostication of women with stage I–III EC who underwent primary surgery, with improvement worldwide on both systemic therapy recommendation and treatment de-escalation.

## Methods

### Ethics statement

The PORTEC-1, PORTEC-2 (NCT00376844) and PORTEC-3 (NCT00411138) study protocols were approved by the Medical Ethical Committee Leiden, Den Haag, Delft and the medical ethics committees at participating centers. Studies were conducted in accordance with the principles of the Declaration of Helsinki. Ethical permissions for the retrospective use of the clinical trials and retrospective cohorts (TransPORTEC study, Medisch Spectrum Twente (MST)) were obtained by the Medical Ethical Committee Leiden (nos. B21.065 and B21.011), as well as the LUMC cohort (nWMO‐D4‐2023‐002) and the Danish Cohort by the Center for Regional Udvikling, De Videnskabsetiske Komiteer (H-16025909). All study participants of the clinical trials provided informed consent. The ethical boards have provided a waiver for informed consent for the other studies. For the UMCG cohort, the medical ethical committee granted permission for the use of the data and provided a waiver for informed consent owing to the observational nature of the study.

### Cohorts

We used formalin-fixed paraffin-embedded (FFPE) tumor material and clinicopathological data of patients with EC from three randomized trials and six clinical cohorts. We included study participants of the female sex, independent of gender identity.

The PORTEC-1 trial recruited 714 women with early stage intermediate-risk EC from 1990 to 1997, and after primary surgery, randomly assigned to pelvic external beam radiotherapy or no adjuvant treatment^26^. The PORTEC-2 trial randomized 427 women with early stage, high- to intermediate-risk EC between 2000 and 2006 to external beam radiotherapy or vaginal brachytherapy^27^. The PORTEC-3 randomized trial included 660 women with stage I–III high-risk EC from 2006 and 2013, and randomly allocated them to pelvic external beam radiotherapy alone or external beam radiotherapy combined with concurrent and adjuvant chemotherapy^3^. The retrospective TransPORTEC study included 116 high-risk EC tumors from international patients using the same inclusion criteria as the PORTEC-3 from 5 institutions (LUMC and UMCG, the Netherlands; University College London and St Mary’s Hospital, Manchester, UK; and Institute Gustave Roussy, Villejuif, France)^28^. The prospective cohort of MST included 257 patients with stage I–III high-risk EC, with the same inclusion criteria as PORTEC-3, who were treated between 1987 and 2015 at MST, Enschede in the Netherlands^29^. The Danish cohort consisted of 451 patients with high-grade EC who were prospectively registered in the Danish gynecological cancer database^30^. The UMCG cohort is a population-based cohort consisting of patients treated at the UMCG between 1984 and 2004, that is, 278 patients with follow-up data collected until 2010 (ref. ^31^). The LUMC cohort is a retrospectively collected, population-based cohort of 222 patients diagnosed and treated at the LUMC between 2012 and 2021. Finally, the publicly available TCGA-UCEC cohort^32^ of 529 patients was downloaded from the cBioPortal^65,66^.

### Datasets

One representative H&E-stained slide of the hysterectomy specimen was included for each patient depending on the availability of the tumor material (Supplementary Figs. 1 and 2, and Supplementary Tables 1, 2 and 14). For the LUMC cohort, we collected three diagnostic H&E-stained tumor slides per patient case with EC, each from a different FFPE tumor tissue block. H&E slides were scanned at ×40 magnification using two scanners 3Dhistech P250 (resolution 0.19 µm per pixel) and 3Dhistech P1000 (resolution 0.24 µm per pixel). Any image provided in the manuscript is an unprocessed scan. Qualitative review was conducted on all WSIs by our expert pathologist, after which cases with no tumor, poor tissue quality and out-of-focus scanning issues were excluded, yielding 2,560 cases with at least one WSI per case (CONSORT chart in Supplementary Figs. 1 and 2).

In the present study, some cases were excluded from the supervised training of HECTOR based on the following criteria: (1) missing time to distant recurrence follow-up data, (2) FIGO 2009 stage IV^34^ because they already have distant recurrence at time of diagnosis and (3) treatment with adjuvant chemotherapy because it may have lowered the risk of distant recurrence^3,4^. The categorical anatomical stages I, II and III are defined following the FIGO 2009 classification^34^. Hence, it represents a tumor confined in the uterus (stage I), a tumor spread to the cervical stroma (stage II) or to the vagina, adnexa, pelvis and lymph nodes (stage III) at diagnosis. Distant recurrence in the adjuvant setting was defined as any recurrence outside the pelvis. Hence, distant recurrence included abdominal metastasis and para-aortic lymph node metastasis. Time to distant recurrence was defined to start at randomization (for PORTEC-1, -2 and -3) or date of primary surgery (MST, TransPORTEC study, Danish, UMCG and LUMC cohort) and to end at the date of the diagnosis of metastasis, or the date of last follow-up or death in patients without metastasis. We also stress that adjuvant chemotherapy was not the standard of care at the time the clinical cohorts were collected and that the vast majority of patients treated with adjuvant chemotherapy originated from the PORTEC-3 randomized trial (*n* = 225).

Following the aforementioned criteria, 2,072 cases were included for the supervised train–test split: 584 from PORTEC-1 (ref. ^26^), 395 from PORTEC-2 (ref. ^27^), 217 from PORTEC-3 (ref. ^3^), 67 from the TransPORTEC study^28^, 226 from the MST cohort^29^, 272 from the Danish cohort^30^, 160 from the UMCG cohort^31^ and 151 from the LUMC cohort. Then we held out one internal test set and two external test sets, all representing an unselected population. The internal test set was obtained by randomly sampling 20% of the supervised training set, stratified by discrete time intervals and censorship status to ensure the presence of enough events across time (*n* = 353, of which 116 were from PORTEC-1, 100 from PORTEC-2, 43 from PORTEC-3, 13 from the TransPORTEC study, 35 from the MST cohort and 46 from the Danish cohort; median follow-up of 8.45 years with 62 events). The first external test set is the UMCG cohort (*n* = 160 patients; 5.32-year median follow-up time with 14 events). The second external test set is the LUMC cohort (*n* = 151 patients: 121 with 3 WSIs, 21 with 2 WSIs and 9 with 1 WSI; 2.90-year median follow-up time with 24 events). Finally, the remaining 1,408 WSIs were used for supervised training of HECTOR (468 from PORTEC-1, 295 from PORTEC-2, 174 from PORTEC-3, 54 from the TransPORTEC study, 191 from the MST cohort and 226 from the Danish cohort; median follow-up of 7.77 years with 246 events).

In addition, the HECTOR risk scores were predicted on the previously excluded, chemotherapy-treated cases from the PORTEC-3 randomized trial^3^ (*n* = 225), as well as the patients with stages I–III from TCGA-UCEC (*n* = 381).

For the self-supervised learning, we used only the 1,408 WSIs already reserved for supervised training, and thus strictly limited to only those that were not part of the internal and external test sets. In addition, the self-supervised learning training was enriched by cases with any stage of disease, whose treatment or distant recurrence outcome data were unknown (*n* = 454 of which 31 from the TransPORTEC study, 5 from the MST cohort, 16 from the Danish cohort and 402 from TCGA-UCEC), resulting in 1,862 cases for self-supervised learning.

### Performance evaluation

Hyperparameter optimization and model comparisons (including architecture choices for patch representational learning with self-supervised learning) were evaluated on the supervised downstream task guided by the C-index metric^33^ (using a tau = 10 years and scikit-survival Python package (v.0.17.2)). To this end, a fivefold crossvalidation routine was performed on the 1,408 WSIs reserved for supervised training. The most performant architecture and hyperparameters were selected based on the highest mean C-index over the five folds. The final model, referred to as HECTOR, is then retrained on the full training set and evaluated on to the internal and the two external test sets (UMCG and LUMC). The cumulative AUC^37^ and Brier scores^38^ were additionally computed.

Given the fact that the LUMC external test set contains up to three WSIs per case, as opposed to one in the internal test set and the UMCG external test set, we performed multiple experiments to derive patient-level risk scores using random sampling. First, we randomly selected one WSI per case and repeated this experiment 100×, yielding a mean C-index and CI. Second, we randomly selected up to two WSIs for each case when available, then averaged with the mean the two risk scores per patient and repeated it 100×. Third, we selected all available WSIs of the external test set with up to three WSIs per case when available and computed the mean and median of the two or three risk scores. In an additional experiment, we combined each patient’s WSIs by merging the patch features from all available WSIs into a single feature bag.

### WSI preprocessing

WSI segmentation was performed using Otsu thresholding. Nonoverlapping patching was performed at 180 µm and patches were resized to 256 × 256 pixels^2^. On average, this procedure generated a bag of 10,185 patches per WSI.

### Vision transformer-based patch representational learning

We followed advancements in self-supervised learning by adopting vision transformer-based DL models that are capable of learning fine-grained, patch-level representation at multiple resolutions. For this, we trained EsVIT^60^ and compared it with CtransPath^67^, an alternative model trained on the histopathology domain (Supplementary Table 3). We modified the initial proposed four-stage Swin^68^, transformer-based architecture of EsVIT to capture cell- and region-level tissue information and to fit our computational resources. The patch size of stage 1 was doubled to 8 pixels to reduce the sequence length and increase field of view to capture cell views. In stages 2–4, we kept the two-factor feature map merging rate and resized the input images to 256 × 256 pixels^2^ instead of 224 × 224 pixels^2^ to avoid indivisible patch size at stage 4. Finally, the number of stacked transformers in stage 3 was reduced from six to four and the rest were kept to two. The first embedding dimension remained unchanged at 96 and the number of attention heads by stage was also kept unchanged, that is, 3, 6, 12 and 24 (Supplementary Table 4).

A dataset of 3,702,447 patches was curated by randomly extracting up to 2,000 patches per WSI at 180 µm resized to 256 × 256 pixels^2^ from the 1,862 WSIs appointed for self-supervised learning. Thereafter, the modified EsVIT was trained on 3 Nvidia RTX 8000 GPUs (graphic processing units) with a batch size of 128 for 100 epochs with a window of 14 to encourage learning of long-term dependencies between patches. For performance improvement, we also used the view- and region-level prediction DINO (self-distillation with no labels) heads with no weight normalization and frozen layers at first epoch and the default output dimension of 65,536 (ref. ^60^). We followed the EsVIT authors’ recommendations with a smaller batch size by increasing the momentum teacher to 0.9996 and starting with the initial teacher temperature of 0.04. The teacher temperature was adjusted halfway through training from 0.04 to 0.02 for further loss decrease. We optimized with AdamW and default parameters, default optimization routines of the learning rate (linear warm-up for ten epochs followed by cosine scheduler to 1 × 10^−6^) and weight decay (cosine scheduler from 0.04 to 0.4). The data augmentation was used exactly as done in the original publication^60^.

After the training was completed, the patch-level features were extracted from the attention heads of the stacked transformers at each stage. For our downstream task, we observed an improvement by extracting the last 8 blocks compared with the default last 4 mentioned in the publication^60^, yielding feature vectors of size 3,456 (Supplementary Table 3).

### Multimodal DL prognostic model

To build the multimodal model for distant recurrence prediction task, ablation studies were first performed using the H&E WSI modality only (referred to as H&E-based, one-arm model) followed by integrating the image-based molecular classes derived from the H&E-based predictions of im4MEC^11^ (referred to as two-arm model) and the categorical stage (hence referred to as HECTOR). This section describes HECTOR with Supplementary Table 5 summarizing the architecture and training parameters, whereas ‘Ablation studies’ provides further details about some training experiments and the choice of the architecture.

The H&E-based, one-arm model takes as input the bag of 180-µm patch-level features of size 3,456 extracted from EsVIT^60^, where the number of patches per bag varies. To train toward time-to-event data and given a batch size of one of the attention-based multiple instance learning (AttentionMIL) model, the time scale was discretized into four intervals based on the quartiles of the distribution of uncensored patients and the −log(likelihood loss) was used^61^.

Within the AttentionMIL model, we reported a slight performance increase by adding another WSI preprocessing step. Specifically, WSI morphological information was spatially and semantically compressed by averaging highly correlated, nearby patch-level features using a L2 norm threshold of three patches and a cosine similarity of 0.8. This step reduced the bag of features from 10,185 patches on average to 1,723 at 180 µm (Supplementary Table 3). Each mean patch-level feature is compressed by 3 Fully Connected layers gradually down to 512. The attention module computes attention scores on latent features reduced to 256 before pooling, resulting in a slide-level embedding of size 512.

To leverage the well-established prognostic value of the molecular class (here image-based derived from the H&E-based predictions of im4MEC^11^) and the categorical (FIGO 2009) stage I, II and III variable, and given the AttentionMIL model computes an H&E slide-level embedding from the patches, we experimented with intermediate-to-late fusion to integrate slide-level, image-based molecular class and patient-level anatomical stage information at the H&E slide-level embedding. We proposed an approach of first encoding each categorical risk factor to higher-dimensional vector space with a learnable Embedding layer of size 16 followed by Elu activation function and one Fully Connected layer of size 8. Next, a gating-based attention mechanism with bilinear product was applied on the embeddings from different modalities to weight the importance of each modality based on ref. ^16^. To capture all interactions and retain unimodal embeddings, one was appended to the attention-weighted embeddings and then fused using the Kronecker product^35^. It is important to note that, for using the image-based molecular class as an input modality for HECTOR, we retrained the im4MEC model on the training set specifically designed for the present study. This was done to avoid any information leakage because some cases used for training the original im4MEC model were used as testing on validation in the present study.

The final multimodal embedding was further reduced by using two Fully Connected layers of size 256 and 128 before the survival categorical head of a Fully Connected layer with output size as the number of discrete time intervals. Each Fully Connected layer in the architecture was followed by a dropout of 0.25 and a ReLU activation function.

HECTOR was trained for 24 epochs with an initial learning rate of 3 × 10^−5^ decayed by a factor of 10 at epochs 2, 5 and 15. The Adam optimizer was used with default parameters and a weight decay of 1 × 10^−5^. HECTOR was also developed by adapting sections of open access repositories^11,16,21^.

### Ablation studies

To find first the optimal architecture to predict distant recurrence from the H&E modality (one-arm model), three state-of-art WSI classification architectures were adapted to our distant recurrence prediction task: AttentionMIL^22^, a Graph Attention Network following ref. ^15^, with a radius up to 32 connected patch nodes and a transformer architecture following ref. ^23^. Both of these architectures were adapted from their open access repository. They were both trained on the same feature bags extracted using EsVIT with a batch size of one and the same discrete survival loss (−log(likelihood loss)). We found that the AttentionMIL architecture yielded a higher C-index than the Graph Attention Network and the transformer in this prognostic task while featuring far lower computational complexity (Supplementary Table 3), which corroborates the findings of ref. ^15^ for TCGA-UCEC.

To incorporate the image-based molecular class predicted by im4MEC from the H&E WSIS, experiments included: (1) transfer learning in which the AttentionMIL backbone was pretrained toward the molecular class and subsequently fine-tuned on the prognostic task; (2) multitask learning in which a second training objective was added to predict the image-based molecular class in addition to the prognosis; and (3) fusion of the image-based molecular class derived from the frozen im4MEC model (as extracted from either an intermediate layer or the final predicted categorical class, followed by an Embedding layer and attention gate). In experiment 2, a second classification head was implemented which was trained using the weighted sum of the survival loss (−log(likelihood loss)) and the cross-entropy classification loss. The weight factor was considered as a hyperparameter and was optimized using the fivefold crossvalidation. Experiment 3 which consisted of the inclusion of the predicted categorical class using an Embedding layer and attention gate resulted in the highest mean C-index (Supplementary Table 3).

Experiments around fusing the stage category included notably training with the extended FIGO 2009 taxonomy or a reduced three-class taxonomy (I, II and II) followed by an Embedding layer and attention gate, the latter achieving the highest C-index (Supplementary Table 3).

### Association with clinicopathological data analysis

We performed multiple single linear regression analyses using the HECTOR continuous risk scores as the dependent variable and the clinicopathological data as the regressor. Statistical tests were two sided with statistical significance accepted with *P* values <0.050. Regression coefficients and exact *P* values have been reported in Supplementary Table 7.

### Input contribution

The IG method^63^ was used to measure the contribution of the WSI and to identify the patches within a WSI relevant to the prediction of the hazard function. Given the discrete time intervals, IG scores were averaged over the four neuron targets. The IG baseline for feature missingness was represented as patch-level features derived from white patches. All IG scores were patient-wise normalized between −1 and +1 while maintaining the sign and the IG score of zero, and further averaged to get a WSI-level IG score. Positive IG value toward 1 means that it contributed positively to increase the risk score, whereas negative means it contributed to decrease the risk score. Selection of representative patches was performed once by an expert pathologist within the top 5% patches, increasing and decreasing the risk scores for each case.

The contribution of the predicted image-based molecular class by im4MEC and the FIGO stage was calculated by fixing the stage- and image-based molecular class values with the value of our choice (referred to as the ‘reference group’) followed by computing the difference in predicted risk scores. Similar to the IG method, a positive or negative difference means a positive or negative contribution to the risk score, respectively.

### Cell-level composition

As part of the explainability section of HECTOR to quantify visual features of extracted patches with high contribution, we first used the cell segmentation and classification Hover-Net^14^ DL model to obtain inflammatory cell counts, retrained on EC-specific WSIs^11^. Then, mitotic figures were detected with a pan-cancer DL-based detector^64^ that was fine-tuned on EC tissue for the purpose of the present study. Fine-tuning was performed by extending the original training set^69^ with additional data points that we internally annotated in 10 WSIs from the PORTEC datasets selected to cover the variability of EC histological types. Region-level inflammatory and mitotic activity density were defined as absolute count normalized by the area in square millimeters and further averaged over the number of regions to obtain a patient-level density value. The size of tumor nuclei was reported in mm^2^ and averaged by patient. The statistical association between the HECTOR risk scores and the patient-level quantity of visual features was tested with linear regressions within the regions of interest, that is, the regions with either a negative or a positive contribution. Statistical tests were two sided with statistical significance accepted for *P* values <0.050. The coefficients of linear regressions and exact *P* values were the following: coefficient −0.0109 (95% CI: −0.019 to −0.002), *P* = 0.011, for the patient-level inflammatory density within the negative regions; and coefficient 0.0447 (95% CI: 0.033–0.057), *P* = 1.96 × 10^−12^ for the patient-level mitotic density within the positive regions; coefficient 377.916 (95% CI: 297.677–458.155), *P* = 3.10 × 10^−19^, for the patient-level tumor nuclei area within the positive regions.

### Outcome analysis

Analysis of distant recurrence-free probabilities was conducted according to the Kaplan–Meier method and the two-sided log rank test with statistical significance accepted for *P* < 0.050. Cutoffs for the HECTOR risk groups were defined by taking the quantiles (25%, 50% and 75%) of the distribution of HECTOR risk scores in the training set only. In the training set, the first two groups (<25% and between 25% and 50%) did not show any major difference in prognosis and were therefore merged into one group named the HECTOR low-risk group. As a result, we defined the HECTOR low-risk group as cases with a risk score below the median risk score value of the training set, the HECTOR intermediate-risk group as those with a risk score between median and third quartile values of the training set and the HECTOR high-risk group as those with a risk score greater than the third quartile value of the training set. These same cutoff values were applied to the unseen internal, UMCG and LUMC external test sets, and the TCGA-UCEC and PORTEC-3.

To compare the DL model performance with well-established clinicopathological risk factors, we fitted CPH models on these clinicopathological risk factors in EC and calculated the corresponding C-index. First, we used risk factors that can be visually assigned on histological slides: the histological subtype, the grade and LVSI. Then we added the FIGO 2009 stage I–III variable. Finally, we included the molecular class of EC (*POLE*mut, MMRd, NSMP and p53abn). To maintain consistency within validation sets in the fivefold crossvalidation and the internal test sets, missing molecular class (115 out of 1,408 in crossvalidation and 38 out of 353 in the internal test set) was imputed using mean substitution.

To estimate HECTOR’s prognostic value as compared to the clinicopathological risk factors, we computed HRs using CPH with HECTOR continuous risk scores. For these analyses, we included all cases with a complete set of clinicopathological and molecular risk factors (*n* = 1,254). First, we corrected the HECTOR risk scores for all clinicopathological risk factors combined into one risk score in a multivariable analysis. To this end, a CPH model was first fitted on to these clinicopathological risk factors. Then, the derived risk scores, referred to as ‘clinical’, were calculated by taking the linear combination of the CPH coefficients and the variables. In the second analysis, we corrected HECTOR’s continuous risk scores for the histological subtype, the grade, LVSI, stage, the molecular class and, in addition, L1CAM and age as continuous data in a multivariable analysis.

The histological subtype categorical variable was processed as grade 3 EEC versus the reference group low-grade EEC and non-EEC versus the reference EEC. The reference group for molecular class was NSMP and stage I for the FIGO 2009 stage variable.

All statistical tests were two sided with statistical significance accepted for *P* values <0.050.

### Genomic and transcriptomic correlation analysis

To analyze the frequency of driver mutations by HECTOR risk groups, the genomic features were extracted from ref. ^70^ using MC3 MAF (mutation annotation format) data. The mutational status of the top 19 oncogenic drivers in EC was downloaded from the cBioPortal portal^65,66^ and annotated by OncoKB^71^. The statistical comparison of proportions with oncogenic mutations between HECTOR risk groups was performed using the two-sided *χ*^2^ tests for each individual gene with *P* < 0.050 accepted as significant. Exact *P* values and sample size are reported in Supplementary Table 12.

The association between the HECTOR continuous risk scores and each immune cell subset was performed using the log_2_(transformed proportion of the immune cell subset) as a fraction of the whole tumor, using the leukocyte fraction values. Linear regressions were performed with the HECTOR continuous risk scores as the independent variable. In addition, we tested the associations by correcting for the molecular class and TMB as additional independent variables. Two-sided *P* values <0.050 are accepted as significant. Regression coefficients and exact *P* values have been reported in Supplementary Table 13.

Messenger RNA sequencing (mRNA-seq) and clinical data from TCGA-UCEC were downloaded from firebrowse.org. Differentially expressed genes were assessed between HECTOR high-risk and HECTOR low-risk cases by DESeq2 (ref. ^72^) (v.1.40.1). Genes with a likelihood ratio test *P* value adjusted using a Benjamini–Hochberg false discovery rate (FDR) were accepted if <0.050 (Supplementary Table 15).

### Analysis of adjuvant chemotherapy effect

We predicted the HECTOR risk scores for the patients included in the PORTEC-3 (ref. ^3^) treatment arm who did receive concurrent and adjuvant chemotherapy (*n* = 225) and, thus, who had been previously left out from training and any test sets. The effect of the combination of adjuvant chemotherapy and external beam radiotherapy over external beam radiotherapy alone was analyzed by: (1) analyzing distant recurrence-free probabilities by treatment arm stratified by HECTOR risk group and measuring group-wise treatment effect with the Kaplan–Meier method and the two-sided log rank test and/or HR of treatment variable with the univariable Cox’s model; (2) calculating the statistical significance of the interaction term between the HECTOR continuous risk scores and the treatment binary variable; and (3) calculating the statistical significance of the interaction term between the HECTOR high-risk group and the treatment binary variable (corrected for HECTOR intermediate-risk group and using HECTOR low-risk group as a reference group). To measure the statistical significance of the interaction term defined as the HECTOR risk score (continuous or categorical) multiplied by the treatment binary variable, a multivariable Cox’s regression analysis was performed. Similar analyses were performed to test the interaction between serous histological subtype and the chemotherapy treatment binary variable (corrected for EEC and clear cell histological subtype), and the FIGO 2009 stage III (corrected for stages I–II) and p53abn (corrected for MMRd, NSMP as a reference group and *POLE*mut tumors removed to reach convergence).

All statistical tests were two sided with statistical significance accepted with *P* values <0.050.

### Software and packages

EsVIT and HECTOR were implemented with Pytorch (v.1.8.1 and v.1.10.0, respectively). IG was implemented with Captum Python package (v.0.6.0), metrics such as the C-index with scikit-survival Python package (v.0.17.2), CPH models and the Kaplan–Meier method with Lifelines Python package (v.0.27.1), *χ*^2^ tests with Scipy Python package (v.1.5.2), boxplot visualizations with altair Python package (v.4.2.0) and linear regression with statsmodels Python package (v.0.13.5). Differentially expressed genes were performed using DESeq2 (v.1.40.1)^72^ and R v.4.3.0 (2023-04-21 ucrt). Additional packages for image processing included Openslide Python package (v.1.1.2), OpenCV (v.4.3.0.36) and Pillow (v.7.2.0). Annotations were done with QuPath (v.0.4.1).

### Reporting summary

Further information on research design is available in the Nature Portfolio Reporting Summary linked to this article.

## Online content

Any methods, additional references, Nature Portfolio reporting summaries, source data, extended data, supplementary information, acknowledgements, peer review information; details of author contributions and competing interests; and statements of data and code availability are available at 10.1038/s41591-024-02993-w.

### Supplementary information

Supplementary Information Supplementary Figs. 1–31, Tables 1–14 and Notes.

Reporting Summary

Supplementary Table 15 Supplementary Table 15, as described in the manuscript, provided the exact *P* values of the analysis performed in Fig. 5c.

## Data Availability

The tumor material and datasets generated during or analyzed in the present study are not publicly available owing to restrictions by privacy laws. Data and tumor material from PORTEC-1, PORTEC-2, PORTEC-3, MST and the TransPORTEC study are held by the PORTEC study group and the international TransPORTEC consortium. Data and tumor material from the Danish cohort are held by the coauthor of this article, G.Ø. Data and tumor material from the UMCG cohort are held by the coauthors of this article, H.W.N. and M.d.B., and from the LUMC by the coauthors N.H. and T.B. Requests for sharing of all data and material should be addressed to the corresponding author within 15 years of the date of publication of this article and include a scientific proposal. Depending on the specific research proposal, the TransPORTEC consortium (PORTEC-3 and TransPORTEC study) or the PORTEC study group (PORTEC-1, PORTEC-2 and MST) or coauthors G.Ø., H.W.N. and M.d.B., or N.H. and T.B., will determine when, for how long, for which specific purposes and under which conditions the requested data can be made available, subject to ethical consent. Requests for data access will be processed within a 3-month timeframe. TCGA-UCEC images, mutational status and clinical data are publicly available via the cBioPortal^65,66^ for Cancer Genomics at https://www.cbioportal.org/study/clinicalData?id=ucec_tcga_pan_can_atlas_2018. The mRNA-seq data of the TCGA-UCEC were downloaded from http://firebrowse.org/?cohort=UCEC.

## Code availability

The code base is available at https://github.com/AIRMEC/HECTOR.
