# Author Correction: Prediction of recurrence risk in endometrial cancer with multimodal deep learning

**DOI:** 10.1038/s41591-024-03126-z

**Published:** 2024-07-01

**Authors:** Sarah Volinsky-Fremond, Nanda Horeweg, Sonali Andani, Jurriaan Barkey Wolf, Maxime W. Lafarge, Cor D. de Kroon, Gitte Ørtoft, Estrid Høgdall, Jouke Dijkstra, Jan J. Jobsen, Ludy C. H. W. Lutgens, Melanie E. Powell, Linda R. Mileshkin, Helen Mackay, Alexandra Leary, Dionyssios Katsaros, Hans W. Nijman, Stephanie M. de Boer, Remi A. Nout, Marco de Bruyn, David Church, Vincent T. H. B. M. Smit, Carien L. Creutzberg, Viktor H. Koelzer, Tjalling Bosse

**Affiliations:** 1 Department of Pathology, Leiden University Medical Center, Leiden, The Netherlands; 2 Department of Radiation Oncology, Leiden University Medical Center, Leiden, The Netherlands; 3 Department of Computer Science, ETH Zurich, Zurich, Switzerland; 4 Department of Pathology and Molecular Pathology, University Hospital, University of Zurich, Zurich, Switzerland; 5 Swiss Institute of Bioinformatics, Lausanne, Switzerland; 6 Department of Gynecology and Obstetrics, Leiden University Medical Center, Leiden, The Netherlands; 7 Department of Gynecology, Copenhagen University Hospital, Rigshospitalet, Copenhagen, Denmark; 8 Department of Pathology, Herlev University Hospital, Herlev, Denmark; 9 Department of Radiology, Leiden University Medical Center, Leiden, The Netherlands; 10 Department of Radiation Oncology, Medisch Spectrum Twente, Enschede, The Netherlands; 11 Maastricht Radiation Oncology, MAASTRO, Maastricht, The Netherlands; 12 Department of Clinical Oncology, Barts Health NHS Trust, London, UK; 13 Department of Medical Oncology, Peter MacCallum Cancer Center, Melbourne, Victoria Australia; 14 Department of Medical Oncology and Hematology, Odette Cancer Center Sunnybrook Health Sciences Center, Toronto, Ontario Canada; 15 Department Medical Oncology, Gustave Roussy Institute, Villejuif, France; 16 Department of Surgical Sciences, Gynecologic Oncology, Città della Salute and S Anna Hospital, University of Turin, Turin, Italy; 17 Department of Obstetrics and Gynecology, University Medical Center Groningen, University of Groningen, Groningen, The Netherlands; 18 Department of Radiotherapy, Erasmus MC Cancer Institute, University Medical Center Rotterdam, Rotterdam, The Netherlands; 19 Wellcome Centre for Human Genetics, University of Oxford, Oxford, UK; 20 Oxford NIHR Comprehensive Biomedical Research Centre, Oxford University Hospitals NHS Foundation Trust, Oxford, UK; 21 Institute of Medical Genetics and Pathology, University Hospital Basel, Basel, Switzerland

**Keywords:** Probabilistic data networks, Endometrial cancer, Machine learning, Prognostic markers, Pathology

Correction to: *Nature Medicine* 10.1038/s41591-024-02993-w, published online 24 May 2024

In the version of the article initially published, Viktor H. Koelzer and Tjalling Bosse were not named as equal contributors. This has now been corrected in the HTML and PDF versions of the article.

