# Supplementary material for: Prediction of recurrence risk in endometrial cancer with multimodal deep learning
Source: Nat Med. 2024 May 24;30(7):1962–73. doi: 10.1038/s41591-024-02993-w (PMC11271412; doi:10.1038/s41591-024-02993-w)
Supplement: Supplementary file 1 — Supplementary Figs. 1–31, Tables 1–14 and Notes. [file 41591_2024_2993_MOESM1_ESM.pdf]

nature medicine

Supplementary information

https://doi.org/10.1038/s41591-024-02993-w

Prediction of recurrence risk in endometrial cancer with multimodal deep learning

In the format provided by the authors and unedited

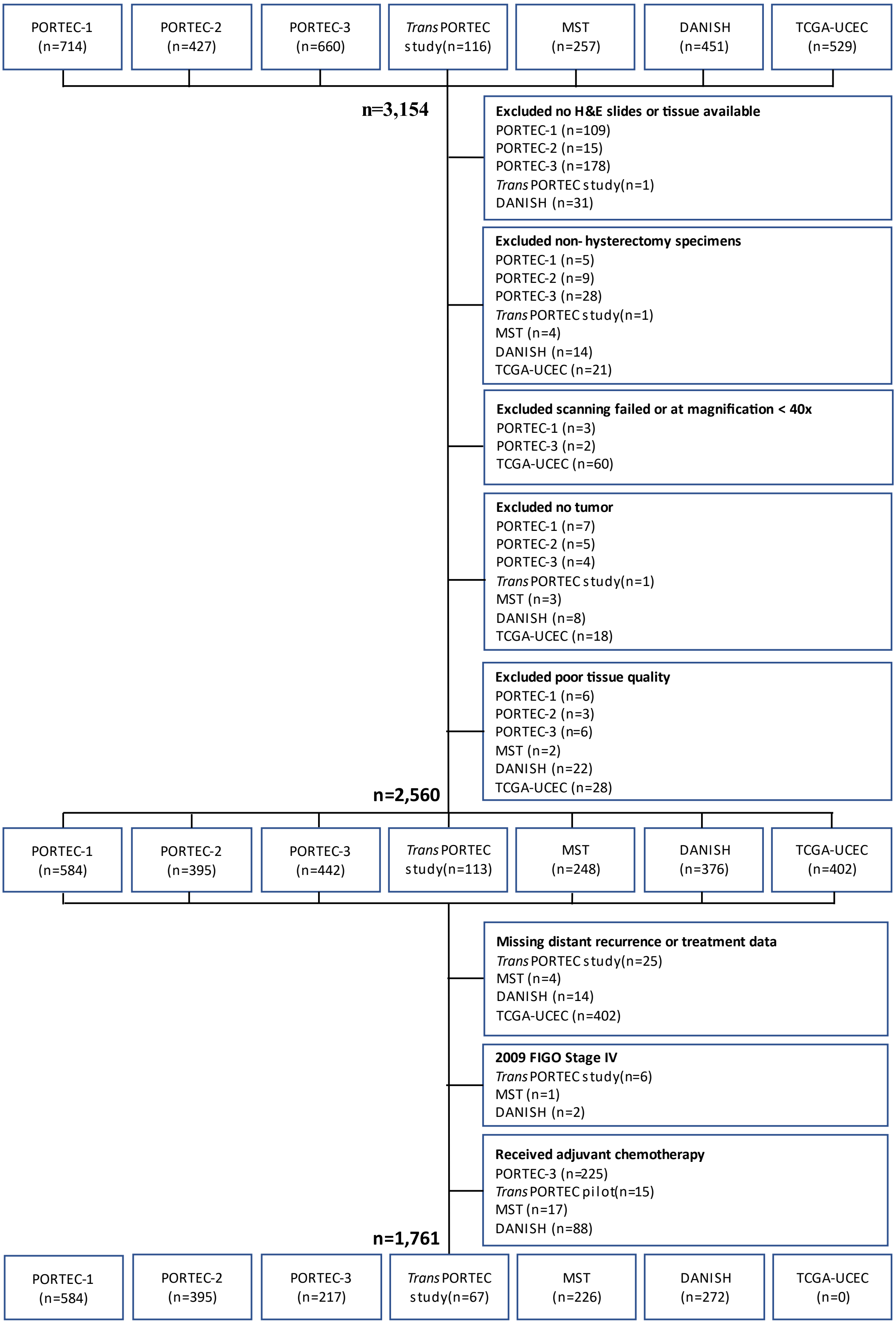

Figure 1: CONSORT flow diagram. The exclusion of cases with no distant recurrence outcomes, 2009 FIGO Stage IV, or received adjuvant chemotherapy was applied for supervised training of HECTOR. Poor tissue quality was defined as tumor tissue with extensive discohesive tumor cells due to poor fixation or very extensive necrosis, or due to very cutting artifacts extensively obliterating the tumoral area.

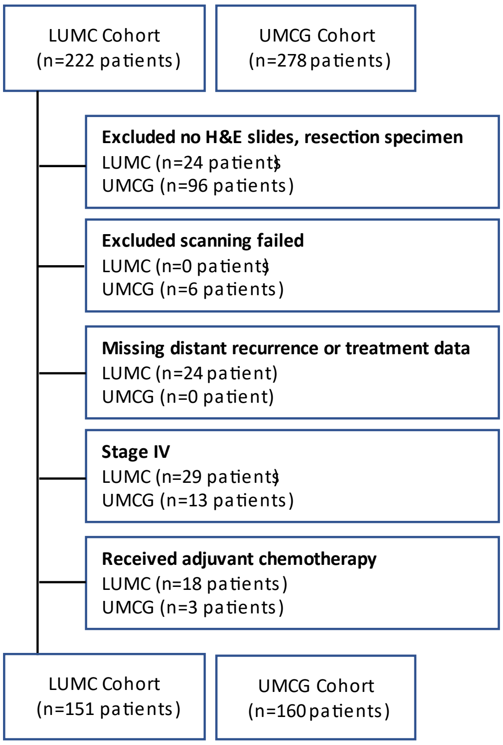

	Inclusion	LUMC Cohort	UMCG Cohort
**Number of patients (%)**		**222**	**278**
	yes	151 (68.0%)	160
	no	71 (32.0%)	118
**Follow-up: median**			
	yes	2.90	5.42
	no	2.62	4.24
**Age: median**			
	yes	66	66
	no	68	61
**Histotype (%)**			
Endometrioid grade 1-2	yes	88 (39.6%)	142 (51.1%)
	no	23 (10.4%)	14 (5.0%)
Endometrioid grade 3	yes	22 (9.9%)	7 (2.5%)
	no	10 (4.5%)	1 (0.4%)
Serous carcinoma	yes	17 (7.7%)	7 (2.5%)
	no	18 (8.1%)	0 (0%)
Clear cell carcinoma	yes	12 (5.4%)	0 (0%)
	no	4 (1.8%)	0 (0%)
Carcinosarcoma	yes	3 (1.4%)	0 (0%)
	no	9 (4.1%)	0 (0%)
Un-dedifferentiated	yes	3 (1.4%)	0 (0%)
	no	3 (1.4%)	0 (0%)
Other/Unknown	yes	6 (2.7%)	4 (1.4%)
	no	4 (1.8%)	103 (37.1%)
**LVSI (%)**			
Present	yes	14 (6.3%)	0 (0%)
	no	11 (5.0%)	0 (0%)
Focal or absent	yes	127 (57.2%)	0 (0%)
	no	46 (20.7%)	0 (0%)
Unknown	yes	10 (4.5%)	160 (57.6%)
	no	14 (6.3%)	118 (42.4%)
**2009 FIGO stage (%)**			
IA	yes	76 (34.2%)	112 (40.3%)*
	no	19 (8.6%)	65 (23.4%)*
IB	yes	42 (18.9%)	/
	no	7 (3.2%)	/
II	yes	14 (6.3%)	19 (6.8%)
	no	0 (0%)	13 (4.7%)
IIIA	yes	8 (3.6%)	29 (10.4%)*
	no	6 (2.7%)	27 (9.7%)*
IIIB	yes	1 (0.5%)	/
	no	2 (0.9%)	/
IIIC	yes	10 (4.5%)	/
	no	7 (3.2%)	/
IV	yes	0 (0%)	0 (0%)
	no	4 (1.8%)	13 (4.7%)
Unkown	yes	0 (0%)	0 (0%)
	no	26 (11.7%)	0 (0%)
**Adjuvant treatment (%)**			
None	yes	79 (35.6%)	/
	no	17 (7.7%)	/
VBT alone	yes	36 (16.2%)	/
	no	7 (3.2%)	/
EBRT alone	yes	25 (11.3%)	/
	no	6 (2.7%)	/
VBT + EBRT	yes	11 (5.0%)	160 (57.6%)*
	no	0 (0%)	115 (41.4%)*
Chemotherapy + EBRT	yes	0 (0%)	/
	no	15 (6.8%)	/
Chemotherapy alone	yes	0 (0%)	0 (0%)*
	no	25 (11.3%)	3 (1.1%)*
Unkown	yes	0 (0%)	/
	no	1 (0.5%)	/

Figure 2: Flow chart (left) and characteristics (right) of the two external test sets, the LUMC cohort and the UMCG cohort. VBT = vaginal brachytherapy; EBRT = external beam radiotherapy. (*) For the UMCG cohort, only (2009 FIGO) stage I, II, III was available with no further granularity in subclasses, for the adjuvant treatment only the binary variable whether patient received chemotherapy or not was available.

		PORTEC-1	PORTEC-2	PORTEC-3	*Trans* PORTEC study	MST	Danish
	**Inclusion**						
**Number of patients (%)**		**714**	**427**	**660**	**116**	**257**	**451**
	yes train	468 (65.5%)	295 (69.1%)	174 (26.4%)	54 (46.6%)	191 (74.3%)	226 (50.1%)
	yes test	116 (16.2%)	100 (23.4%)	43 (6.5%)	13 (11.2%)	35 (13.6%)	46 (10.2%)
	no	130 (18.2%)	32 (7.5%)	443 (67.1%)	49 (42.2%)	31 (12.1%)	179 (39.7%)
**Follow-up: median**							
	yes train	10.54	9.49	4.99	3.30	5.92	6.57
	yes test	10.77	10.03	5.23	3.02	3.34	6.33
	no	9.79	9.58	5.05	1.36	4.94	5.84
**Age: median**							
	yes train	67	70	61	65	70	70
	yes test	67	69	63	71	65	70
	no	64	69	63	69	66	68
**Histotype (%)**							
Endometrioid grade 1-2	yes train	389 (54.5%)	261 (61.1%)	73 (11.1%)	13 (11.2%)	77 (30.0%)	0 (0%)
	yes test	91 (12.7%)	85 (19.9%)	19 (2.9%)	1 (0.9%)	14 (5.4%)	0 (0%)
	no	121 (16.9%)	28 (6.6%)	165 (25.0%)	4 (3.4%)	10 (3.9%)	0 (0%)
Endometrioid grade 3	yes train	68 (9.5%)	28 (6.6%)	54 (8.2%)	39 (33.6%)	52 (20.2%)	111 (24.6%)
	yes test	19 (2.7%)	9 (2.1%)	8 (1.2%)	10 (8.6%)	11 (4.3%)	24 (5.3%)
	no	8 (1.1%)	4 (0.9%)	123 (18.6%)	19 (16.4%)	6 (2.3%)	57 (12.6%)
Serous carcinoma	yes train	7 (1.0%)	5 (1.2%)	20 (3.0%)	1 (0.9%)	20 (7.8%)	71 (15.7%)
	yes test	1 (0.1%)	6 (1.4%)	8 (1.2%)	2 (1.7%)	2 (0.8%)	14 (3.1%)
	no	1 (0.1%)	0 (0%)	77 (11.7%)	9 (7.8%)	3 (1.2%)	60 (13.3%)
Clear cell carcinoma	yes train	2 (0.3%)	0 (0%)	15 (2.3%)	1 (0.9%)	8 (3.1%)	18 (4.0%)
	yes test	2 (0.3%)	0 (0%)	7 (1.1%)	0 (0%)	2 (0.8%)	6 (1.3%)
	no	0 (0%)	0 (0%)	40 (6.1%)	17 (14.7%)	4 (1.6%)	12 (2.7%)
Carcinosarcoma	yes train	0 (0%)	0 (0%)	0 (0%)	0 (0%)	20 (7.8%)	8 (1.8%)
	yes test	0 (0%)	0 (0%)	0 (0%)	0 (0%)	2 (0.8%)	1 (0.2%)
	no	0 (0%)	0 (0%)	0 (0%)	0 (0%)	2 (0.8%)	4 (0.9%)
Un-dedifferentiated	yes train	0 (0%)	0 (0%)	2 (0.3%)	0 (0%)	7 (2.7%)	11 (2.4%)
	yes test	0 (0%)	0 (0%)	1 (0.2%)	0 (0%)	1 (0.4%)	1 (0.2%)
	no	0 (0%)	0 (0%)	11 (1,7%)	0 (0%)	1 (0.4%)	5 (1.1%)
Other	yes train	2 (0.3%)	1 (0.2%)	10 (1.5%)	0 (0%)	7 (2.7%	7 (1.6%)
	yes test	3 (0.4%)	0 (0%)	0 (0%)	0 (0%)	3 (1.2%)	0 (0%)
	no	0 (0%)	0 (0%)	27 (4.1%)	0 (0%)	5 (1.9%)	41 (9.1%)
**LVSI (%)**							
Present	yes train	20 (2.8%)	13 (3.0%)	112 (17.0%)	25 (21.6%)	40 (15.6%)	23 (5.1%)
	yes test	4 (0.6%)	6 (1.4%)	23 (3.5%)	6 (5.2%)	7 (2.7%)	1 (0.2%)
	no	2 (0.3%)	1 (0.2%)	254 (38.5%)	24 (20.7%)	6 (2.3%)	31 (6.9%)
Focal or absent	yes train	410 (57.4%)	269 (63.0%)	62 (9.4%)	28 (24.1%)	151 (58.8%)	202 (44.8%)
	yes test	101 (14.1%)	90 (21.1%)	20 (3.0%)	7 (6.0%)	27 (10.5%)	45 (10.0%)
	no	26 (3.6%)	14 (3.3%)	189 (28.6%)	5 (4.3%)	24 (9.3%)	107 (23.7%)
Unknown	yes train	38 (5.3%)	13 (3.0%)	0 (0%)	1 (0.9%)	0 (0%)	1 (0.2%)
	yes test	11 (1.5%)	4 (0.9%)	0 (0%)	0 (0%)	1 (0.4%)	0 (0%)
	no	102 (14.3%)	17 (4.0%)	0 (0%)	20 (17.2%)	1 (0.4%)	41 (9.1%)

Table 1: Characteristics (such as median follow-up, age, histoype and LVSI) of the cohorts used in this study stratified by whether patients were included in the supervised training, supervised testing or excluded. Exclusion criteria included lack of material (H&E slide), missing distant recurrence data, 2009 FIGO stage IV and patients who received adjuvant chemotherapy. LVSI = lymphovascular space invasion.

	Inclusion	PORTEC-1	PORTEC-2	PORTEC-3	*Trans* PORTEC study	MST	Danish
**2009 FIGO stage (%)**							
IA	yes train	190 (26.6%)	47 (11.0%)	21 (3.2%)	3(2.6%)	20 (7.8%)	126 (27.9%)
	yes test	47 (6.6%)	16 (3.7%)	5 (0.8%)	1 (0.9%)	2 (0.8%)	24 (5.3%)
	no	57 (8.0%)	8 (1.9%)	52 (7.9%)	7 (6.0%)	3 (1.2%)	24 (5.3%)
IB	yes train	278 (38.9%)	244 (57.1%)	32 (4.8%)	20 (17.2%)	57 (22.2%)	66 (14.6%)
	yes test	69 (9.7%)	83 (19.4%)	6 (0.9%)	4 (3.4%)	9 (3.5%)	12 (2.7%)
	no	73 (10.2%)	24 (5.6%)	79 (12.0%)	7 (6.0%)	2 (0.8%)	18 (4.0%)
II	yes train	0 (0%)	2 (0.5%)	50 (7.6%)	13 (11.2%)	62 (24.1%)	17 (3.8%)
	yes test	0 (0%)	0 (0%)	10 (1.5%)	4 (3.4%)	7 (2.7%)	3 (0.7%)
	no	0 (0%)	0 (0%)	110 (16.7%)	4 (3.4%)	10 (3.9%)	9 (2.0%)
IIIA	yes train	0 (0%)	2 (0.5%)	18 (2.7%)	9 (7.8%)	33 (12.8%)	1 (0.2%)
	yes test	0 (0%)	0 (0%)	6 (0.9%)	2 (1.7%)	11 (4.3%)	2 (0.4%)
	no	0 (0%)	0 (0%)	59 (8.9%)	7 (6.0%)	6 (2.3%)	3 (0.7%)
IIIB	yes train	0 (0%)	0 (0%)	16 (2.4%)	1 (0.9%)	8 (3.1%)	5 (1.1%)
	yes test	0 (0%)	1 (0.2%)	1 (0.2%)	0 (0%)	4 (1.6%)	3 (0.7%)
	no	0 (0%)	0 (0%)	25 (3.8%)	3 (2.6%)	6 (2.3%)	6 (1.3%)
IIIC	yes train	0 (0%)	0 (0%)	37 (5.6%	8 (6.9%)	11 (4.3%)	11 (2.4%)
	yes test	0 (0%)	0 (0%)	15 (2.3%)	2 (1.7%)	2 (0.8%)	2 (0.4%)
	no	0 (0%)	0 (0%)	118 (17.9%)	9 (7.8%)	2 (0.8%)	48 (10.6%)
IV	yes train	0 (0%)	0 (0%)	0 (0%)	0 (0%)	0 (0%)	0 (0%)
	yes test	0 (0%)	0 (0%)	0 (0%)	0 (0%)	0 (0%)	0 (0%)
	no	0 (0%)	0 (0%)	0 (0%)	11 (9.5%)	2 (0.8%)	32 (7.1%)
Unkown	yes train	0 (0%)	0 (0%)	0 (0%)	0 (0%)	0 (0%)	0 (0%)
	yes test	0 (0%)	0 (0%)	0 (0%)	0 (0%)	0 (0%)	0 (0%)
	no	0 (0%)	0 (0%)	0 (0%)	1 (0.9%)	0 (0%)	0 (0%)
**Molecular class (%)**							
POLE mut	yes train	31 (4.3%)	17 (4.0%)	21 (3.2%)	12 (10.3%)	12 (4.7%)	29 (6.4%)
	yes test	9 (1.3%)	7 (1.6%)	6 (0.9%)	2 (1.7%)	2 (0.8%)	2 (0.4%)
	no	2 (0.3%)	0 (0%)	24 (3.6%)	2 (1.7%)	2 (0.8%)	7 (1.6%)
MMRd	yes train	111 (15.5%)	85 (19.9%)	59 (8.9%)	10 (8.6%	58 (22.6%)	61 (13.5%)
	yes test	22 (3.1%)	20 (4.7%)	11 (1.7%)	1 (0.9%)	7 (2.7%)	14 (3.1%)
	no	4 (0.6%)	5 (1.2%)	69 (10.5%)	9 (7.8%)	6 (2.3%)	35 (7.8%)
NSMP	yes train	209 (29.3%)	162 (37.9%)	46 (7.0%)	21 (18.1%)	66 (25.7%)	36 (8.0%)
	yes test	51 (7.1%)	64 (15.0%)	11 (1.7%)	7 (6.0%)	16 (6.2%)	10 (2.2%)
	no	5 (0.7%)	6 (1.4%)	65 (9.8%)	15 (12.9%)	8 (3.1%)	28 (6.2%)
p53abn	yes train	30 (4.2%)	24 (5.6%)	32 (4.8%)	10 (8.6%)	51 (19.8%)	100 (22.2%)
	yes test	9 (1.3%)	4 (0.9%)	8 (1.2%)	3 (2.6%)	9 (3.5%)	20 (4.4%)
	no	1 (0.1%)	2 (0.5%)	59 (8.9%)	21 (18.1%)	9 (3.5%)	70 (15.5%)
Unkown	yes train	87 (12.2%)	7 (1.6%)	16 (2.4%)	1 (0.9%)	4 (1.6%)	0 (0%)
	yes test	25 (3.5%)	5 (1.2%)	7 (1.1%)	0 (0%)	1 (0.4%)	0 (0%)
	no	118 (16.5%)	19 (4.4%)	226 (34.2%)	2 (1.7%)	6 (2.3%)	39 (8.6%)
**Adjuvant treatment (%)**							
None	yes train	241 (33.8%)	1 (0.2%)	0 (0%)	3 (2.6%)	0 (0%)	198 (43.9%)
	yes test	63 (8.8%)	0 (0%)	0 (0%)	1 (0.9%)	0 (0%)	43 (9.5%)
	no	65 (9.1%)	2 (0.5%)	0 (0%)	6 (5.2%)	0 (0%)	30 (6.7%)
VBT alone	yes train	0 (0%)	148 (34.7%)	0 (0%)	1 (0.9%)	17 (6.6%)	0 (0%)
	yes test	0 (0%)	50 (11.7%)	0 (0%)	0 (0%)	2 (0.8%)	0 (0%)
	no	0 (0%)	17 (4.0%)	0 (0%)	0 (0%)	3 (1.2%)	0 (0%)
EBRT alone	yes train	227 (31.8%)	146 (34.2%)	174 (26.4%)	42 (36.2%)	174 (67.7%)	28 (6.2%)
	yes test	53 (7.4%)	50 (11.7%)	43 (6.5%)	8 (6.9%)	33 (12.8%)	3 (0.7%)
	no	65 (9.1%)	13 (3.0%)	116 (17.6%)	2 (1.7%)	7 (2.7%)	4 (0.9%)
EBRT + VBT	yes train	0 (0%)	0 (0%)	0 (0%)	8 (6.9%)	0 (0%)	0 (0%)
	yes test	0 (0%)	0 (0%)	0 (0%)	4 (3.4%)	0 (0%)	0 (0%)
	no	0 (0%)	0 (0%)	0 (0%)	1 (0.9%)	0 (0%)	0 (0%)
Chemotherapy + EBRT	yes train	0 (0%)	0 (0%)	0 (0%)	0 (0%)	0 (0%)	0 (0%)
	yes test	0 (0%)	0 (0%)	0 (0%)	0 (0%)	0 (0%)	0 (0%)
	no	0 (0%)	0 (0%)	327 (49.5%)	11 (9.5%)	15 (5.8%)	8 (1.8%)
chemotherapy alone	yes train	0 (0%)	0 (0%)	0 (0%)	0 (0%)	0 (0%)	0 (0%)
	yes test	0 (0%)	0 (0%)	0 (0%)	0 (0%)	0 (0%)	0 (0%)
	no	0 (0%)	0 (0%)	0 (0%)	4 (3.4%)	0 (0%)	98 (21.7%)
Unkown	yes train	0 (0%)	0 (0%)	0 (0%)	0 (0%)	0 (0%)	0 (0%)
	yes test	0 (0%)	0 (0%)	0 (0%)	0 (0%)	0 (0%)	0 (0%)
	no	0 (0%)	0 (0%)	0 (0%)	25 (21.6%)	6 (2.3%)	39 (8.6%)

Table 2: Characteristics (such as 2009 FIGO stage, Molecular class, Adjuvant treatment) of the cohorts used in this study stratified by whether patients were included in the supervised training, supervised testing or excluded. Exclusion criteria included lack of material (H&E slide), missing distant recurrence

data, 2009 FIGO stage IV and patients who received adjuvant chemotherapy. *POLE*mut = *POLE* mutant; MMRd = mismatch repair deficient; NSMP = No specific molecular profile; p53abn = p53 abnormal; VBT = vaginal brachytherapy; EBRT = external beam radiotherapy.

Self-supervised features	C-index⇧	Cumulative AUC⇧	IBS⇩
Modified EsVIT (8 blocks)	0.770±0.0193	0.823±0.0353	0.114±0.0118
Modified EsVIT (4 blocks)	0.742±0.0294	0.777±0.0503	0.105±0.0056
CtransPath	0.728±0.0332	0.765±0.0650	0.100±0.0021
Unimodal model (H&E only)			
AttentionMIL + merged EsVIT features	0.775±0.0312	0.810±0.0257	0.124±0.0057
AttentionMIL + EsVIT features	0.770±0.0193	0.823±0.0353	0.114±0.0118
GAT + merged EsVIT features	0.766±0.0109	0.804±0.0441	0.0986±0.0069
Transformer + merged EsVIT features	0.762±0.0201	0.801±0.0611	0.109±0.0101
Multimodal model<br>(in addition to merged EsVIT features)			
AttentionMIL (unimodal baseline)	0.775±0.0312	0.810±0.0257	0.124±0.0057
AttentionMIL + Transfer learning from im4MEC backbone to the prognostic task	0.764±0.026	0.815±0.0427	0.100±0.006
AttentionMIL + Multitask learning with two objective heads and weighted loss (prognostic and im4MEC 4 classes)	0.772±0.0252	0.816±0.0351	0.106±0.0105
AttentionMIL + fusion with image embeddings produced by im4MEC	0.764±0.0151	0.806±0.0371	0.111±0.0102
AttentionMIL + fusion with Embedding layer for im4MEC classes	0.779±0201	0.820±0.0354	0.110±0.0281
AttentionMIL + fusion with attention-gated Embedding layer for im4MEC classes	0.782±0.0264	0.824±0.0402	0.113±0.0094
AttentionMIL + fusion with categorical 2009 FIGO stage IA-IB-II-IIIA-IIIB-IIIC	0.767±0.0199	0.822±0.0367	0.104±0.0077
AttentionMIL + fusion with categorical 2009 FIGO stage I-II-III	0.772±0.0160	0.816±0.0245	0.101±0.0081
AttentionMIL + fusion with Embedding layer for 2009 FIGO stage I-II-III	0.786±0.0081	0.841±0.0424	0.106±0.0243
AttentionMIL + fusion with attention-gated Embedding layer for 2009 FIGO stage I-II-III	0.790±0.0089	0.830±0.0320	0.102±0.0065
**AttentionMIL + 2-sided attention-gated Embedding layer for im4MEC classes and categorical 2009 FIGO stage I-II-III (HECTOR)**	**0.795±0.0307**	**0.844±0.0290**	**0.097±0.0122**

Table 3: Ablation studies on five-fold cross-validation (*n* = 1,408 patients). (Top) Comparison of EsVIT[1] (a state-of-the-art transformer-based model trained with self-supervised learning, re-trained on endometrial cancer-specific patches, where patch-level embeddings are extracted from either the last four or height transformer blocks) and CtransPath[2] (trained on histology images). Performance was measured on the downstream supervised task using unimodal (H&E only) AttentionMIL[3], that is the prediction of distant recurrence-free probabilities. Modified EsVIT refers to the architecture described in Table 3. (Middle) Comparison of state-of-the-art supervised deep learning architectures for distant recurrence-free probabilities prediction using only H&E WSIs as input and EsVIT features from the last height blocks. All models were trained with the negative log likelihood loss[4] and a batch size of 1 for fair comparison. For GAT[5], we experimented a radius of 16 and 32 patches. Merged features extracted from EsVIT were obtained by averaging patches spatially and semantically merging using L2 norm of

three patches and a cosine similarity above 0.8. Transformers do not include patch positional encoding[6]. (Bottom) Ablation studies with multimodal deep learning, that is when including the anatomic stage as input and the image-based molecular class as predicted by im4MEC[7] from the H&E WSIs. Since the image-based molecular classes can be predicted from the H&E WSIs, experiments first covered transfer learning in which the same AttentionMIL backbone was pre-trained towards the 4-class prediction of the molecular classes then fine-tuned towards the prognostic task (with more of less fully connected layers following the backbone), and multitask learning where two objective heads (one classification with 4 classes, one prognostic) along with a weighted loss (weighted loss of the negative log likelihood loss for the survival task and cross-entropy for the classification task with weighted factor as one hyperparameter) were implemented. Second, we experimented fusing the WSI-level embedding using either the WSI-level embeddings produced by im4MEC or the categorical image-based molecular class as predicted by im4MEC[7]. For the anatomic stage, we used the 2009 FIGO stage classification with two taxonomies[8]. Mean and standard deviation across validation folds of C-index[9], cumulative AUC[10], IBS[11] are reported. Final hyperparameter selection was performed based on the highest mean C-index. HECTOR, which returned the highest mean C-index, was selected for downstream analysis. C-index=concordance index; AUC = area under the receiver operating characteristic curve; IBS = integrated Brier score; GAT = graph attention networks; AttentionMIL = attention multiple instance learning.

Modified Swin, W=14, S=256	Stage 1	Stage 2	Stage 3	Stage 4
Merging rate<br>Feature map	8x<br>32 x 32	16x<br>16 x 16	32x<br>8 x 8	64x<br>4 x 4
Architecture	$\begin{bmatrix} \text{window size: } 14 \times 14 \\ 96\text{-}d\ (3\ heads) \end{bmatrix} \times 2$	$\begin{bmatrix} \text{window size: } 14 \times 14 \\ 192\text{-}d\ (6\ heads) \end{bmatrix} \times 2$	$\begin{bmatrix} \text{window size: } 14 \times 14 \\ 384\text{-}d\ (12\ heads) \end{bmatrix} \times 4$	$\begin{bmatrix} \text{window size: } 14 \times 14 \\ 768\text{-}d\ (24\ heads) \end{bmatrix} \times 2$

Table 4: Architecture of the modified four-stage EsVIT used[1]. W = window size; S = Size of input image in pixels.

	Architecture	Training parameters
WSI preprocessing	- Otsu thresholding<br>- 180µm non-overlapping patching<br>- 256x256 pixels	
SSL	- Modified EsVIT (*see Table 5*)<br>- View and region-level prediction DINO heads<br>- Window of 14<br>- Output dimension of 65,536<br>- Features extracted at 8 blocks	- batch size 128<br>- no weight normalization<br>- frozen layers at first epoch<br>- momentum teacher 0.9996<br>- teacher temperature of 0.04; 0.05 at epoch 49<br>- AdamW and default parameters<br>- linear warm up for ten epochs followed by cosine scheduler to $1x10^{-6}$<br>- weight decay (cosine scheduler from 0.04 to 0.4)<br>- data augmentation as in the original publication[1]<br>- trained on 3 Nvidia RTX 8000 GPUs.
HECTOR	- H&E WSI with merged EsVIT features (3 nearby patches and 0.8 cosine similarity)<br>- 3 fully connected layers (down to 512) applied onto patch-level features<br>- AttentionMIL with latent features reduced to 256<br>- im4MEC frozen<br>- Tumor stage as 3-class category (I,II,III)<br>- Embedding layers for im4MEC and tumor stage (size 16 followed by Elu activation function and one fully connected layer of size 8)<br>- 2-sided attention-gates with bilinear product[12]<br>- 1 appended to the 3 attention-weighted embeddings<br>- fused using the Kronecker product<br>- final multimodal embedding reduced with two fully connected layers of size 256 and 128<br>- All fully connected layers followed by a dropout of 0.25 and a ReLU	- 24 epochs<br>- initial learning rate of $3x10^{-5}$ decayed by a factor of 10 at epoch 2, 5, and 15.<br>- Adam optimizer with default parameters<br>- weight decay of $1x10^{-5}$<br>- trained on 1 Nvidia RTX 6000 GPU.

Table 5: Summary of architectural components and hyperparameters of HECTOR.

Input required	Model	C-index⇧	Cumulative AUC⇧	IBS⇩
H&E tumor slide	Unimodal DL	0.775±0.0312	0.810±0.0257	0.124±0.0057
	Two-arm DL	0.782±0.0264	0.824±0.0402	0.113±0.0094
	CPH	0.676±0.0601	0.721±0.0742	0.129±0.1023
H&E tumor slide + 2009 FIGO stage I-III	HECTOR	**0.795±0.0307**	**0.844±0.0290**	**0.097±0.0122**
	CPH	0.723±0.0449	0.762±0.0539	0.128±0.0443
H&E tumor slide + 2009 FIGO stage I-III + true molecular class	CPH	0.736±0.0328	0.809±0.0472	0.128±0.0029

Table 6: Comparison of unimodal and multimodal deep learning models and baseline Cox proportional hazard (CPH) for distant recurrence-free probabilities prediction in the five-fold cross validation (n = 1,408 patients). We report the cumulative AUC[10] and IBS[11]. H&E-based variables used in CPH are histologic subtype, grade, and lymphovascular space invasion. 2009 FIGO I-III classification was used[8]. Unlike the C-index and Cumulative AUC, a lower IBS value is better. H&E = Hematoxylin and eosin; C-index=concordance index; cumulative AUC = cumulative area under the receiver operating characteristic curve; IBS= integrated brier score.

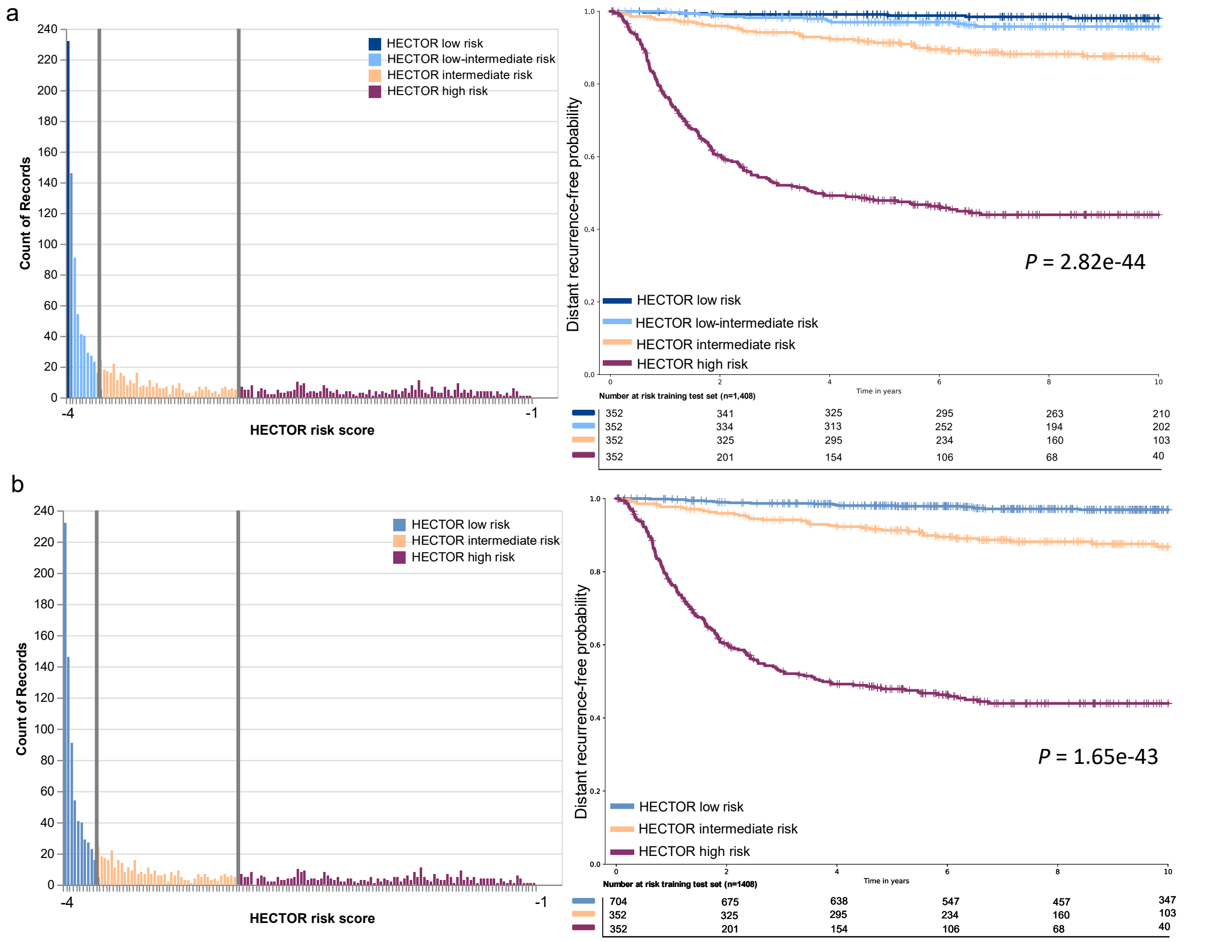

Figure 3: Categorization of the HECTOR continuous risk scores based on the quartiles of the distribution in the training set (*n* = 1,408 patients) and the corresponding 10-year distant recurrence-free probabilities in the training set. a, The quartiles are used to create four groups low, low-intermediate, intermediate and high risk (left). The corresponding distant recurrence-free

probabilities by Kaplan Meier analysis and the log-rank test *P*-value (right). b, Patients from low and low-intermediate were combined into the HECTOR low risk group (left). The corresponding distant recurrence-free probabilities by Kaplan Meier analysis and *P*-value of the log-rank test (right).

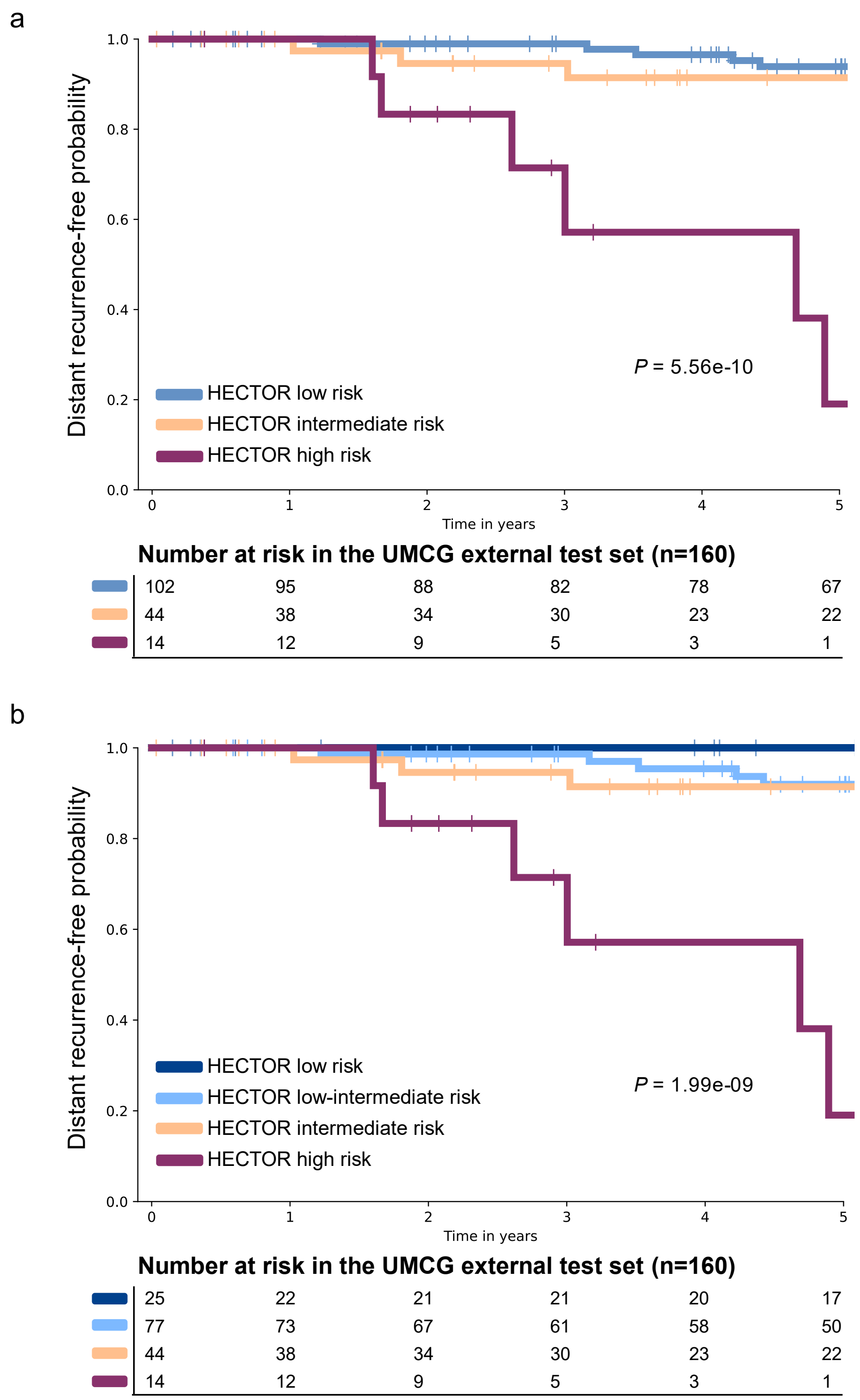

Figure 4: Analysis of the five-year distant recurrence-free probabilities in the UMCG external test set (n = 160). a, Stratified by HECTOR low, intermediate, high risk groups according to the quartiles and the first two combined in one low risk group from the training set. Kaplan Meir method was used and the *P*-value of the log-rank test displayed. b, Without combining the two first quartiles. Kaplan Meir method was used and the *P*-value of the log-rank test displayed.

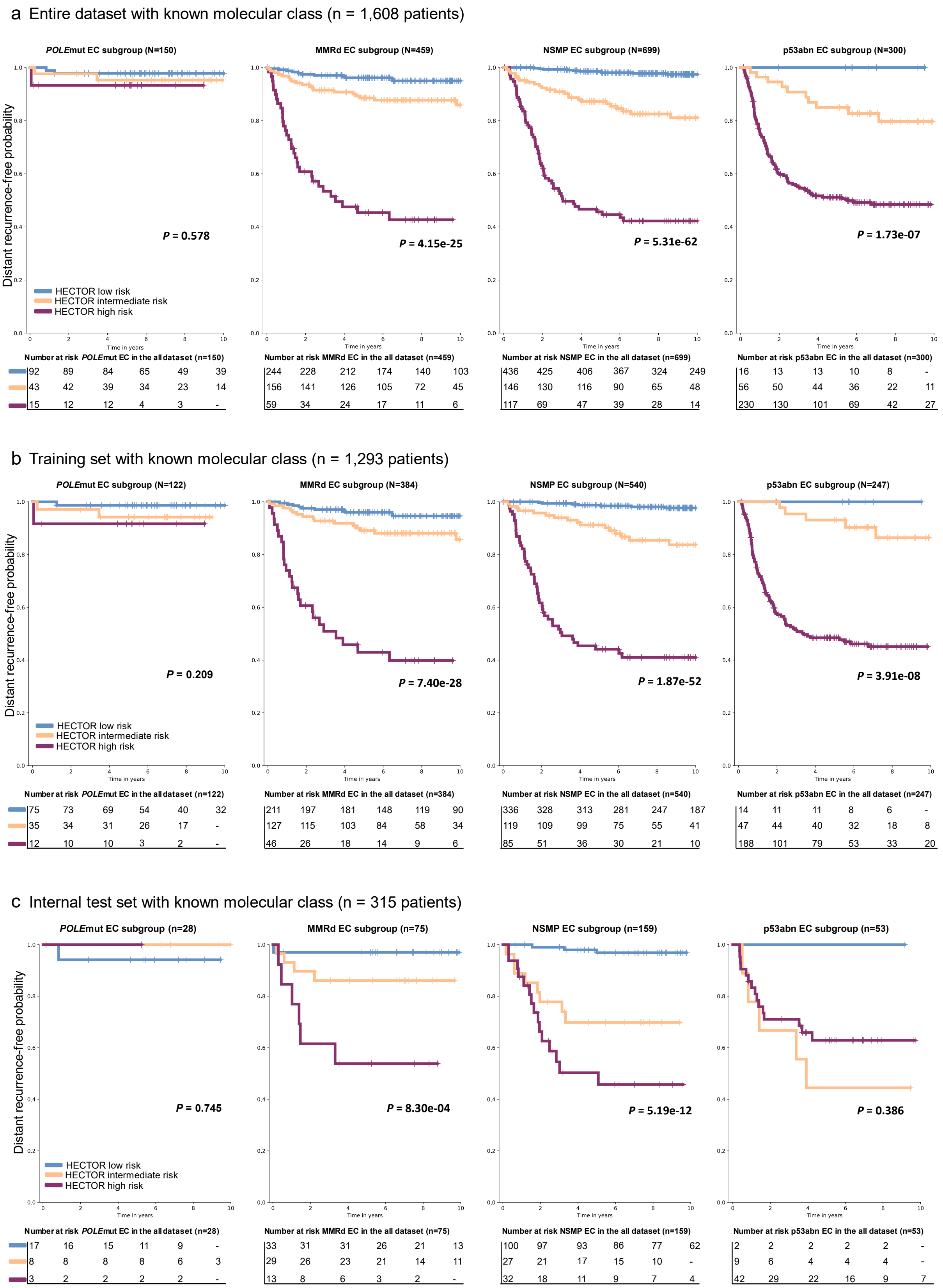

Figure 5: Analysis of the distant recurrence-free probability analysis using Kaplan-Meier method within each molecular class of endometrial cancer stratified by HECTOR risk groups. For each the *P*-value of the log-rank test is reported. MMRd= mismatch repair deficient; p53abn = p53 abnormal; NSMP = no specific molecular profile.

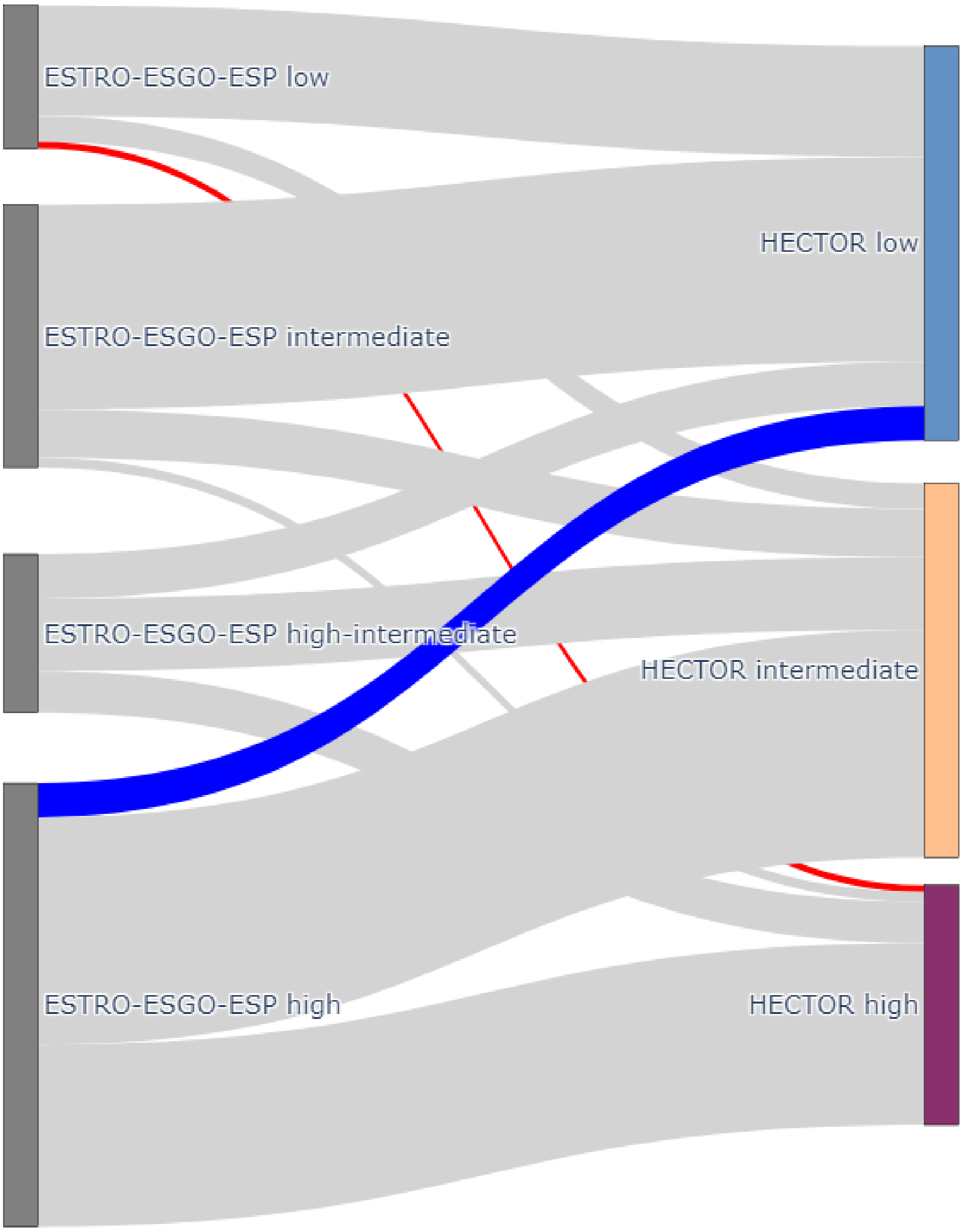

Figure 6: Shift between the risk assessment of the ESGO-ESTRO-ESP 2021 guidelines[13] and HECTOR risk group in the entire dataset (*n* = 1,608 patients) with all known prognostic factors (such as LVSI and molecular class).

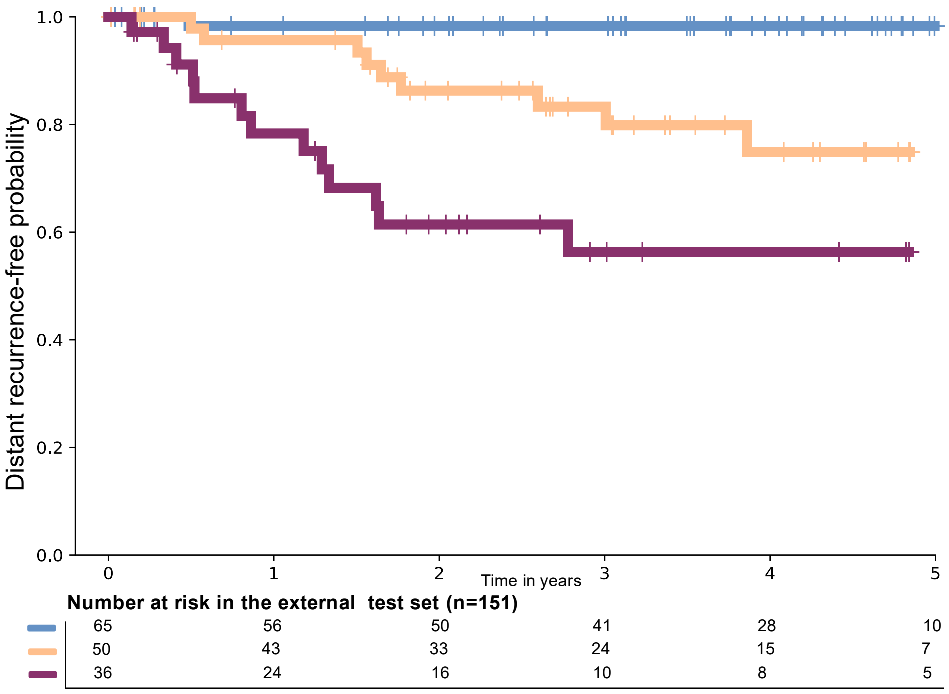

Figure 7: Five-year distant recurrence-free probabilities in the LUMC external test set (n = 151 patients) by post-aggregation with mean, i.e. taking the mean of the predicted HECTOR risk scores obtained for all the WSIs of each patient. Analysis was performed using Kaplan Meir method.

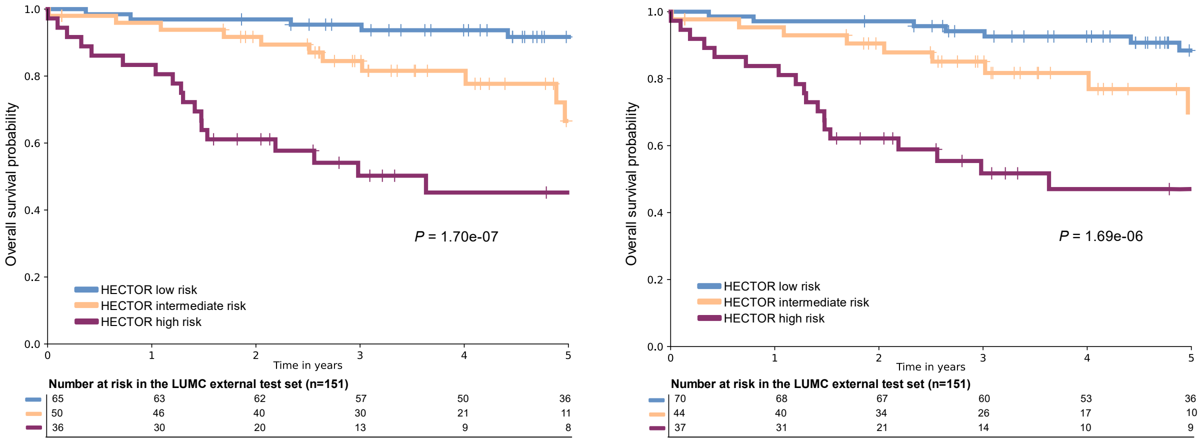

Figure 8: Five-year overall survival probabilities in the LUMC external test set (n = 151 patients) by post-aggregation with mean (left), i.e. taking the mean of the predicted HECTOR risk scores obtained for all the WSIs of each patient., and by median (right). Analysis was performed using the Kaplan Meier method and log-rank test P-values are displayed.

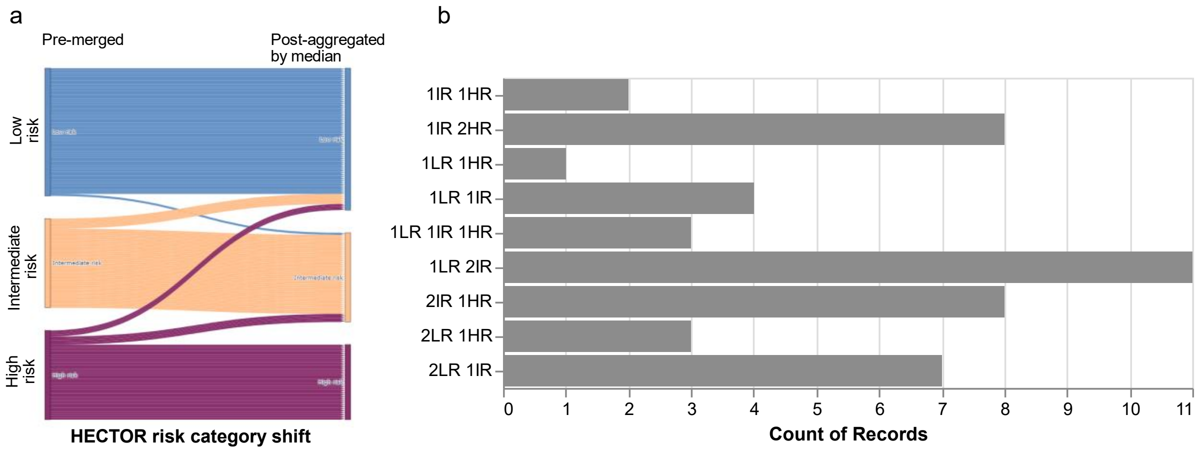

Figure 9: a, Sankey plot showing patient-level shifts between a scenario where the whole slide images are pre-merged into one bag of features (left) yielding a C-index of 0.805 and alternatively when they were post-aggregated by median (right) yielding a c-index of 0.816. b, Number of cases with more than one WSI with a different predicted risk category from HECTOR. LR = HECTOR low risk; IR = HECTOR intermediate risk; HR = HECTOR high risk.

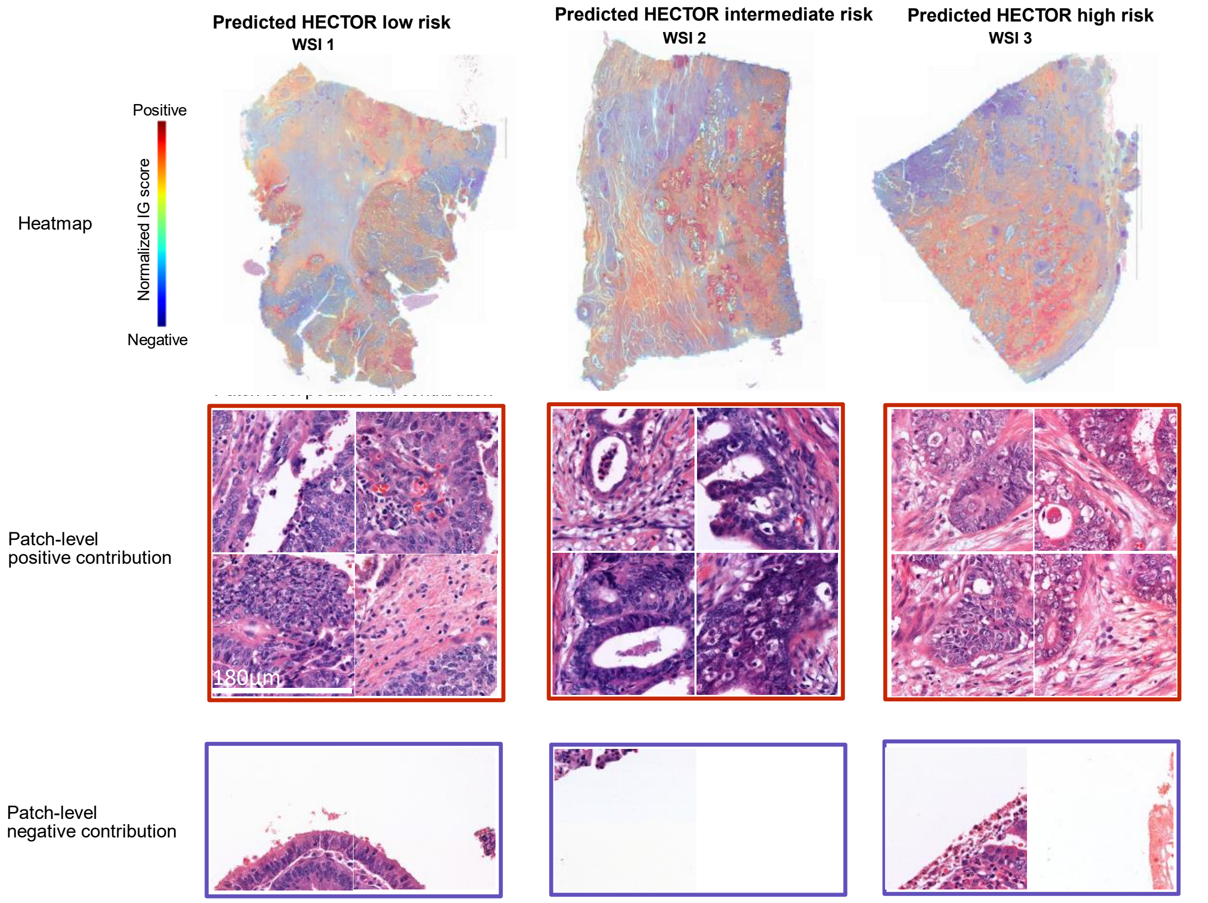

	WSI 1	WSI 2	WSI 3
Endpoint	Censored		
Histological subytpe	Endometrioid		
FIGO stage	III		
im4MEC class	imNSMP	imNSMP	imNSMP
HECTOR risk group	low risk	intermediate risk	high risk

Figure 10: First case out of three of the LUMC external test set (*n* = 151) with three WSIs respectively HECTOR low, intermediate and high risk. Contribution of each tissue region to the prediction was quantified with the Integrated Gradient method[14]. Each square patch is 180µm wide. Im4MEC class is the image-based molecular class as predicted by im4MEC[7]. WSI = whole slide image; im = image-based; NSMP = no specific molecular profile.

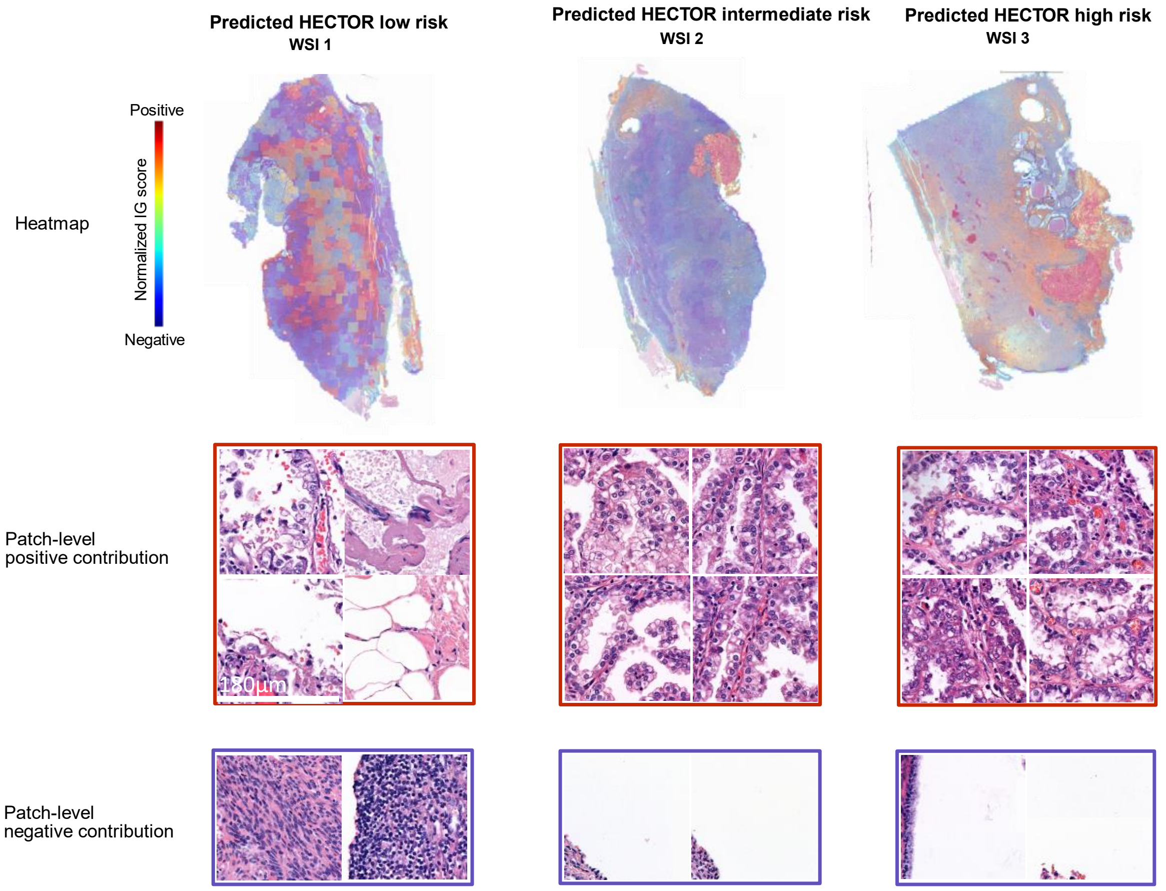

	WSI 1	WSI 2	WSI 3
Endpoint	Censored		
Histological subytpe	Clear Cell		
FIGO stage	II		
im4MEC class	im*POLE*mut	imp53abn	imp53abn
HECTOR risk group	low risk	intermediate risk	high risk

Figure 11: Second case out of three of the LUMC external test set (*n* = 151) with three WSIs predicted respectively HECTOR low, intermediate and high risk. Contribution of each tissue region to the prediction was quantified with the Integrated Gradient method[14]. Each square patch is 180µm wide. Im4MEC class is the image-based molecular class as predicted by im4MEC[7]. WSI = whole slide image; im = image-based; *POLE*mut = *POLE* mutated; p53abn = p53 abnormal.

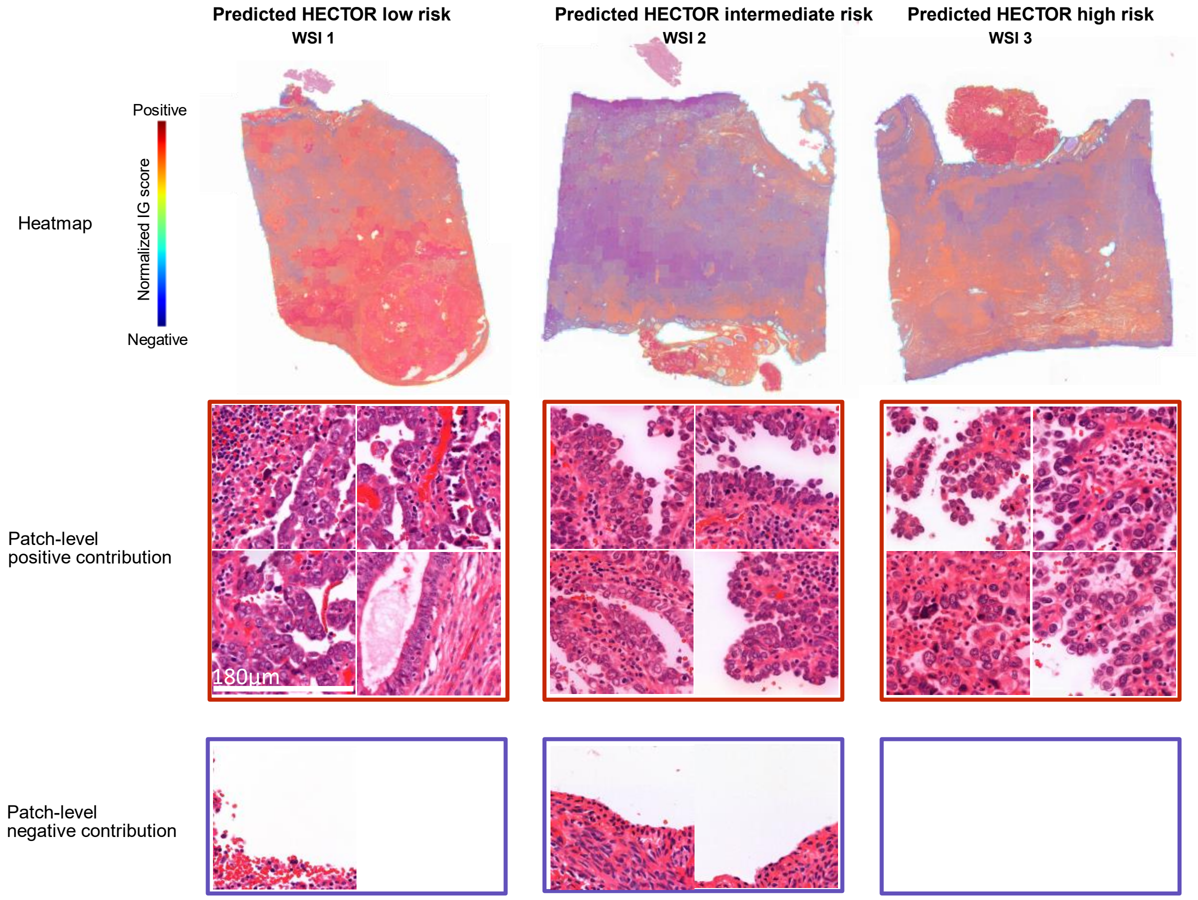

	WSI 1	WSI 2	WSI 3
Endpoint	Censored		
Histological subytpe	Clear Cell		
FIGO stage	I		
im4MEC class	im*POLE*mut	im*POLE*mut	imp53abn
HECTOR risk group	low risk	intermediate risk	high risk

Figure 12: Third case out of three of the LUMC external test set (*n* = 151) with three WSIs predicted respectively HECTOR low, intermediate and high risk. Contribution of each tissue region to the prediction was quantified with the Integrated Gradient method[14]. Each square patch is 180µm wide. Im4MEC class is the image-based molecular class as predicted by im4MEC[7]. WSI = whole slide image; im = image-based; *POLE*mut = *POLE* mutated; p53abn = p53 abnormal.

Notes:

Here in Supplementary Fig. 9a, two experiments performed in the LUMC external test set (*n* = 151 patients; *n* = 121 with three WSIs; *n* = 21 with two and *n* = 9 with one) are shown. The first one consisted of predicting the risk score separately for each WSI of each patient followed by taking the median of these scores (referred to as 'post-aggregation by median'). The second experiment consisted of merging all WSIs of each patient into one feature bag per patient, resulting in one risk score per patient (referred to as 'pre-merged'). These two aggregation methods resulted in different risk group predictions for only three cases which moved from HECTOR high risk using the pre-merged method to HECTOR low risk in the post-aggregated median method (since in the latter, two WSIs were predicted as HECTOR low risk and one WSI predicted as HECTOR high risk). This might indicate that, when WSIs are pre-merged and considered as a single input image, the visual features of the WSI predicted as HECTOR high risk may overrule the information present in the other two WSIs.

In Supplementary Fig. 9b, we showed that a number of patients with multiple WSIs had different HECTOR predictions for each WSI. Among the 142 cases with more than one WSI, 85 cases (59.9%) had consistent HECTOR risk group predictions across the WSIs. Only four cases (2.8%) had at least one WSI predicted as HECTOR low risk while another was predicted as HECTOR high risk. Only three cases (2.1%) each with three WSIs had a different predicted HECTOR risk group for each WSI. Upon review of these three cases, two cases showed the same pattern: one WSI was predicted as HECTOR low risk combined with im*POLE*mut, one WSI was predicted as HECTOR high risk and imp53abn which is in line with the respective negative and positive contributions to the risk score of the im*POLE*mut and imp53abn image-based molecular class. These three cases also showed heterogeneous tumor volumes and tumor features, including one case with the WSI predicted as HECTOR low risk presenting smooth luminal borders when the destructive component of the remaining two WSIs was more substantial (Supplementary Figs. 10-12).

Variable	Coefficient [95% CI]	*P* value
Endometrioid (EEC)	-1.124 [-1.337;-0.910]	1.87e-21
Serous (SEC)	1.025 [0.722;1.328]	1.50e-10
Clear cell (CCC)	0.862 [0.416;1.309]	1.76e-04
Un/de-differentiated	1.212 [0.288;2.137]	0.010
Carcinosarcoma	1.708 [0.794;2.621]	2.82e-04
Grade 1 (GR1)	-0.751 [-0.923;-0.580]	4.30e-16
Grade 2 (GR2)	-0.035 [-0.347;0.276]	0.824
Grade 3 (GR3)	0.794 [0.622;0.967]	2.38e-17
FIGO Stage I	-0.683 [-0.904;-0.463]	3.59e-09
FIGO Stage II	0.215 [-0.177;0.607]	0.282
FIGO Stage III	0.801 [0.548;1.053]	1.66e-09
LVSI presence	0.526 [0.237;0.814]	3.94e-04
*POLE*mut	-0.383 [-0.724;-0.042]	0.028
im*POLE*mut	-0.320 [-0.644;0.004]	0.053
MMRd	-0.208 [-0.430;0.014]	0.067
imMMRd	-0.228 [-0.442;-0.014]	0.037
p53abn	1.028 [0.827;1.229]	1.57e-20
imp53abn	1.253 [1.066;1.439]	2.22e-31
NSMP	-0.441 [-0.627;-0.256]	4.46e-06
imNSMP	-0.543 [-0.727;-0.359]	1.76e-08
ER negative	0.948 [0.708;1.187]	1.48e-13
L1CAM positive	1.007 [0.802;1.211]	2.31e-19

Table 7: Coefficients of single linear regression between continuous HECTOR risk scores and each categorical variable. Corresponding *P*-values of the linear regression are reported. CI = confidence interval; LVSI = lymphovascular space invasion; MMRd = Mismatch repair deficient; NSMP = no specific molecular profile; p53abn = p53 abnormal; im = image-based predicted from im4MEC[7].

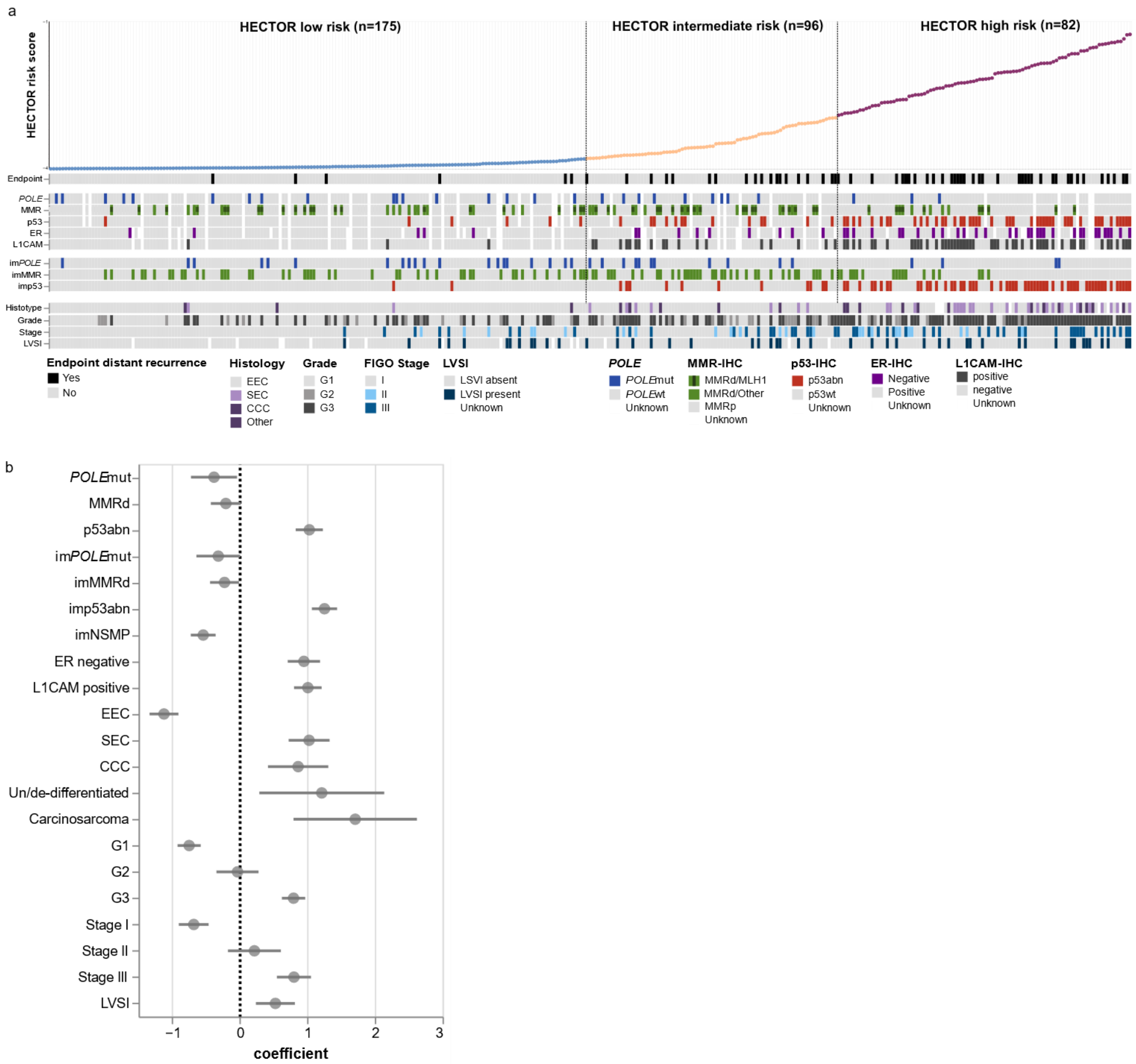

Figure 13: a, Extended heatmap of risk factors for patients included in the internal test set (n = 353) ordered by predicted HECTOR risk scores, along with their image-based molecular class as predicted by im4MEC[7]. b, Coefficients and confidence intervals of the risk factors derived from separate single linear regression models with the continuous risk score as the target variable. Data are presented as the coefficients of linear regression and 95% confidence interval (n = 353).

n=1408	im*POLE* mut	imMMRd	imp53abn	imNSMP
POLE mut	111	6	4	1
MMRd	8	367	6	3
p53abn	0	1	246	0
NSMP	2	5	8	525
Unkown	14	29	13	59

n=353	m*POLE* mu	imMMRd	imp53abn	imNSMP
POLE mut	11	13	1	3
MMRd	14	49	3	9
p53abn	2	1	43	7
NSMP	4	23	15	117
Unkown	4	12	5	17

n=96	*imPOLE* mut	imMMRd	imp53abn	imNSMP
POLE mut	1	2	0	0
MMRd	2	7	2	2
p53abn	0	0	38	4
NSMP	0	8	10	14
Unknown	1	0	4	1

Table 8: Confusion matrices of im4MEC[7] in the training set (left), internal test set (right), HECTOR high-risk of the internal test set (bottom right).

	HECTOR low-risk	HECTOR intermediate-risk	HECTOR high-risk	Total
**Total**	**175**	**82**	**96**	**353**
**Histotype**				
Endometrioid	170 (58%)	70 (24%)	51 (18%)	**291**
Serous	2 (6%)	7 (21%)	24 (73%)	**33**
Clear cells	0 (0%)	4 (24%)	13 (76%)	**17**
Un/de-differentiated	0 (0%)	0 (0%)	3 (100%)	**3**
Carcinosarcoma	0 (0%)	0 (0%)	3 (100%)	**3**
Other	3 (50%)	1 (17%)	2 (33%)	**6**
**Grade**				
1	123 (72%)	32 (19%)	15 (9%)	**170**
2	16 (39%)	17 (41%)	8 (20%)	**41**
3	36 (25%)	33 (23%)	73 (51%)	**142**
**2009 FIGO stage**				
IA	52 (55%)	17 (18%)	26 (27%)	**95**
IB	109 (60%)	44 (24%)	30 (16%)	**183**
II	6 (25%)	8 (33%)	10 (42%)	**24**
IIIA	2 (10%)	6 (29%)	13 (62%)	**21**
IIIB	0 (0%)	1 (11%)	8 (89%)	**9**
IIIC	6 (29%)	6 (29%)	9 (43%)	**21**
**LVSI**				
Present	12 (26%)	13 (28%)	22 (47%)	**47**
Absent or focal	150 (52%)	66 (23%)	74 (26%)	**290**
Unkown	13 (81%)	3 (19%)	0 (0%)	**16**
**Molecular class**				
POLE mut	17 (59%)	9 (31%)	3 (10%)	**29**
POLEwt	135 (47%)	64 (22%)	87 (30%)	**286**
MMRd	36 (46%)	29 (37%)	14 (18%)	**79**
MMRp	116 (49%)	44 (19%)	76 (32%)	**236**
p53abn	6 (9%)	13 (20%)	45 (70%)	**64**
p53wt	146 (58%)	60 (24%)	45 (18%)	**251**
Unkown	23 (61%)	9 (24%)	6 (16%)	**38**
**ER IHC**				
Negative	5 (11%)	9 (20%)	31 (69%)	**45**
Positive	144 (57%)	56 (22%)	52 (21%)	**252**
Unkown	26 (46%)	17 (30%)	13 (23%)	**56**
**L1CAM**				
Negative	153 (61%)	55 (22%)	42 (17%)	**250**
Positive	3 (4%)	18 (26%)	49 (70%)	**70**
Unkown	19 (58%)	9 (27%)	5 (15%)	**33**
**im4MEC**				
im*POLE* mut	20 (57%)	11 (31%)	4 (11%)	**35**
imMMRd	45 (46%)	36 (37%)	17 (17%)	**98**
imp53abn	2 (3%)	11 (16%)	54 (81%0	**67**
imNSMP	108 (71%)	24 (16%)	21 (14%)	**153**

Table 9: Composition of the HECTOR risk groups with clinicopathological prognostic factors and guideline prognostic groups. G = grade; EEC = endometrioid. LVSI = lymphovascular space invasion; *POLE*mut = *POLE* mutated; *POLE*wt = *POLE* wild type; MMRd = Mismatch repair deficient; MMRp = Mismatch repair proficient; NSMP = no specific molecular profile; p53abn = p53 abnormal; p53wt = p53 wild type; im = image-based predicted from im4MEC[7].

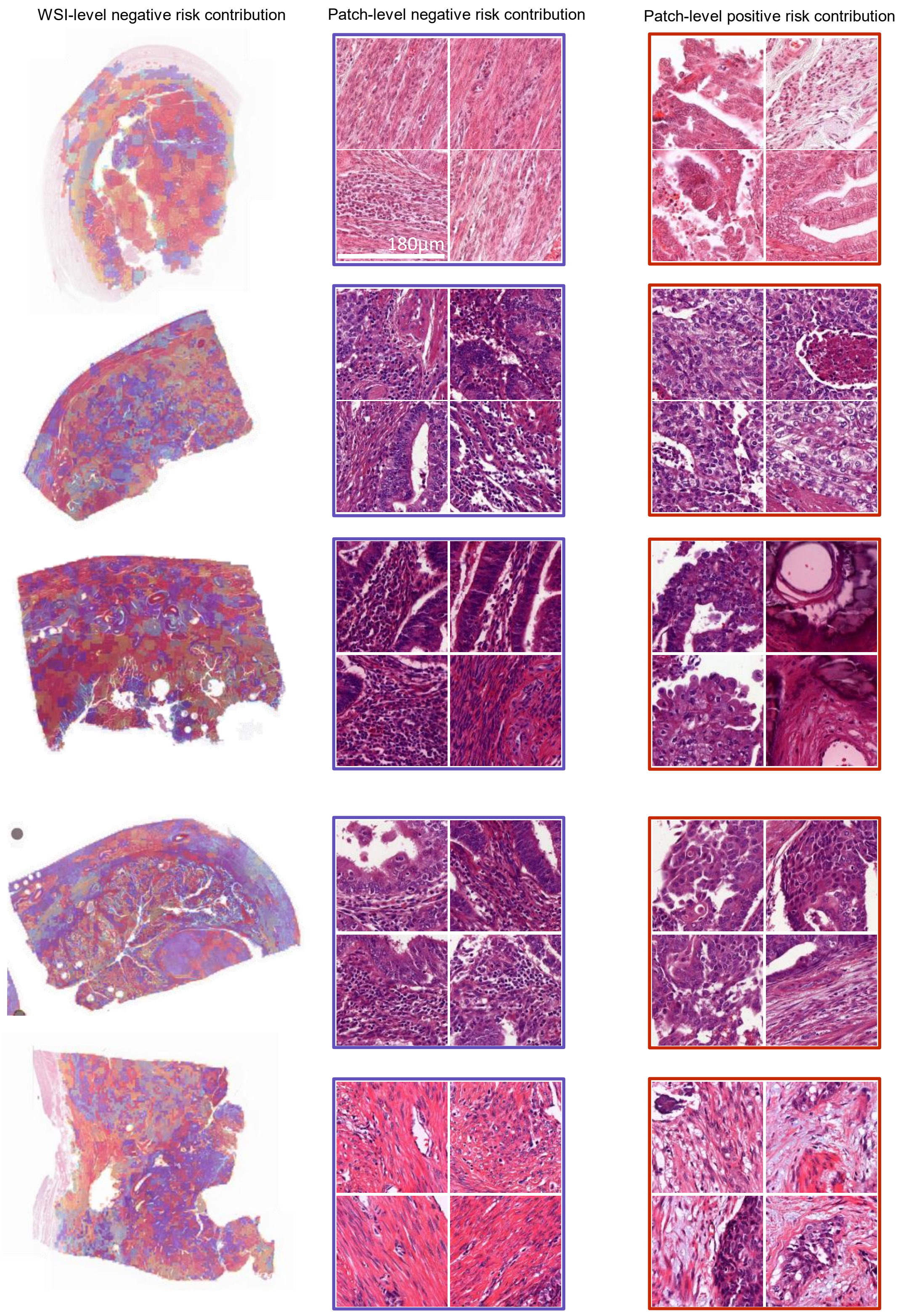

Figure 14: Cases with whole slide image (WSI)-level negative risk contribution as quantified using the Integrated Gradient (IG) method[14] (that is by avareging all patch-level contribution scores given by the IG method). Left shows the heatmap overlay of the contributions given to each patch. Middle and left show a selection of four patches with high negative (decreasing the risk) and positive contribution (increasing the risk). Selection of representative patches was performed once by an expert pathologist within the top 5% patches with highest IG scores. Each square patch is 180µm wide.

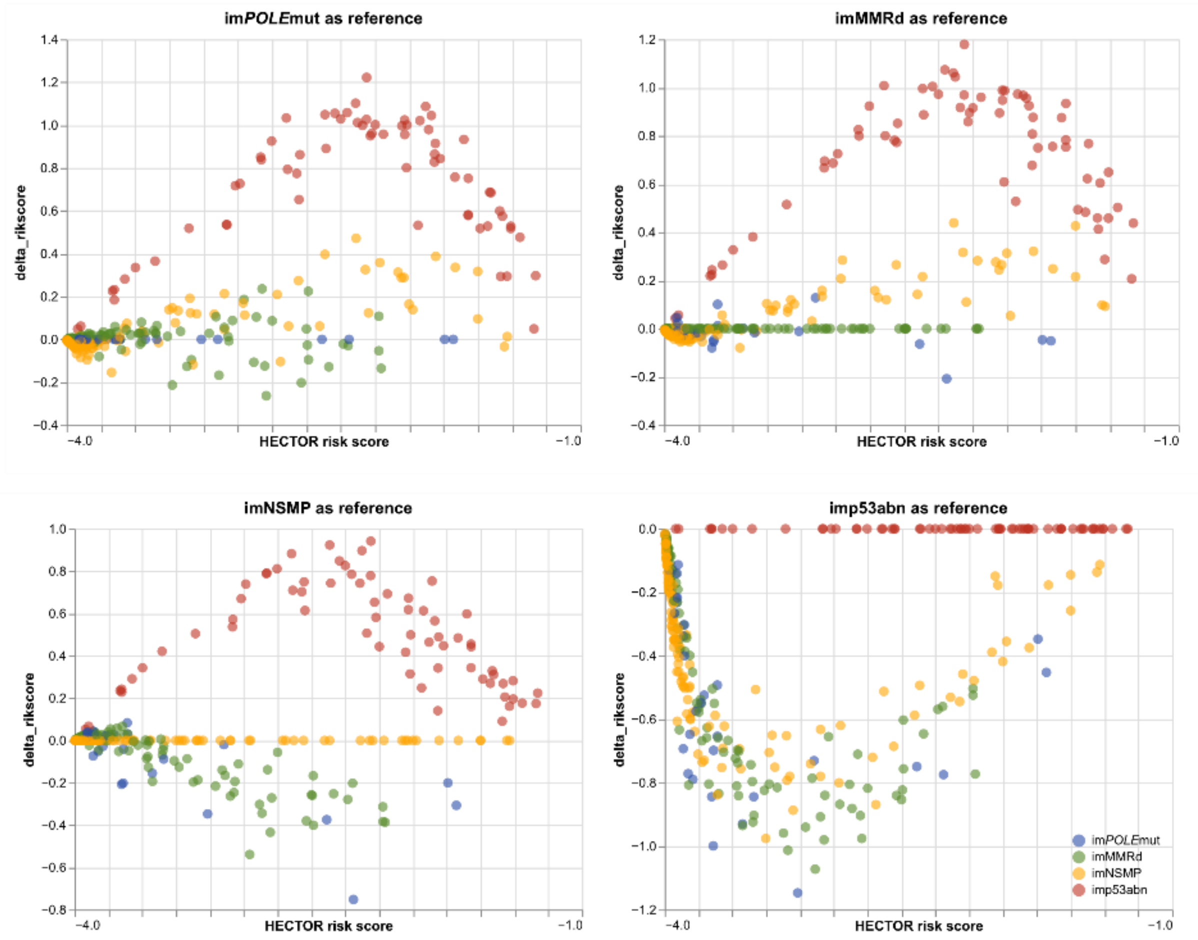

Figure 15: Contribution each the image-based molecular class (im4MEC) to the HECTOR risk scores. The difference in predicted HECTOR risk score is computed between the HECTOR risk score given by the image-based molecular class as precited by im4MEC[7] and the one given by hard-coding the image-based molecular class, referred to as "the reference group": (top left) im*POLE*mut is the reference group; (top right) imMMRd is the reference group; (bottom left) imNSMP is the reference group; (bottom right) imp53abn is the reference group. im = image-based; MMRd = Mismatch repair deficient; NSMP = no specific molecular profile; p53abn = p53 abnormal.

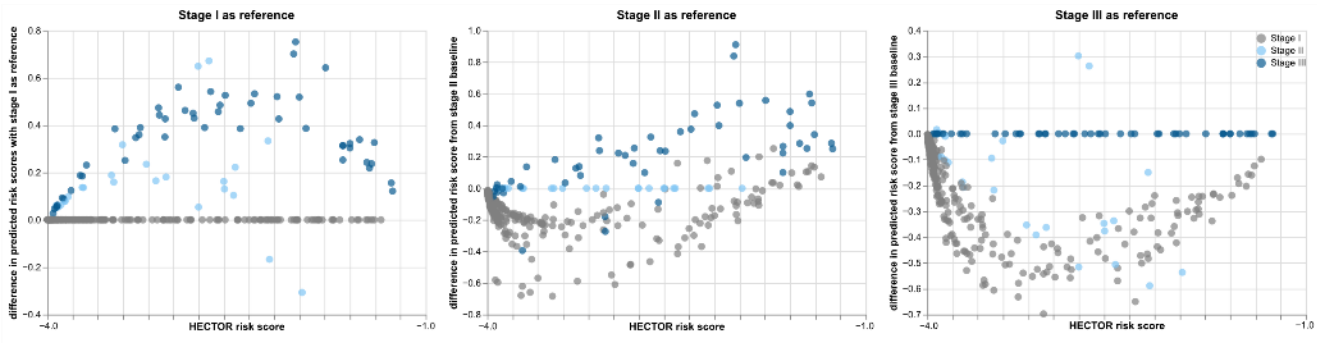

Figure 16: Contribution of the image-based molecular class as predicted by im4MEC to the HECTOR risk score. The difference in predicted risk score is computed between the risk score given by the FIGO stage and the reference group: (left) 2009 FIGO stage I is the reference group; (middle) 2009 FIGO stage II is the reference group; (bottom) 2009 FIGO stage III is the reference group.

	Registered histological subtype/LVSI	Review by expert of the single input WSI	2009 FIGO Stage	Image-based molecular class	HECTOR risk group	True molecular class	HECTOR risk group with true molecular class
Case 1	G1 EEC / no LVSI	Concordant	I	imNSMP	Low risk	*POLE*mut	Low risk
Case 2	G3 EEC / no LVSI	Concordant	I	im*POLE*mut	Low risk	MMRd	Low risk
Case 3	G3 EEC / no LVSI	Concordant	I	imNSMP	Low risk	NSMP	Low risk
Case 4	G1 EEC / LVSI	No LVSI	III	im*POLE*mut	Low risk	NSMP	Low risk
Case 5	G1 EEC / no LVSI	Concordant	II	im*POLE*mut	Low risk	NSMP	Low risk
Case 6	Other Non-EEC / Unknown	Endometrioid histology	I	im*POLE*mut	Low risk	Unknown	-

Table 10: Clinicopathological data and HECTOR risk group predictions of the cases predicted as HECTOR low risk that had distant recurrence in the internal test set (*n* = 6 out of 353 patients). The image-based (im) molecular class is produced by the im4MEC arm[7]. G1 EEC = Grade 1 Endometrioid; LVSI = lymphovascular space invasion; im = image-based; *POLE*mut = *POLE* mutated; NSMP = No specific molecular profile.

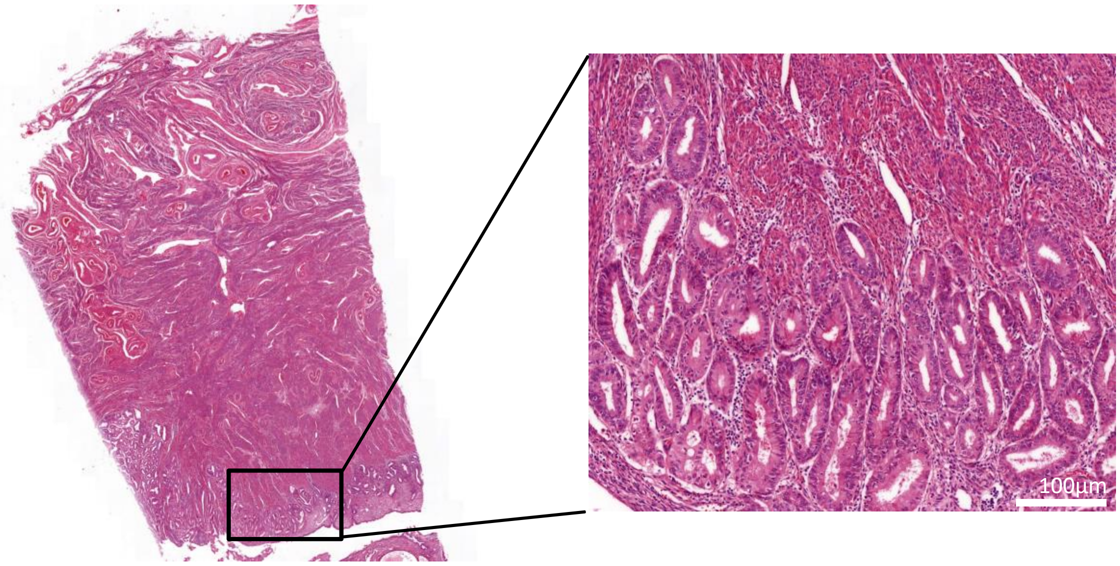

Figure 17: Example of a case with discrepancy between registered non-endometrioid histological subtype and the review of the input WSI by an expert, showing an endometrioid histology. Distant recurrence predicted HECTOR low risk in the internal test set (*n* = 353 patients).

	Registered histological subtype/LVSI	Review expert of the input WSI	2009 FIGO Stage	Image-based molecular class	HECTOR risk category	True molecular class	HECTOR risk category with true molecular class
Case 1	G3 EEC / no LVSI	Concordant	III	imMMRd	High risk	*POLE*mut	High risk
Case 2	G3 EEC / no LVSI	Concordant	II	im*POLE*mut	High risk	*POLE*mut	High risk
Case 3	Un/de-differentiated / LVSI	Concordant	III	imMMRd	High risk	*POLE*mut	High risk

Table 11: Clinicopathological data and HECTOR predictions of the *POLE*mut cases predicted as HECTOR high risk in the internal test set (n = 3 out of 353 patients). The image-based (im) molecular class is produced by the im4MEC arm[7]. G3 EEC = Grade 3 Endometrioid; LVSI = lymphovascular space invasion; im = image-based; *POLE*mut = *POLE* mutated; MMRd = Mismatch repair deficient.

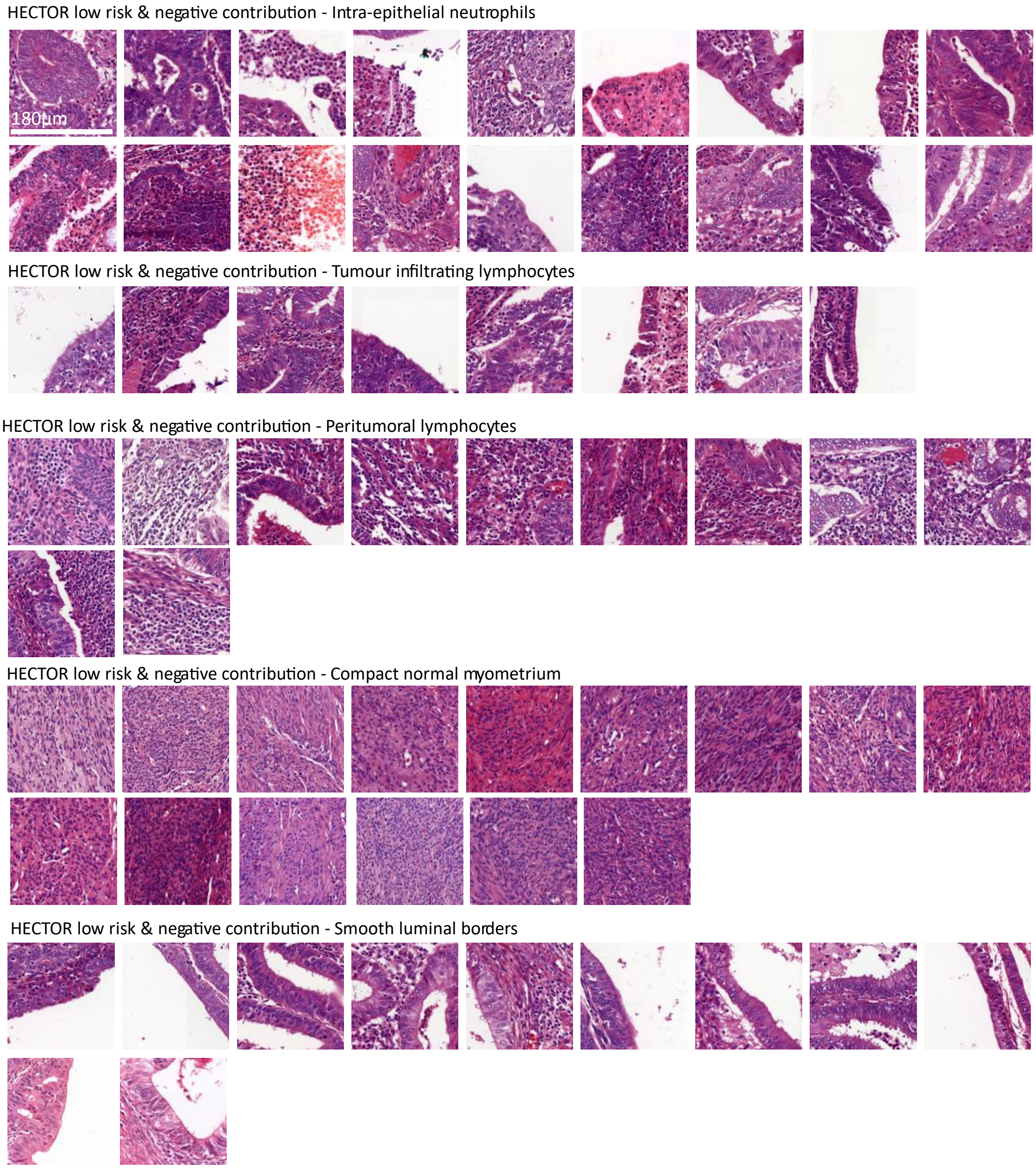

Figure 18: Additional representative patches which strongly decreased the HECTOR risk scores from all WSIs in the HECTOR low risk group. Selection of representative patches was performed once by an expert pathologist within the top 5% patches with negative contribution using the Integrated Gradient method[14]). Each square patch is 180µm wide.

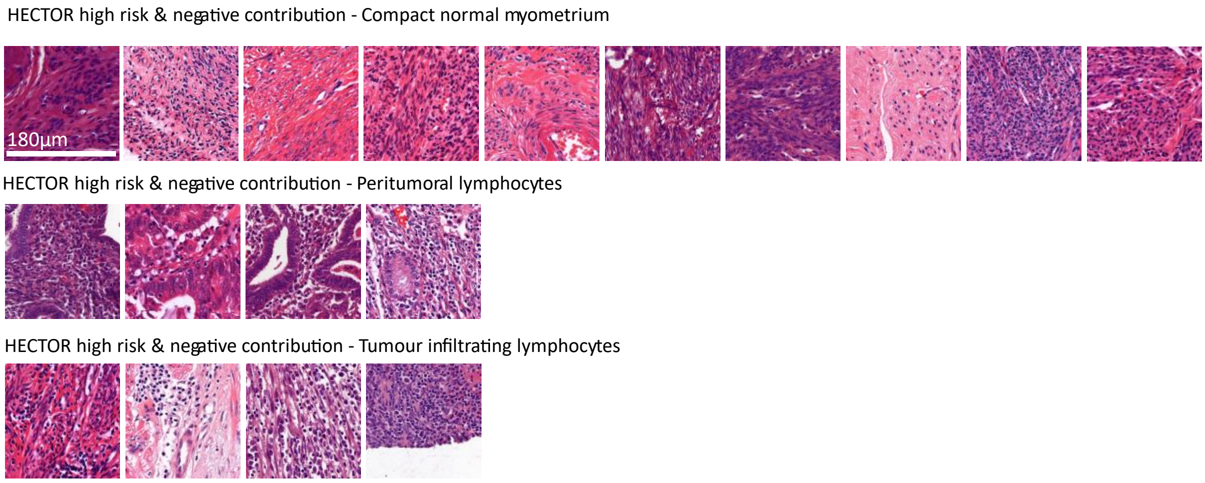

Figure 19: Additional representative patches which strongly decreased the HECTOR risk scores from all WSIs in the HECTOR high risk group. Selection of representative patches was performed once by an expert pathologist within the top 5% patches with negative contribution using the Integrated Gradient method[14]. Each square patch is 180µm wide.

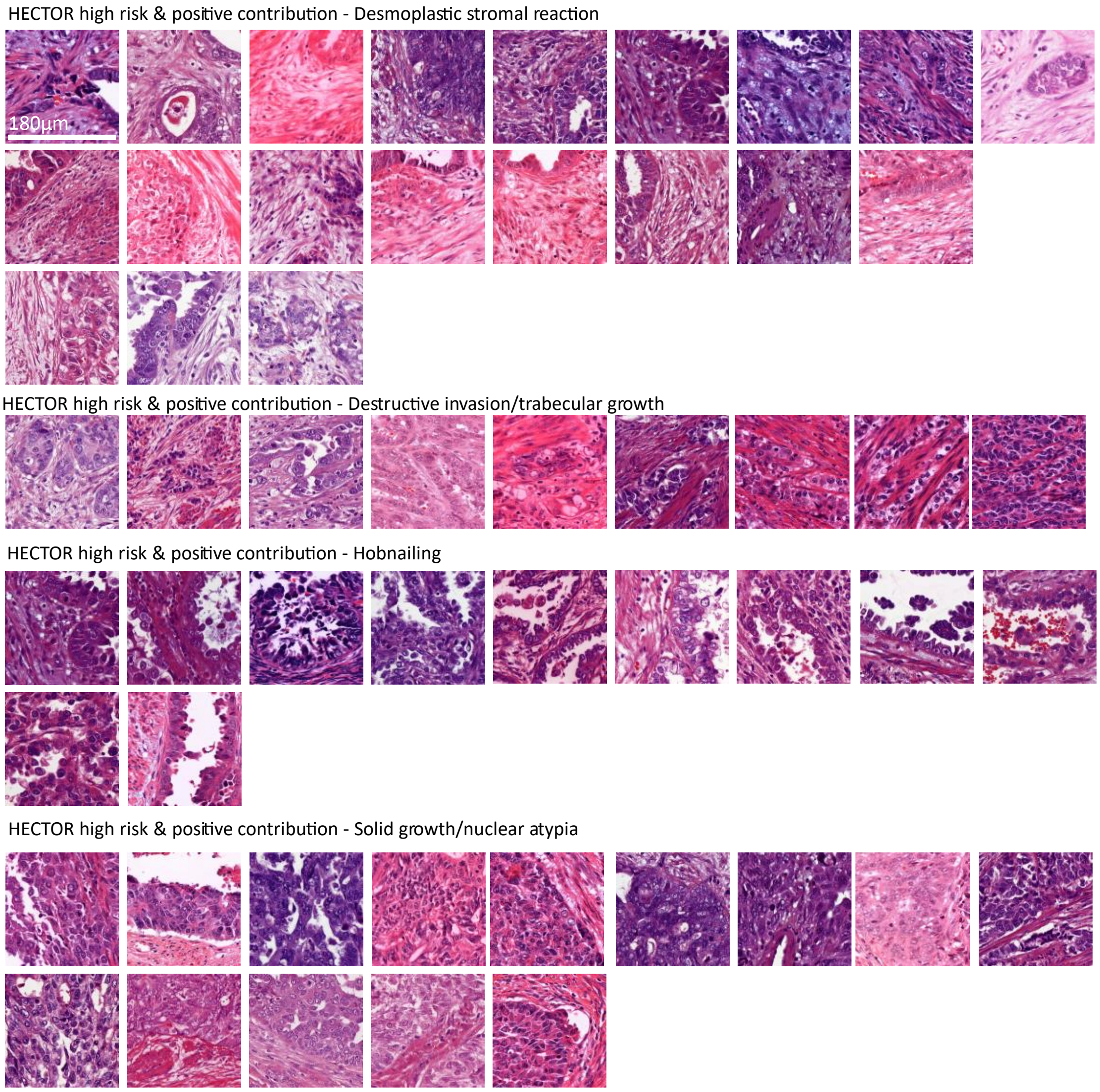

Figure 20: Additional representative patches which strongly increased the HECTOR risk scores from all WSIs in the HECTOR low risk group. Selection of representative patches was performed once by an expert pathologist within the top 5% patches with positive contribution using the Integrated Gradient method[14]). Each square patch is 180µm wide.

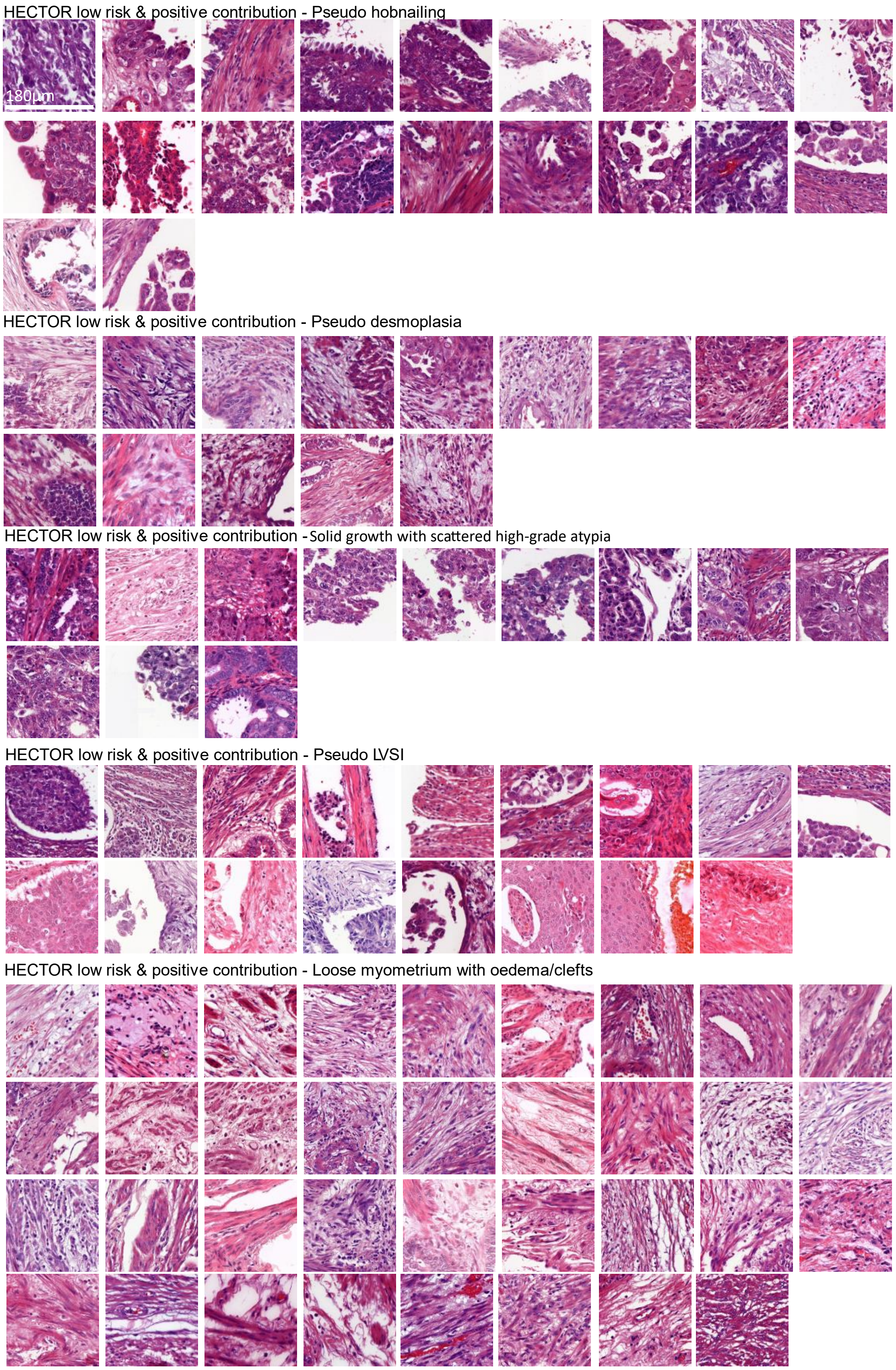

Figure 21: Additional representative patches which strongly increased the HECTOR risk scores from all WSIs in the HECTOR low risk group. Selection of representative patches was performed once by

an expert pathologist within the top 5% patches with positive contribution using the Integrated Gradient method[14]). Each square patch is 180µm wide.

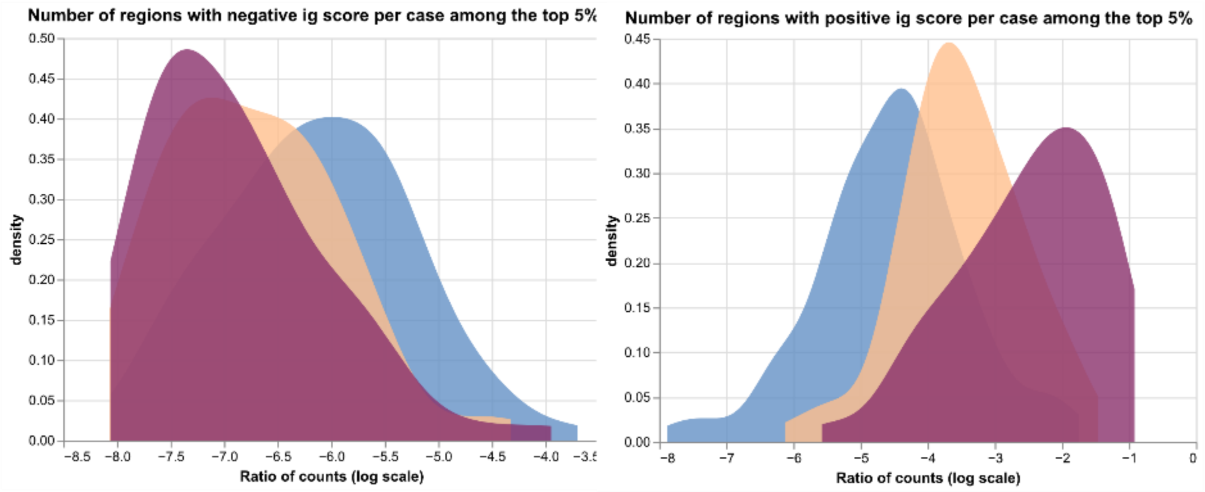

Figure 22: Density plots showing the patient-level ratio of the number of regions with negative or positive contribution among all top 5% regions with negative (left) or positive contribution (right) as quantified by the Integrated Gradient method[14].

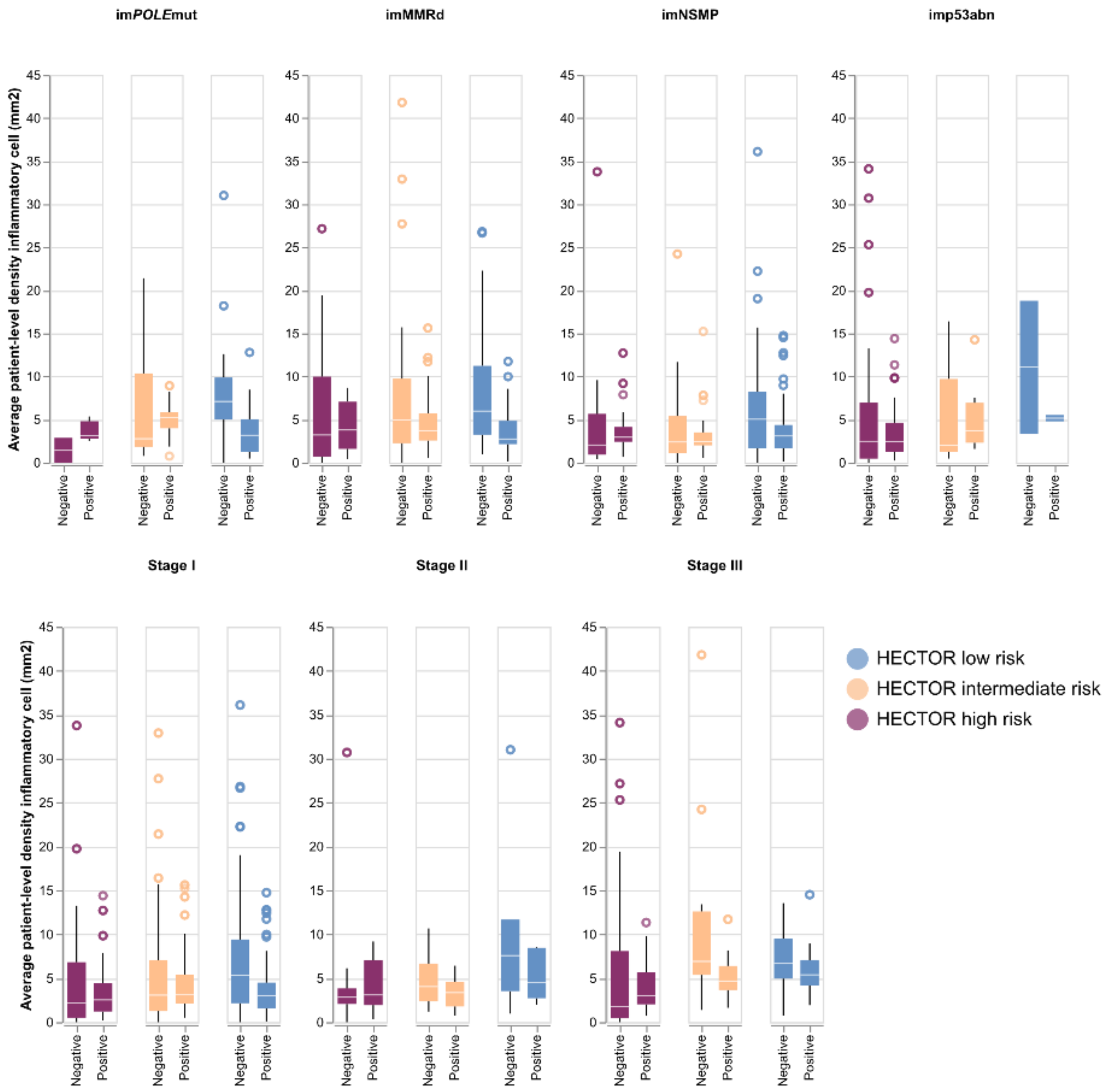

Figure 23: Average patient-level inflammatory cell density within the top 5% regions with positive and negative contribution, using the Integrated Gradient method[14], stratified by image-based molecular class (top) and 2009 FIGO stage I-III (bottom) in the internal test set (*n* = 353 cases of which 278 stage I, 24 stage II, 51 stage III; 35 im*POLE*mut, 98 imMMRd, 67 imp53abn, 153 imNSMP). The box plots are defined by the center tick as the median value, the lower and upper parts of the box as the first (Q1) and third (Q3) quartile respectively, the bounds of whiskers are [Q1 – 1.5 * IQR, Q3 + 1.5 * IQR] where IQR is the interquartile range (Q3-Q1). Any outlier points beyond the whisker are displayed with point marks.

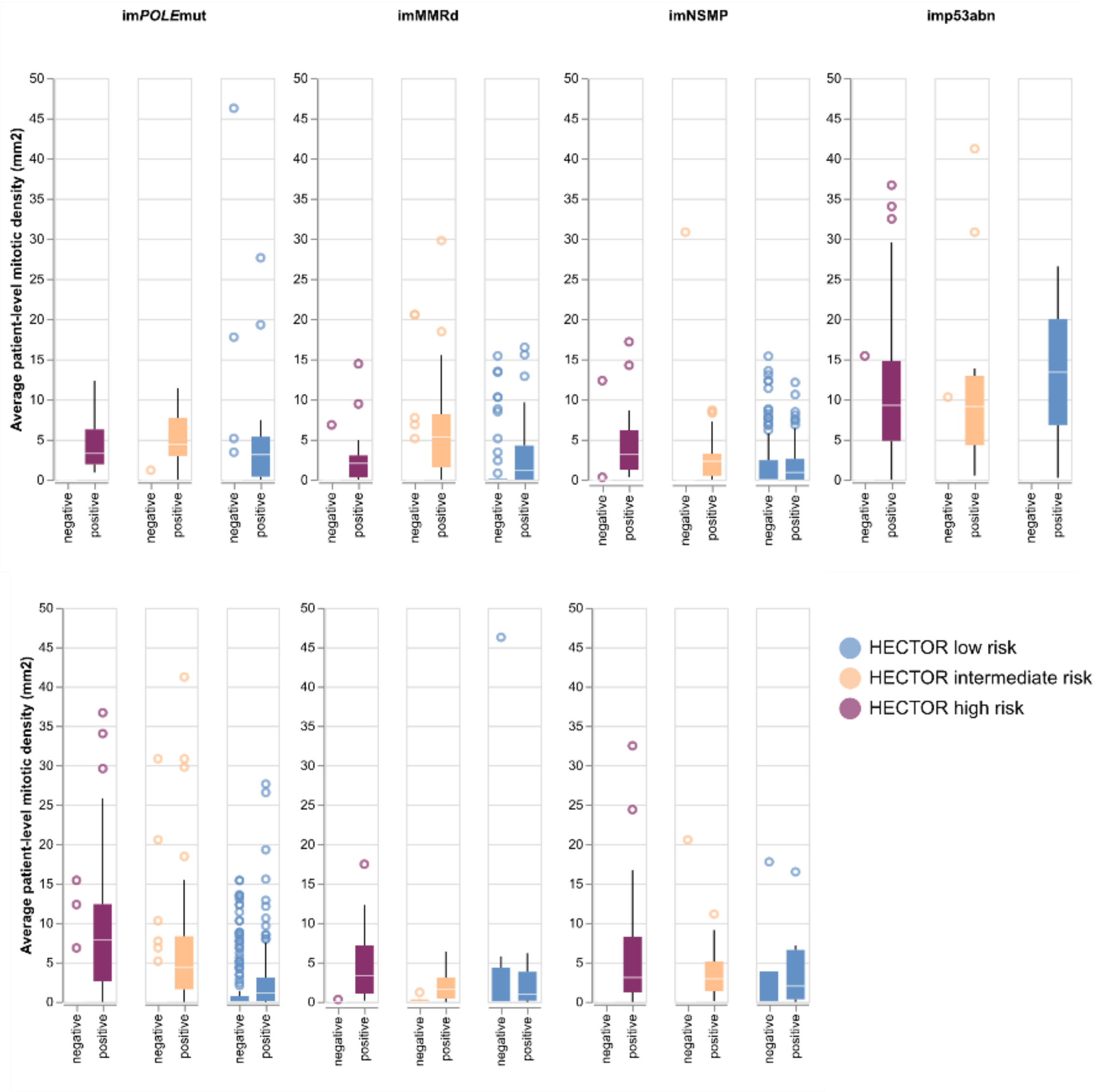

Figure 24: Average patient-level mitotic density within the top 5% regions with positive and negative Integrated Gradient[14] score filtered by regions, stratified by image-based molecular class[7] (top) and 2009 FIGO stage I-III (bottom) in the internal test set (*n* = 353 cases of which 278 stage I, 24 stage II, 51 stage III; 35 im*POLE*mut, 98 imMMRd, 67 imp53abn, 153 imNSMP).The box plots are defined by the center tick as the median value, the lower and upper parts of the box as the first (Q1) and third (Q3) quartile respectively, the bounds of whiskers are [Q1 – 1.5 * IQR, Q3 + 1.5 * IQR] where IQR is the interquartile range (Q3-Q1). Any outlier points beyond the whisker are displayed with point marks.

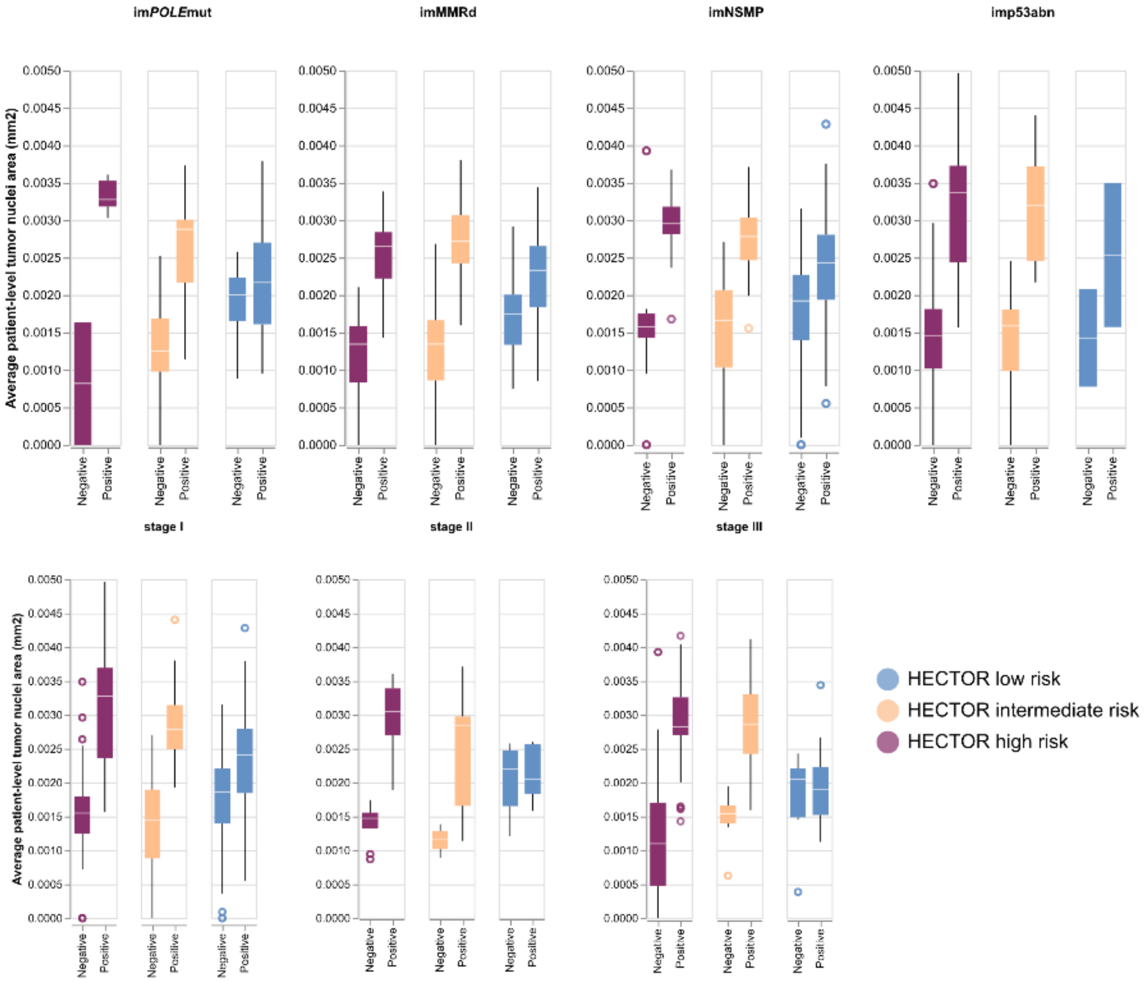

Figure 25: Average patient-level area of tumor nuclei within the top 5% regions with positive and negative Integrated Gradient[14] score filtered by regions, stratified by image-based molecular class[7] (top) and 2009 FIGO stage I-III (bottom) in the internal test set (*n* = 353 cases of which 278 stage I, 24 stage II, 51 stage III; 35 im*POLE*mut, 98 imMMRd, 67 imp53abn, 153 imNSMP).The box plots are defined by the center tick as the median value, the lower and upper parts of the box as the first (Q1) and third (Q3) quartile respectively, the bounds of whiskers are [Q1 – 1.5 * IQR, Q3 + 1.5 * IQR] where IQR is the interquartile range (Q3-Q1). Any outlier points beyond the whisker are displayed with point marks.

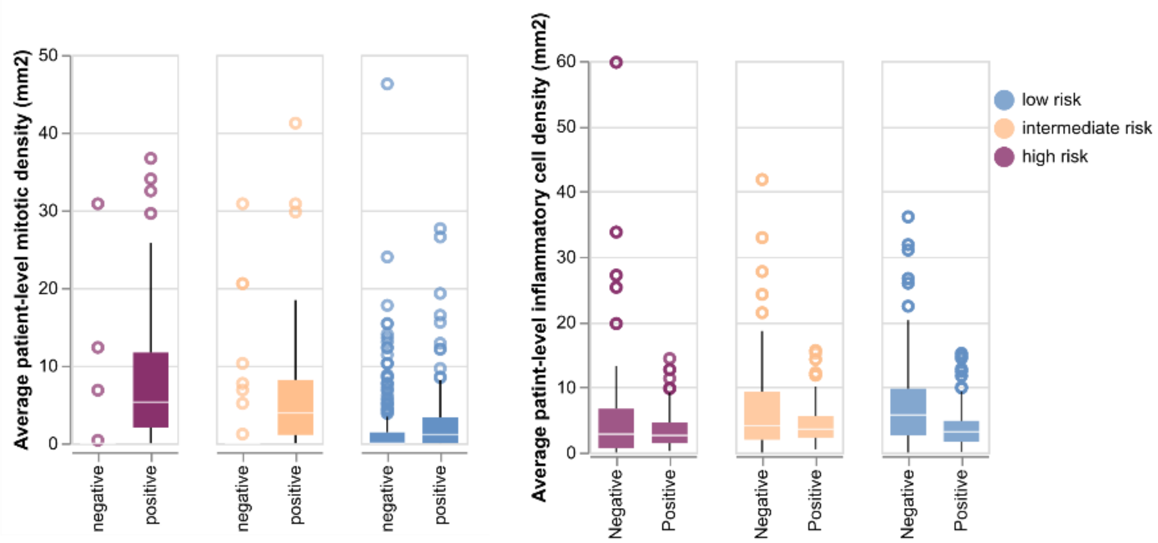

Figure 26: Average patient-level inflammatory cell count (left) and mitotic figure count (right) within the top 5% regions with positive and negative Integrated Gradient[14] score filtered by regions that contained tumour cells as predicted by Hover-Net[15] in the internal test set (*n* = 353 cases). The box plots are defined by the center tick as the median value, the lower and upper parts of the box as the first (Q1) and third (Q3) quartile respectively, the bounds of whiskers are [Q1 – 1.5 * IQR, Q3 + 1.5 * IQR] where IQR is the interquartile range (Q3-Q1). Any outlier points beyond the whisker are displayed with point marks.

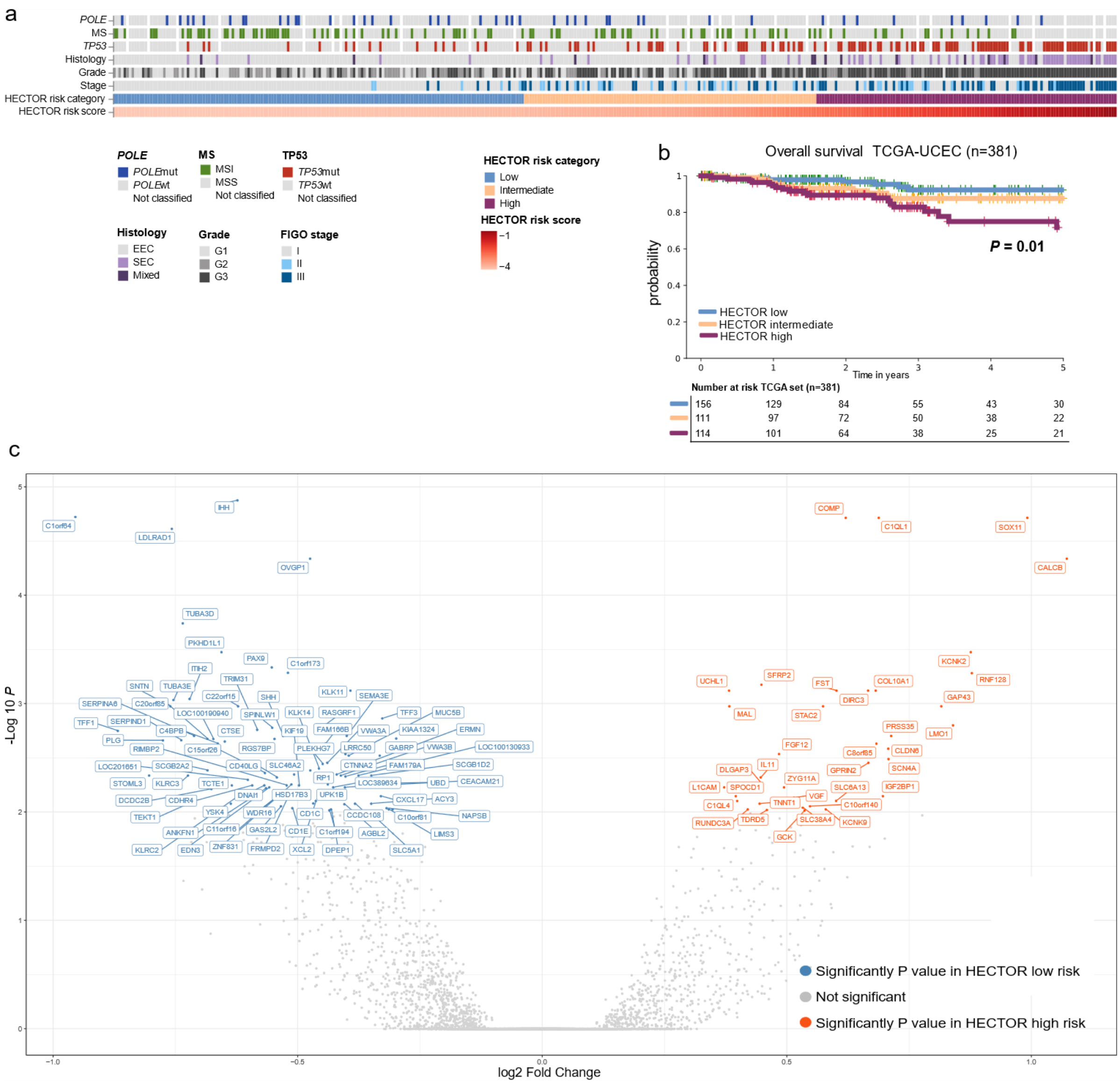

Figure 27: Analysis in FIGO stage I-III TCGA-UCEC (*n* = 381 patients) a, Association with clinicopathological data and HECTOR risk scores. b, Six-year overall survival analysis using the Kaplan Meier's method stratified by HECTOR risk group. Log-rank test *P*-value is reported. c, Differential gene expression analysis by DESeq2[16]. *P*-values of the likelihood ratio test were adjusted with Benjamini–Hochberg False Discovery Rate (FDR) and statistical significance accepted below 0.050. Exact *P* values are reported in Supplementary Table 15.

	Sample size	*P* value of test between HECTOR low, intermediate, high risk groups	*P* value of test between HECTOR low and high risk groups
ARID1A	Low risk (*n* = 72)<br>Intermediate risk (*n* = 50)<br>High risk (*n* = 27)	3.74e-05	6.11e-06
BCOR	Low risk (*n* = 10)<br>Intermediate risk (*n* = 11)<br>High risk (*n* = 5)	0.304	0.197
CTCF	Low risk (*n* = 39)<br>Intermediate risk (*n* = 20)<br>High risk (*n* = 11)	1.57e-04	7.50e-05
CTNNB1	Low risk (*n* = 47)<br>Intermediate risk (*n* = 26)<br>High risk (*n* = 11)	8.47e-06	2.28e-06
FAT1	Low risk (*n* = 16)<br>Intermediate risk (*n* = 11)<br>High risk (*n* = 7)	0.166	0.0606
FBXW7	Low risk (*n* = 25)<br>Intermediate risk (*n* = 14)<br>High risk (*n* = 19)	0.208	0.365
FGFR2	Low risk (*n* = 23)<br>Intermediate risk (*n* = 8)<br>High risk (*n* = 6)	9.12e-04	1.59e-03
KMT2C	Low risk (*n* = 14)<br>Intermediate risk (*n* = 5)<br>High risk (*n* = 9)	0.113	0.297
KMT2D	Low risk (*n* = 19)<br>Intermediate risk (*n* = 15)<br>High risk (*n* = 12)	0.447	0.209
KRAS	Low risk (*n* = 30)<br>Intermediate risk (*n* = 21)<br>High risk (*n* = 8)	1.99e-03	3.59e-04
NOTCH1	Low risk (*n* = 1)<br>Intermediate risk (*n* = 4)<br>High risk (*n* = 0)	0.180	-
NSD1	Low risk (*n* = 13)<br>Intermediate risk (*n* = 11)<br>High risk (*n* = 5)	0.166	0.0593
PIK3CA	Low risk (*n* = 79)<br>Intermediate risk (*n* = 51)<br>High risk (*n* = 41)	1.11e-03	5.23e-04
PIK3R1	Low risk (*n* = 47)<br>Intermediate risk (*n* = 30)<br>High risk (*n* = 17)	7.29e-04	1.77e-04
PPP2R1A	Low risk (*n* = 1)<br>Intermediate risk (*n* = 8)<br>High risk (*n* = 15)	2.19e-03	4.65e-04

PTEN	Low risk (*n* = 117)<br>Intermediate risk (*n* = 71)<br>High risk (*n* = 39)	1.51e-09	4.24e-10
SPOP	Low risk (*n* = 11)<br>Intermediate risk (*n* = 6)<br>High risk (*n* = 7)	0.417	0.346
TP53	Low risk (*n* = 17)<br>Intermediate risk (*n* = 29)<br>High risk (*n* = 62)	2.81e-07	4.13e-07
ZFHX3	Low risk (*n* = 20)<br>Intermediate risk (*n* = 18)<br>High risk (*n* = 9)	0.112	0.0411

Table 12: *P* values of the two-sided Chi square tests to compare proportions with oncogenic mutations between HECTOR risk groups on an individual gene basis. Gene symbols are shown in italic.

	Unadjusted		Adjusted for EC molecular class		Adjusted for EC molecular class and TMB	
Immune cells	Coefficient	*P* value	Coefficient	*P* value	Coefficient	*P* value
B cells memory	0.068	8.47e-3	0.0474	0.137	ND	ND
Denditric cells activated	0.078	7.88e-4	0.0564	0.0419	ND	ND
Denditric cells resting	-0.031	0.0492	-0.00343	0.857	0.0588	0.0355
Mast cells resting	0.058	0.0287	0.0255	0.417	ND	ND
CD8 t cells	-0.247	6.89e-6	-0.155	0.0163	-0.165	0.011
T cells follicular helper	-0.178	1.53e-4	-0.131	0.0161	-0.147	6.71e-3
T cells regulatory T regs	-0.251	2.21e-09	-0.199	8.02e-5	-0.196	1.19e-4

Table 13: Coefficients of linear regressions performed with the log2-transformed proportion of cell subset (Leukocyte_Fraction*immune_subset) as the dependent variable, and the the HECTOR risk scores as independent variables in the TCGA-UCEC (*n* = 381). Linear tegressions were adjusted for molecular subgroup and tumor mutational burden (TMB) by adding these two variables as independent variables. Corresponsing *P*-values of the linear regressions are reported.

	Inclusion	PORTEC-3
**Number of patients (%)**		**660**
	yes	442 (67.0%)
	no	218 (33,0%)
**Follow-up: median**		
	yes	5.06
	no	7.66
**Age: median**		
	yes	62
	no	68
**Histotype (%)**		
Endometrioid grade 1-2	yes	177 (26.8%)
	no	80 (12.1%)
Endometrioid grade 3	yes	127 (19.2%)
	no	58 (8.8%)
Serous carcinoma	yes	64 (9.7%)
	no	41 (6.2%)
Clear cell carcinoma	yes	42 (6.4%)
	no	20 (3.0%)
Carcinosarcoma	yes	0 (0.0%)
	no	0 (0.0%)
Un-dedifferentiated	yes	10 (1.5%)
	no	4 (0.6%)
Other	yes	22 (3.3%)
	no	15 (2.3%)
**LVSI (%)**		
Present	yes	280 (42.4%)
	no	109 (16.5%)
Focal or absent	yes	162 (24.5%)
	no	109 (16.5%)
**2009 FIGO stage (%)**		
IA	yes	56 (8.5%)
	no	22 (3.3%)
IB	yes	79 (12.0%)
	no	38 (5.8%)
II	yes	114 (17.3%)
	no	56 (8.5%)
IIIA	yes	50 (7.6%)
	no	33 (5.0%)
IIIB	yes	31 (4.7%)
	no	11 (1.7%)
IIIC	yes	112 (17.0%)
	no	58 (8.8%)
IV	yes	0 (0.0%)
	no	0 (0.0%)
**Molecular class (%)**		
POLE mut	yes	50 (7.6%)
	no	1 (0.2%)
MMRd	yes	136 (20.6%)
	no	3 (0.5%)
NSMP	yes	120 (18.2%)
	no	2 (0.3%)
p53abn	yes	88 (13.3%)
	no	11 (1.7%)
Unkown	yes	48 (7.3%)
	no	201 (30.5%)
**Adjuvant treatment (%)**		
EBRT alone	yes	214 (32.4%)
	no	116 (17.6%)
Chemotherapy + EBRT	yes	228 (34.5%)
	no	102 (15.5%)

Table 14: Characteristics of the PORTEC-3 randomized trial used for the analysis of treatment response by HECTOR. *POLE*mut = *POLE* mutant; MMRd = mismatch repair deficient; NSMP = No specific molecular profile; p53abn = p53 abnormal; EBRT = external beam radiotherapy.

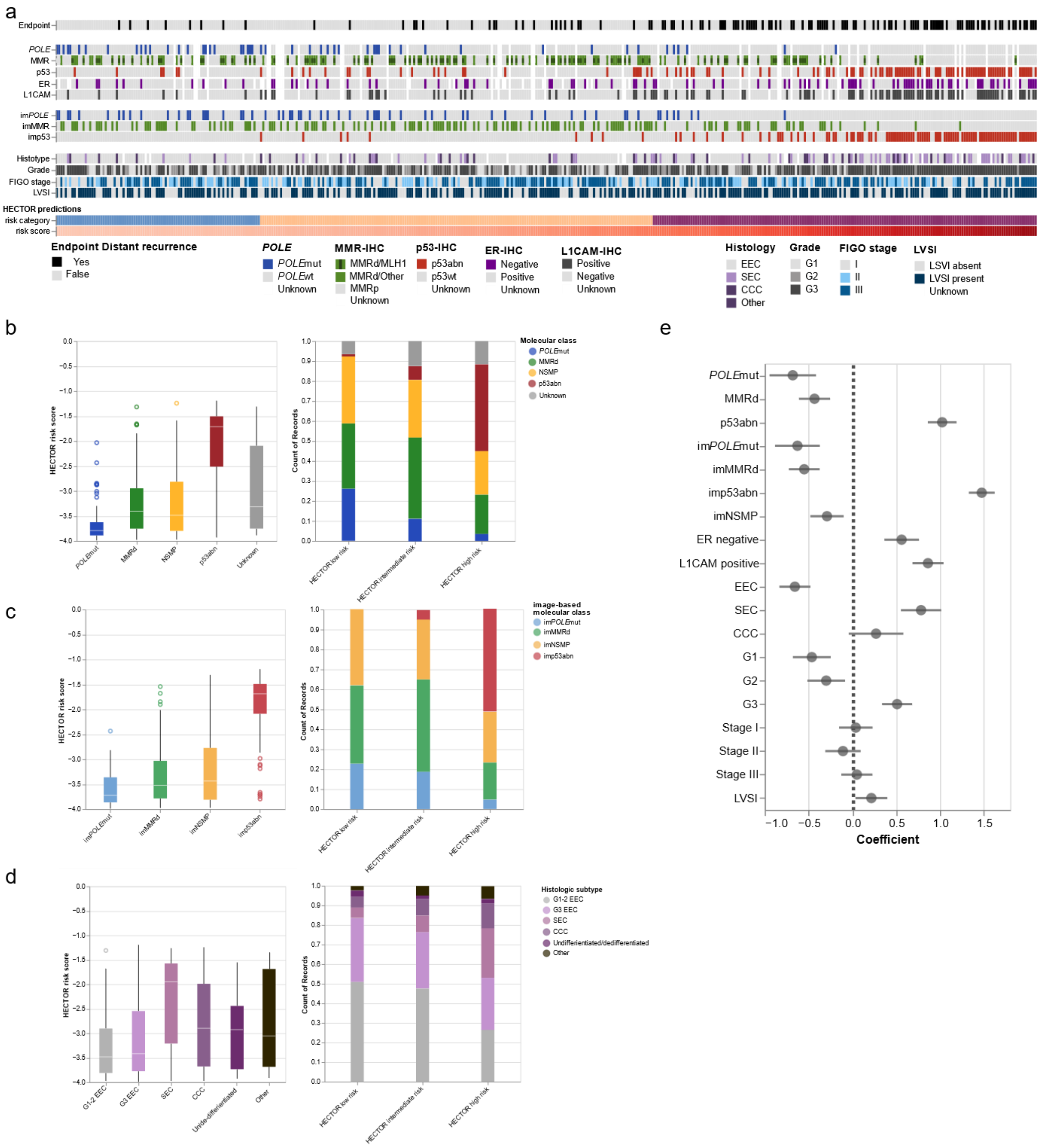

Figure 28: Association between HECTOR risk scores and clinicopathological data in the PORTEC-3 randomized trial (*n* = 442 patients). a, Heatmap of prognostic factors for patients ordered by predicted risk scores. b, Association between the molecular class of endometrial cancer and HECTOR risk scores (left) as continuous (right) as categorical using cutoffs of the training set in the PORTEC-3 (*n* = 442 patients). c, Association between the image-based molecular class as predicted by im4MEC and HECTOR risk scores (left) as continuous (right) as categorical using cutoffs of the training set in the PORTEC-3 (*n* = 442 patients). d, Association between histologic subtypes and HECTOR risk scores (left) as continuous (right) as categorical using cutoffs of the training set in the PORTEC-3 (*n* = 442 patients). e, Association of the clinicopathological data and continuous HECTOR risk scores using single linear regression. Data are presented as the coefficients of linear regression and 95% confidence interval (*n* = 442 patients). In b-d, the box plots are defined by the center tick as the median value, the lower and

upper parts of the box as the first (Q1) and third (Q3) quartile respectively, the bounds of whiskers are [Q1 – 1.5 * IQR, Q3 + 1.5 * IQR] where IQR is the interquartile range (Q3-Q1). Any outlier points beyond the whisker are displayed with point marks. *POLE*mut = *POLE* mutated; MMRd = mismatch repair deficient; p53abn = p53 abnormal; im = image-based; ER = estrogen receptor; EEC = endometrioid EC; SEC = serous; CCC = clear cell; G1-3 = grade 1-3.

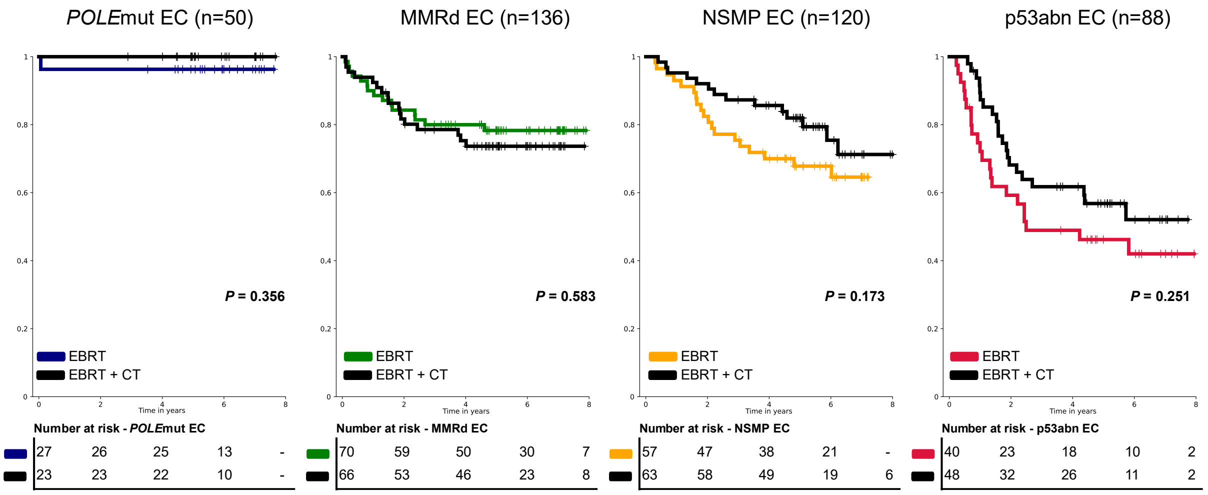

Figure 29: Eight-year distant recurrence-free probabilities of molecularly classified patients from the PORTEC-3 randomized trial by molecular class (*POLE*mut EC, MMRd EC, NSMP EC, p53abn EC). PORTEC-3 patients were randomized (1:1) between external beam radiotherapy (EBRT) and external beam radiotherapy and chemotherapy (CT). Analysis was performed with Kaplan Meier method and log-rank test *P*-values are reported. *POLE*mut = *POLE* mutant; MMRd = mismatch repair deficient; NSMP = No specific molecular profile; p53abn = p53 abnormal; EC = endometrial cancer.

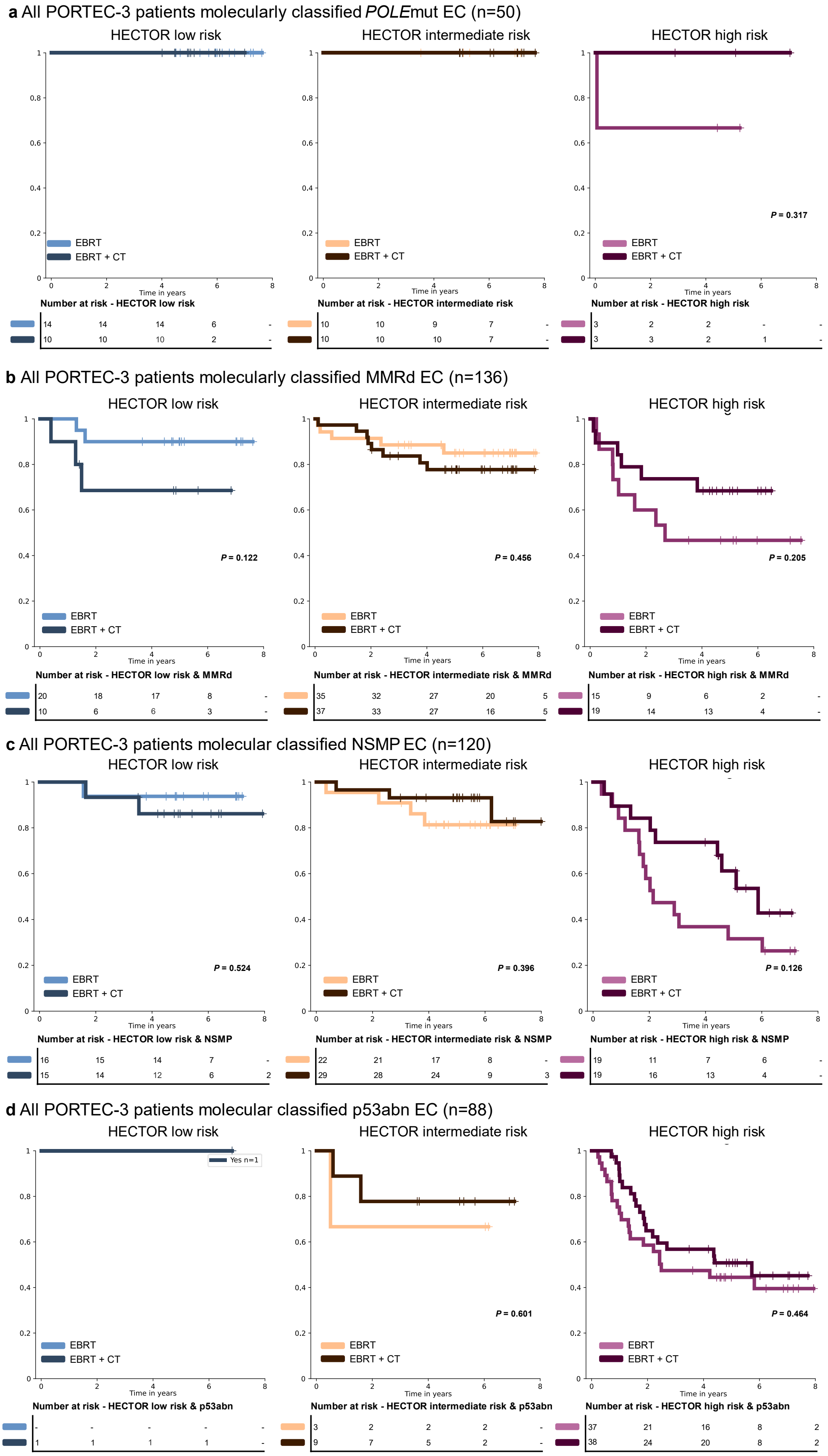

Figure 30: Eight-year distant recurrence-free probabilities of patients from the PORTEC-3 randomized trial by HECTOR risk group in the context of the true molecular classification of endometrial cancer

(EC), *POLE*mut, MMRd, NSMP or p53abn EC. PORTEC-3 patients were randomized (1:1) between external beam radiotherapy (EBRT) and external beam radiotherapy and chemotherapy (CT). Only patients with known molecular class were retained (*n* = 394); for reference Figure 29 shows the analysis by molecular class. Analysis was done using Kaplan Meier method and log-rank test *P*-values reported. a, All PORTEC-3 patients molecularly classified as *POLE*mut EC (*n* = 50) and HECTOR low (*n* = 24), intermediate (*n* = 20) and high risk group (*n* = 6), stratified by treatment arm. b, All PORTEC-3 patients molecularly classified as MMRd EC (*n* = 136) and HECTOR low (*n* = 30), intermediate (*n* = 72) and high risk group (*n* = 34), stratified by treatment arm. c, All PORTEC-3 patients molecularly classified as NSMP EC (*n* = 120) and HECTOR low (*n* = 31), intermediate (*n* = 51) and high risk group (*n* = 38), stratified by treatment arm. d, All PORTEC-3 patients molecularly classified as p53abn EC (*n* = 88) and HECTOR low (*n* = 1), intermediate (*n* = 12) and high risk group (*n* = 75), stratified by treatment arm. Log-rank test p-values are reported. *POLE*mut = *POLE* mutant; MMRd = mismatch repair deficient; NSMP = No specific molecular profile; p53abn = p53 abnormal.

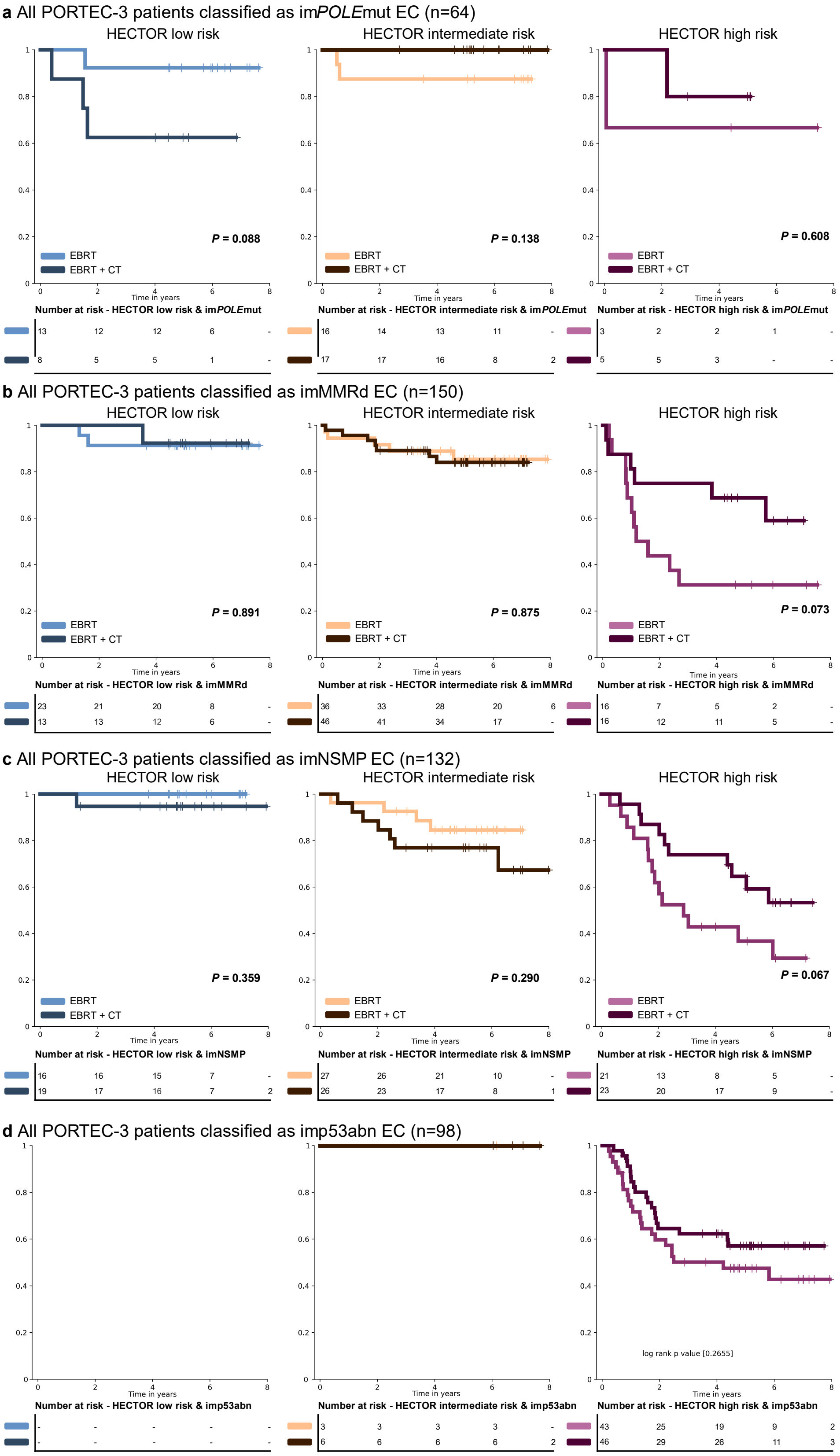

Figure 31: Eight-year distant recurrence-free probabilities of patients from the PORTEC-3 randomized trial by HECTOR risk group and stratified by the image-based (im) molecular classification arm of HECTOR, (as predicted by im4MEC[7]). PORTEC-3 patients were randomized (1:1) between external beam radiotherapy (EBRT) and external beam radiotherapy and chemotherapy (CT). a, The PORTEC-3

patients predicted as im*POLE*mut (*n* = 64) and by HECTOR low (*n* = 21), intermediate (*n* = 33) and high risk group (*n* = 8), stratified by treatment arm. Analysis was done using Kaplan Meier method and log-rank test *P*-values reported. b, The PORTEC-3 patients predicted as imMMRd (*n* = 150) and HECTOR low (*n* = 35), intermediate (*n* = 82) and high risk group (*n* = 32), stratified by treatment arm. c, The PORTEC-3 patients predicted as imNSMP (*n* = 132) and HECTOR low (*n* = 35), intermediate (*n* = 53) and high risk group (*n* = 44), stratified by treatment arm. d, The PORTEC-3 patients predicted as imp53abn (*n* = 98) and HECTOR low (*n* = 0), intermediate (*n* = 9) and high risk group (*n* = 89), stratified by treatment arm. Log-rank test p-values are reported. *POLE*mut = *POLE* mutant; MMRd = mismatch repair deficient; NSMP = No specific molecular profile; p53abn = p53 abnormal.

1. Li, C. *et al.* Efficient Self-supervised Vision Transformers for Representation Learning. *Int. Conf. Learn. Represent. ICLR* (2022).

2. Wang, X. *et al.* Transformer-based unsupervised contrastive learning for histopathological image classification. *Med. Image Anal.* **81**, 102559 (2022).

3. Ilse, M., Tomczak, J. & Welling, M. Attention-based Deep Multiple Instance Learning. in *Proceedings of the 35th International Conference on Machine Learning* (eds. Dy, J. & Krause, A.) vol. 80 2127–2136 (PMLR, 2018).

4. Zadeh, S. G. & Schmid, M. Bias in Cross-Entropy-Based Training of Deep Survival Networks. *IEEE Trans. Pattern Anal. Mach. Intell.* **43**, 3126–3137 (2021).

5. Lee, Y. *et al.* Derivation of prognostic contextual histopathological features from whole-slide images of tumours via graph deep learning. *Nat. Biomed. Eng.* (2022) doi:10.1038/s41551-022-00923-0.

6. Wagner, S. J. *et al.* Transformer-based biomarker prediction from colorectal cancer histology: A large-scale multicentric study. *Cancer Cell* **41**, 1650-1661.e4 (2023).

7. Fremond, S. *et al.* Interpretable deep learning model to predict the molecular classification of endometrial cancer from haematoxylin and eosin-stained whole-slide images: a combined analysis of the PORTEC randomised trials and clinical cohorts. *Lancet Digit. Health* **5**, e71–e82 (2023).

8. Pecorelli, S. Revised FIGO staging for carcinoma of the vulva, cervix, and endometrium. *Int. J. Gynaecol. Obstet. Off. Organ Int. Fed. Gynaecol. Obstet.* **105**, 103–104 (2009).

9. Uno, H., Cai, T., Pencina, M. J., D'Agostino, R. B. & Wei, L. J. On the C-statistics for evaluating overall adequacy of risk prediction procedures with censored survival data. *Stat. Med.* **30**, 1105–1117 (2011).

10. Lambert, J. & Chevret, S. Summary measure of discrimination in survival models based on cumulative/dynamic time-dependent ROC curves. *Stat. Methods Med. Res.* **25**, 2088–2102 (2016).

11. Graf, E., Schmoor, C., Sauerbrei, W. & Schumacher, M. Assessment and comparison of prognostic classification schemes for survival data. *Stat. Med.* **18**, 2529–2545 (1999).

12. Chen, R. J. *et al.* Pan-cancer integrative histology-genomic analysis via multimodal deep learning. *Cancer Cell* **40**, 865-878.e6 (2022).

13. Concin, N. *et al.* ESGO/ESTRO/ESP guidelines for the management of patients with endometrial carcinoma. *Int. J. Gynecol. Cancer Off. J. Int. Gynecol. Cancer Soc.* **31**, 12–39 (2021).

14. Sundararajan, M., Taly, A. & Yan, Q. Axiomatic Attribution for Deep Networks. in *Proceedings of the 34th International Conference on Machine Learning* (eds. Precup, D. & Teh, Y. W.) vol. 70 3319–3328 (PMLR, 2017).

15. Graham, S. *et al.* Hover-Net: Simultaneous segmentation and classification of nuclei in multi-tissue histology images. *Med. Image Anal.* **58**, 101563 (2019).

16. Love, M. I., Huber, W. & Anders, S. Moderated estimation of fold change and dispersion for RNA-seq data with DESeq2. *Genome Biol.* **15**, 550 (2014).
