# Supplementary material for: Prediction of recurrence risk in endometrial cancer with multimodal deep learning
Source: Nat Med. 2024 May 24;30(7):1962–73. doi: 10.1038/s41591-024-02993-w (PMC11271412; doi:10.1038/s41591-024-02993-w)
Supplement: Supplementary file 2 — Reporting Summary [file 41591_2024_2993_MOESM2_ESM.pdf]

nature portfolio

Corresponding author(s): Dr. Tjalling Bosse

Last updated by author(s): Apr 4, 2024

Reporting Summary

Nature Portfolio wishes to improve the reproducibility of the work that we publish. This form provides structure for consistency and transparency in reporting. For further information on Nature Portfolio policies, see our Editorial Policies and the Editorial Policy Checklist.

Statistics

For all statistical analyses, confirm that the following items are present in the figure legend, table legend, main text, or Methods section.

n/a	Confirmed	
☐	☒	The exact sample size (*n*) for each experimental group/condition, given as a discrete number and unit of measurement
☐	☒	A statement on whether measurements were taken from distinct samples or whether the same sample was measured repeatedly
☐	☒	The statistical test(s) used AND whether they are one- or two-sided<br>*Only common tests should be described solely by name; describe more complex techniques in the Methods section.*
☐	☒	A description of all covariates tested
☐	☒	A description of any assumptions or corrections, such as tests of normality and adjustment for multiple comparisons
☐	☒	A full description of the statistical parameters including central tendency (e.g. means) or other basic estimates (e.g. regression coefficient) AND variation (e.g. standard deviation) or associated estimates of uncertainty (e.g. confidence intervals)
☐	☒	For null hypothesis testing, the test statistic (e.g. *F*, *t*, *r*) with confidence intervals, effect sizes, degrees of freedom and *P* value noted<br>*Give P values as exact values whenever suitable.*
☐	☒	For Bayesian analysis, information on the choice of priors and Markov chain Monte Carlo settings
☐	☒	For hierarchical and complex designs, identification of the appropriate level for tests and full reporting of outcomes
☐	☒	Estimates of effect sizes (e.g. Cohen's *d*, Pearson's *r*), indicating how they were calculated

Our web collection on statistics for biologists contains articles on many of the points above.

Software and code

Policy information about availability of computer code

Data collection	Scanning of images was conducted with the 3D Histech P250 and P1000 scanner at 40x magnification. Images were read and pre-processed with Openslide Python package (version 1.1.2), OpenCV (version 4.3.0.36), and Pillow (version 7.2.0). Annotations were done with QuPath (version 0.4.1).
Data analysis	The custom deep learning model (HECTOR) was developed and trained using Pytorch (version 1.8.1 for the self-supervised learning and version 1.10.0 otherwise). Integrated Gradient was implemented with Captum Python package (version 0.6.0); metrics such as the concordance-index with scikit-survival Python package (version 0.17.2); Cox Proportional Hazard models and Kaplan Meier's method with Lifelines Python package (version 0.27.1); Chi square tests with Scipy Python package (version 1.5.2); Boxplots visualizations with altair Python package (version 4.2.0); Linear regression with statsmodels Python package (version 0.13.5). Differentially expressed genes was performed with DESeq272 (version 1.40.1) and R version 4.3.0 (2023-04-21 ucrt). We made publicly available the code at https://github.com/AIRMEC/HECTOR.

For manuscripts utilizing custom algorithms or software that are central to the research but not yet described in published literature, software must be made available to editors and reviewers. We strongly encourage code deposition in a community repository (e.g. GitHub). See the Nature Portfolio guidelines for submitting code & software for further information.

Data

Policy information about availability of data

All manuscripts must include a data availability statement. This statement should provide the following information, where applicable:

- Accession codes, unique identifiers, or web links for publicly available datasets
- A description of any restrictions on data availability
- For clinical datasets or third party data, please ensure that the statement adheres to our policy

The tumor material and datasets generated during or analyzed in this study are not publicly available due to restrictions by privacy laws. Data and tumor material from PORTEC-1, PORTEC-2, PORTEC-3, MST, the TransPORTEC study, are held by the PORTEC study group and the international TransPORTEC consortium. Data and tumor material from the Danish cohort are held by the coauthor of this article G.O. Data and tumor material from the UMCG cohort are held by the coauthors of this article H.N and M.B; LUMC by the co-authors N.H and T.B. Requests for sharing of all data and material should be addressed to the corresponding author within 15 years of the date of publication of this Article and include a scientific proposal. Depending on the specific research proposal, the TransPORTEC consortium (PORTEC-3 and TransPORTEC study) or the PORTEC study group (PORTEC-1, PORTEC-2, MST), or co-author G.O., H.N and M.B, or N.H and T.B, will determine when, for how long, for which specific purposes, and under which conditions the requested data can be made available, subject to ethical consent. Requests for data access will be processed within a 3-month timeframe. TCGA-UCEC images, mutational status and clinical data are publicly available via the cBioPortal65,66 for Cancer Genomics at https://www.cbioportal.org/study/clinicalData?id=ucec_tcga_pan_can_atlas_2018. mRNA-seq data of the TCGA-UCEC were downloaded from http://firebrowse.org/?cohort=UCEC.

Research involving human participants, their data, or biological material

Policy information about studies with human participants or human data. See also policy information about sex, gender (identity/presentation), and sexual orientation and race, ethnicity and racism.

Reporting on sex and gender	We do not report on sex and gender. The findings of this study relate to endometrial cancer and apply to biologically female individuals.<br>We reported in the methods "We included study participants of the female sex, independent of gender identity."
Reporting on race, ethnicity, or other socially relevant groupings	We do not report on race, ethnicity or socially relevant groupings, nor have data related to this.
Population characteristics	All population characteristics of any cohort used are described in the Supplemental Figure 2 and Supplemental Tables 1,2,14, in which we report the following characteristics : age, type of tumor, tumor stage, molecular characteristic of the tumor (POLE mutation, Mismatch repair deficient, p53 abnormality), adjuvant treatment received, and median follow-up time.
Recruitment	Cohorts used are the three PORTEC (1/2/3) randomized trials in which recruitment followed the design protocol of the clinical trials as reported in their original publication as well as in the Methods section. The PORTEC-1 trial recruited 714 women with early-stage intermediate risk EC from 1990 to 1997, and after primary surgery, randomly assigned to pelvic external beam radiotherapy or no adjuvant treatment. The PORTEC-2 trial randomized 427 women with early-stage high-intermediate risk EC between 2000 to 2006 to external beam radiotherapy or vaginal brachytherapy. The PORTEC-3 randomized trial included 660 women with stage I-III high risk EC from 2006 and 2013, and randomly allocated them to pelvic external beam radiotherapy alone or external beam radiotherapy combined with concurrent and adjuvant chemotherapy. The retrospective TransPORTEC study included 116 high-risk EC tumors from international patients using the same inclusion criteria as the PORTEC-3 from five institutions (Leiden University Medical Center, The Netherlands; University Medical Center Groningen, The Netherlands; University College London, United Kingdom; St Mary's Hospital, Manchester, United Kingdom; and Institute Gustave Roussy, Villejuif, France). The prospective cohort of Medisch Spectrum Twente (MST) included 257 patients with stage I-III high risk EC, with the same inclusion criteria as the PORTEC-3, who were treated between 1987 and 2015 at MST, Enschede in the Netherlands. The Danish cohort consisted of 451 high-grade EC of patients who were prospectively registered in the Danish gynecological cancer database. The Leiden cohort is a retrospectively collected population-based cohort of 222 patients diagnosed and treated at the Leiden University Medical Center between 2012 and 2021.<br>This study excluded patients if tumor data or material was missing such as an image of the tumor, or missing follow-up data.
Ethics oversight	The PORTEC-1, PORTEC-2 (NCT00376844) PORTEC-3 (NCT00411138) study protocols were approved by the Medical Ethical Committee Leiden – Den Haag – Delft and the medical ethics committees at participating centers. Studies were conducted in accordance with the principles of the Declaration of Helsinki. Ethical permissions for the retrospective use of the clinical trials and retrospective cohorts (TransPORTEC study, MST), were obtained by the Medical Ethical Committee Leiden – Den Haag – Delft (numbers B21.065 and B21.011) as well as the LUMC Cohort (nWMO-D4-2023-002), and the Danish Cohort by the Center for Regional Udvikling – De Videnskabsetiske Komiteer (H-16025909). All study participants of the clinical trials provided informed consent. The ethical boards have provided a waiver for informed consent for the other studies. For the UMCG cohort, the medical ethical committee granted permission of the use of the data and provided a waiver for informed consent due to the observational nature of the study.

Note that full information on the approval of the study protocol must also be provided in the manuscript.

Field-specific reporting

Please select the one below that is the best fit for your research. If you are not sure, read the appropriate sections before making your selection.

☒ Life sciences ☐ Behavioural & social sciences ☐ Ecological, evolutionary & environmental sciences

For a reference copy of the document with all sections, see nature.com/documents/nr-reporting-summary-flat.pdf

Life sciences study design

All studies must disclose on these points even when the disclosure is negative.

Sample size	Deep learning-based methods benefit from the largest possible datasets for a better training and relatively large test sets. No minimum sample size was calculated in our case as we possessed a sufficiently large cohort of 2,072 patients for training and testing the model. As for the split between training, validation and testing, we followed standard split by sampling 20% for internal test set, and held out two external test sets. As a result, the training dataset had 1,408 patients (with 246 clinical events), the internal test 353 patients (with 62 clinical events) and the first external test set 151 patients (with 24 clinical events) and the second test set contained 160 patients (with 14 events), which is sufficiently large for having enough events in each set and evaluating the accuracy of the model or using Kaplan-Meir's methods.
Data exclusions	Exclusion criteria were pre-established before development and testing of the models based on 1) requirements for training the model and 2) clinical knowledge of the disease based on previous publication. First exclusion criteria are the absence of tumor material or/and tumor data; artifacts in the digitized tumor slide such as out of focus areas. In our specific study, where the supervised Deep learning-based developed model is trained to predict distant recurrence-free probability, patients that already had distant recurrence at diagnosis (that is FIGO stage IV) and then the ones who received adjuvant chemotherapy were excluded from training-testing. This is because adjuvant chemotherapy likely reduces this risk as shown in previous clinical publications. Moreover in our dataset in which treatment is known for any patient, the far majority of patients treated with chemotherapy comes from the PORTEC-3 randomized trial and chemotherapy is not standard of care in the Netherlands, and rarely given. Therefore, any bias which would exclude a specific type of tumor is very unlikely, as a matter of fact, all the patients included in this study cover all tumor types, all stages I to III, and all molecular types. These specificities were all reported in the manuscript in the Methods as well as in the supplemental data with, for instance, a flow chart indicating exact number of patients being excluded.
Replication	We used 5 fold-cross validation. Furthermore we showed similar performance in 5 fold-cross validation, the internal and external test sets.
Randomization	The external test sets were blindly and randomly held-out. The internal test set was randomly sampled from the entire training set. Similarly, the 5 fold-cross validation split was performed randomly.
Blinding	Our manuscript describes the development and performance of a deep learning model. The developed model was tested one time after development, in one internal and one external test set and performance was reported. The tumor slide images of internal and external test sets were therefore completely unseen by the model, and no optimization on the internal nor external test set was performed. Furthermore, the internal and external test sets were blindly and randomly held-out. Specifically, tumor characteristics in each test set were not analyzed before testing the model performance in these test sets.

Reporting for specific materials, systems and methods

We require information from authors about some types of materials, experimental systems and methods used in many studies. Here, indicate whether each material, system or method listed is relevant to your study. If you are not sure if a list item applies to your research, read the appropriate section before selecting a response.

Materials & experimental systems

n/a	Involved in the study	
☒	☐	Antibodies
☒	☐	Eukaryotic cell lines
☒	☐	Palaeontology and archaeology
☒	☐	Animals and other organisms
☐	☒	Clinical data
☒	☐	Dual use research of concern
☒	☐	Plants

Methods

n/a	Involved in the study	
☒	☐	ChIP-seq
☒	☐	Flow cytometry
☒	☐	MRI-based neuroimaging

Clinical data

Policy information about clinical studies

All manuscripts should comply with the ICMJE guidelines for publication of clinical research and a completed CONSORT checklist must be included with all submissions.

Clinical trial registration	The PORTEC-1 (there is no registration as clinical trial registration did not exist in the 90s. The PORTEC-1 study was supported by the grant CKVO 90–01), PORTEC-2 (NCT00376844) PORTEC-3 (NCT00411138)

Study protocol

For the clinical trials that were included in this study, that is the PORTEC-1, PORTEC-2, PORTEC-3, we can provide the protocols as they are not available online. The PORTEC-1 protocol is in Dutch and the PORTEC-2 and PORTEC-3 in english.

Data collection

The PORTEC-1 trial recruited 714 women with early-stage intermediate risk EC from 1990 to 1997, and after primary surgery, randomly assigned to pelvic external beam radiotherapy or no adjuvant treatment. 19 departments of radiation oncology in the Netherlands took part. The patients were evaluated and treated by their local radiation oncologist. Central blocked randomisation by telephone was done at the Daniel den Hoed Cancer Centre trial office. The PORTEC-2 trial randomized 427 women with early-stage high-intermediate risk EC between 2000 to 2006 to external beam radiotherapy or vaginal brachytherapy. 19 Dutch radiation oncology departments participated. Patient details and answers about eligibility questions were entered by the data managers of the participating centres. Eligibility check and randomisation were done on the basis of the original pathology diagnosis. Central review of the pathology was done to assess histological type, stage, and grade. The PORTEC-3 randomized trial included 660 women with stage I-III high risk EC from 2006 and 2013, and randomly allocated them to pelvic external beam radiotherapy alone or external beam radiotherapy combined with concurrent and adjuvant chemotherapy. 103 centres (oncology centres, university hospitals, regional hospitals, or radiation oncology centres with referrals from regional hospitals) from six clinical trial groups which collaborated in the Gynaecological Cancer Intergroup. Participating groups were the National Cancer Research Institute (NCRI; UK), Australia and New Zealand Gynaecologic Oncology Group (ANZGOG; Australia and New Zealand), Mario Negri Gynaecologic Oncology Group (MaNGO; Italy), Canadian Cancer Trials Group (CCTG; Canada), and Fedegyn (France). Central pathology review was done by reference gynaecopathologists (as appointed by each participating group before the start of the trial) to determine final eligibility. The slides and blocks were sent to each participating group's central review pathologists at one gynaecological pathology review site (in France and Italy), two sites (in the UK and the Netherlands), or five to six sites (in Australia and New Zealand, and Canada), with the result of the review confirming the patient's eligibility for the trial being sent to the local investigators within 1 week.

Outcomes

This is a deep learning-based study. We predefined the primary outcomes as the performance of the model measured by the concordance-index between the predicted risk score of distant recurrence and the true time to distant recurrence. Secondary outcomes were the survival area under the curve (AUC) and the Brier scores. Additionally, we analyzed the distant recurrence-free probabilities and stratification with the Kaplan Meier's method, the log-rank test and Cox regression analyses.

Plants

Seed stocks

Report on the source of all seed stocks or other plant material used. If applicable, state the seed stock centre and catalogue number. If plant specimens were collected from the field, describe the collection location, date and sampling procedures.

Novel plant genotypes

Describe the methods by which all novel plant genotypes were produced. This includes those generated by transgenic approaches, gene editing, chemical/radiation-based mutagenesis and hybridization. For transgenic lines, describe the transformation method, the number of independent lines analyzed and the generation upon which experiments were performed. For gene-edited lines, describe the editor used, the endogenous sequence targeted for editing, the targeting guide RNA sequence (if applicable) and how the editor was applied.

Authentication

Describe any authentication procedures for each seed stock used or novel genotype generated. Describe any experiments used to assess the effect of a mutation and, where applicable, how potential secondary effects (e.g. second site T-DNA insertions, mosiacism, off-target gene editing) were examined.
